# Effect of prebiotics, probiotics, and synbiotics on gastrointestinal outcomes in healthy adults and active adults at rest and in response to exercise—A systematic literature review

**DOI:** 10.3389/fnut.2022.1003620

**Published:** 2022-12-07

**Authors:** Christopher E. Rauch, Alice S. Mika, Alan J. McCubbin, Zoya Huschtscha, Ricardo J. S. Costa

**Affiliations:** ^1^Department of Nutrition Dietetics and Food, School of Clinical Sciences, Faculty of Medicine Nursing and Health Sciences, Monash University, Notting Hill, VIC, Australia; ^2^Institute for Physical Activity and Nutrition, School of Exercise and Nutrition Sciences, Deakin University, Geelong, VIC, Australia

**Keywords:** exercise-induced gastrointestinal syndrome, running, exertional-heat stress, epithelial, permeability, endotoxin, cytokine, gastrointestinal symptoms

## Abstract

**Introduction:**

A systematic literature search was undertaken to assess the impact of pre-, pro-, and syn-biotic supplementation on measures of gastrointestinal status at rest and in response to acute exercise.

**Methods:**

Six databases (Ovid MEDLINE, EMBASE, Cinahl, SportsDISCUS, Web of Science, and Scopus) were used. Included were human research studies in healthy sedentary adults, and healthy active adults, involving supplementation and control or placebo groups. Sedentary individuals with non-communicable disease risk or established gastrointestinal inflammatory or functional diseases/disorders were excluded.

**Results:**

A total of *n* = 1,204 participants were included from *n* = 37 papers reported resting outcomes, and *n* = 13 reported exercise-induced gastrointestinal syndrome (EIGS) outcomes. No supplement improved gastrointestinal permeability or gastrointestinal symptoms (GIS), and systemic endotoxemia at rest. Only modest positive changes in inflammatory cytokine profiles were observed in *n* = 3/15 studies at rest. Prebiotic studies (*n* = 4/5) reported significantly increased resting fecal Bifidobacteria, but no consistent differences in other microbes. Probiotic studies (*n* = 4/9) increased the supplemented bacterial species-strain. Only arabinoxylan oligosaccharide supplementation increased total fecal short chain fatty acid (SCFA) and butyrate concentrations. In response to exercise, probiotics did not substantially influence epithelial injury and permeability, systemic endotoxin profile, or GIS. Two studies reported reduced systemic inflammatory cytokine responses to exercise. Probiotic supplementation did not substantially influence GIS during exercise.

**Discussion:**

Synbiotic outcomes resembled probiotics, likely due to the minimal dose of prebiotic included. Methodological issues and high risk of bias were identified in several studies, using the Cochrane Risk of Bias Assessment Tool. A major limitation in the majority of included studies was the lack of a comprehensive approach of well-validated biomarkers specific to gastrointestinal outcomes and many included studies featured small sample sizes. Prebiotic supplementation can influence gut microbial composition and SCFA concentration; whereas probiotics increase the supplemented species-strain, with minimal effect on SCFA, and no effect on any other gastrointestinal status marker at rest. Probiotic and synbiotic supplementation does not substantially reduce epithelial injury and permeability, systemic endotoxin and inflammatory cytokine profiles, or GIS in response to acute exercise.

## Introduction

Gastrointestinal disturbances and associated symptoms are relatively common occurrences in the general population, and range from minor inconvenience to severe clinical conditions (e.g., gastrointestinal inflammatory and functional diseases/disorders) (1). Athletes (i.e., elite and amateur) and recreationally active populations (i.e., health and fitness) are also susceptible to these gastrointestinal disturbances and symptoms, which include those occurring at rest, as well as substantial perturbations that occur specifically during and/or after exercise (2). The reported incidence of gastrointestinal symptoms (GIS), as a result of exercise, during and/or after competitive events varies from <5 to >85% in both the elite and recreational population (2), depending on the exertional extent of the event. It is now well established that various factors increase the magnitude of exertional stress, and subsequently increase the risk of substantial gastrointestinal disturbances and associated GIS. These extrinsic and intrinsic exacerbation factors have been described in Costa et al. (2, 3).

**Figure 1:**
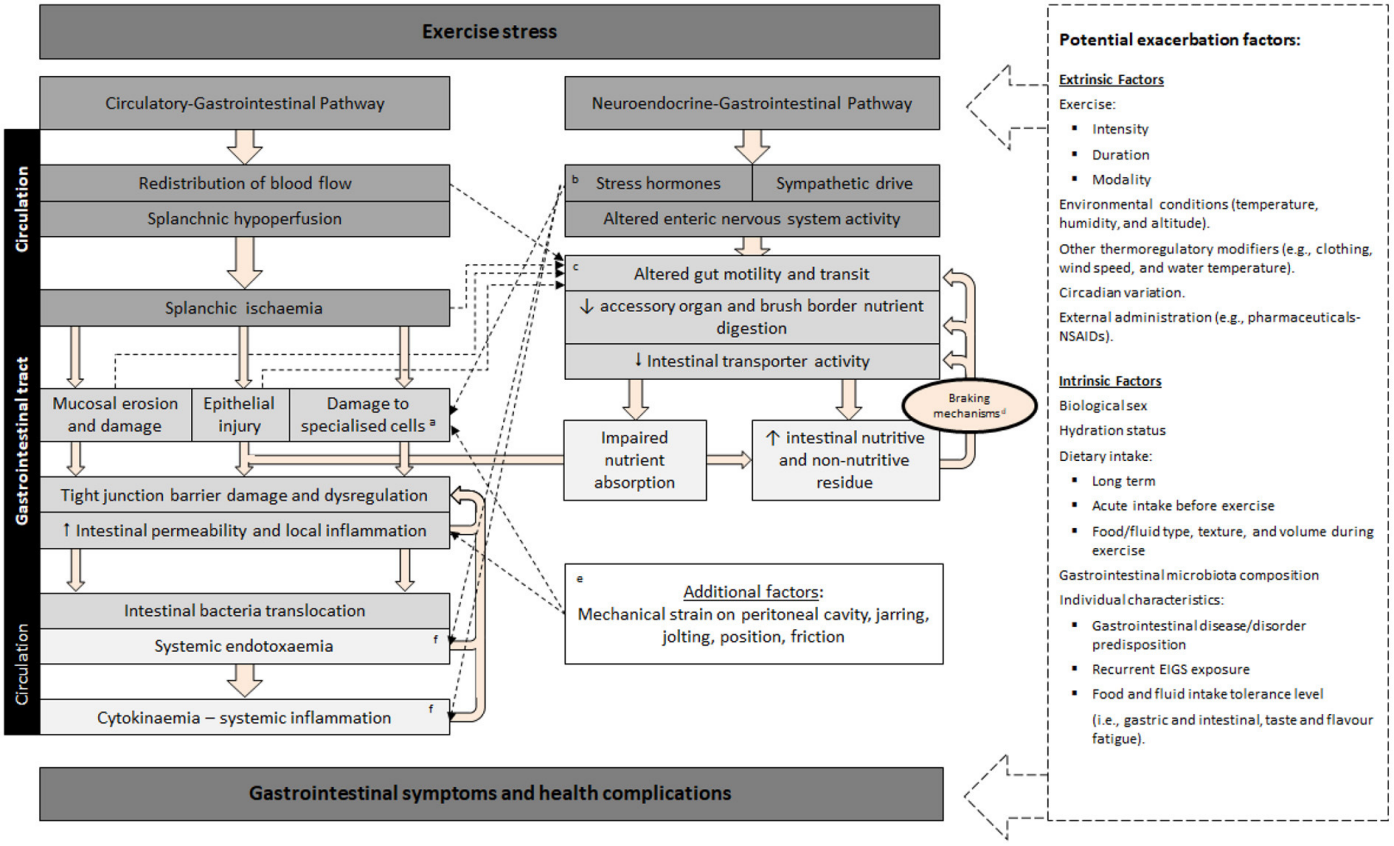
Schematic description of exercise-induced gastrointestinal syndrome (EIGS): Physiological changes in circulatory and neuroendocrine pathways at the onset of exercise resulting in perturbed gastrointestinal integrity and function, which may lead to gastrointestinal symptoms, with performance and clinical implications (2, 3). ^a^Specialized antimicrobial protein-secreting (i.e., Paneth cells) and mucus-producing (goblet cells) cells, aid in preventing intestinal-originating pathogenic microorganisms entering systemic circulation. ^b^Splanchnic hypoperfusion and subsequent intestinal ischemia and injury (including mucosal erosion) results from stress induced direct (e.g., enteric nervous system, and/or enteroendocrine cell) or indirect (e.g., braking mechanisms) alterations to gastrointestinal motility. ^c^Increase in neuroendocrine activation and suppressed submucosal and myenteric plexus result in epithelial cell loss and subsequent perturbed tight junctions (4, 5). ^d^Gastrointestinal brake mechanisms: Nutritive and non-nutritive residue along the small intestine, and inclusive of terminal ileum, results in neural and enteroendocrine negative feedback to gastric activity (6–10). ^e^Aggressive acute or low grade, prolonged mechanical strain, is proposed to contribute toward disturbances to epithelial integrity (i.e., epithelial cell injury and tight-junction dysregulation) and subsequent “knock-on” effects for gastrointestinal functional responses (11). ^f^Bacteria and bacterial endotoxin microorganism molecular patterns (MAMPs), and stress induced danger associated molecular patterns (DAMPs), are proposed to contribute toward the magnitude of systemic immune responses (e.g., systemic inflammatory profile) (12). Adapted from Costa et al. (2), with permission.

The pathophysiology of disturbances to gastrointestinal integrity, function, subsequent systemic responses (e.g., endotoxemia and systemic inflammation), and associated GIS that active individuals present in response to exercise is referred to as “exercise-induced gastrointestinal syndrome” (EIGS), and is characterized by two primary pathways (Figure 1), as described in Gaskell et al. (13). Briefly, the gastrointestinal-circulatory pathway describes the splanchnic hypoperfusion and intestinal ischemia that occurs due to a redistribution of blood flow to skeletal muscle and peripheral circulation (14, 15), resulting in intestinal epithelial injury and hyperpermeability, plus local and/or systemic inflammatory effects in response to translocated pathogens (16–18). The gastrointestinal-neuroendocrine pathway describes the stress response contribution to gastrointestinal integrity and functional disturbances, *via* an increase in stress hormone responses and sympathetic activation (2). Such stress response is synonymous with impaired gastrointestinal motility, transit, digestive function, and nutrient absorption (19–21).

It is commonly assumed by athletes and their support crew that administration of probiotics will confer benefits to the gastrointestinal tract, particularly at times of intensified training or leading into or during competition, when gastrointestinal disturbance is of particular concern due to the potential to compromise physical performance (6, 19). Recently published narrative or opinion piece reviews exploring prebiotics (i.e., non-digestible material that can be fermented by bacteria in the lower gastrointestinal tract), probiotics (i.e., live bacteria which survive transit to colonize the lower gastrointestinal tract), and synbiotics (a combination of pre- and pro-biotics) in active adults, have implied a beneficial effect on the gastrointestinal tract in response to exercise and improved performance; however the primary focus has been on exercise performance or immunological outcomes (22–29). Unlike these narrative or opinion-based reviews, recent systematic literature reviews (SLR) that focused and/or included pro- and/or syn-biotic supplementation, concluded inconsistent methodologies and/or findings that provided no convincing evidence of any substantial beneficial effects resulting from probiotic supplementation in healthy populations (30–32). Although it is important to note that these SLR did not comprehensively evaluate EIGS markers or changes to fecal bacterial taxa or SCFA. Nevertheless, it has recently been demonstrated that the microbial composition of the gastrointestinal tract, when using partial correlation analysis and controlling for potential confounding factors, is another factor that may influence an individual's susceptibility of developing EIGS and associated GIS (33). Exploratory work suggests that an increased relative abundance of various SCFA producing commensal bacterial groups may improve epithelial integrity and reduce GIS in response to prolonged strenuous exercise (33–36), through mechanisms that warrant further exploration and clarification. Proposed mechanisms may include: (i) attenuation of exercise-associated hypoperfusion through the presence of nutrient content along the gastrointestinal tract and increased fermentation activity of commensal bacteria (6, 19, 37, 38); and/or, (ii) increased epithelial cell stability resulting from an increased luminal SCFA concentration (34, 35). The possible role of the gut microbiota as an intrinsic factor that alters the risk of EIGS pathophysiology, and subsequent GIS in response to exercise, suggests potential to manipulate this risk through the use of prebiotics, probiotics, and synbiotics. Mechanistically, particular probiotics (e.g., *Lactobacillus plantarum*) have demonstrated favorable effects on epithelial integrity (39, 40) and in clinical outcomes for patients presenting with infection and/or inflammation (41–43).

In regard to the application of variables to assess the impact of pre-, pro-, and/or syn-biotic supplementation on gastrointestinal status in response to exercise stress, various biomarkers have been employed (44). Changes in gastrointestinal integrity as a result of EIGS are commonly reported in research studies using intestinal fatty acid binding protein (I-FABP), a surrogate marker for intestinal epithelial injury; or urinary or plasma claudin-3 concentration, a surrogate marker for epithelial tight gap junction function or injury. Gastrointestinal permeability is commonly assessed by dual or multiple sugars tests including, urinary lactulose:mannitol or lactulose:rhamnose ratio for small intestinal permeability, and sucrose for gastroduodenal permeability. Translocation of pathogenic agents from the gastrointestinal lumen into systemic circulation are observed by measuring the plasma endotoxin response, including lipopolysaccharide (LPS), resulting lipopolysaccharide binding protein (LBP) response, and/or gram-negative endotoxin and anti-endotoxin antibodies such as IgM. Objective assessment of perturbations to gastrointestinal function may be measured *via*: (i) gastric antral sonography for gastric emptying, measuring ultrasound half gastric emptying time or ultrasound full emptying time (45); (ii) electrogastrography (EGG), recording gastric myoelectrical activity (e.g., slow waves) using electrodes placed on the abdominal skin (21); and/or (iii) breath hydrogen response as a measure of carbohydrate malabsorption, as undigested material pass through the ilium where bacterial fermentation releases hydrogen and methane which diffuse through the lumen into the blood and excreted *via* the lungs (19, 46–49). *Via* the latter mechanism, orocecal transit time (OCTT) can be indirectly assessed *via* the administration of an indigestible carbohydrate, such as lactulose, recording the time to the resulting breath hydrogen peak (20). Participant reported data on defecation frequency and stool consistency using the Bristol Stool Rating Scale also offer supportive evidence on changes to gastrointestinal function (50).

As stated, several SLR have been published in respect to biotic supplementation interventions in active adults, but none have considered the methodological issues (e.g., adequate experimental design including exertional or exertional-heat stress with or without issues with sample collection timing, experimental control of confounding factors, limitations in EIGS biomarkers, validation and reliability of GIS assessment tool) or magnitude of response of key pathophysiological markers (e.g., clinical significance of responses) (44). To date, no systematic review has comprehensively examined in-depth the effect of short or long-term pre-, pro- and syn-biotics supplementation on gastrointestinal status outcomes in healthy active adults at rest and in response to acute exercise. Therefore, the aim of this current systematic literature review is to determine the beneficial, detrimental, or neutral effects of differing supplementation periods and dosages of pre-, pro- and syn-biotic supplementation, taken by healthy active adults, on gastrointestinal outcomes at rest and in response to exercise, with a specific focus on the defined markers characteristic of EIGS and associated GIS.

## Methods

A systematic literature search was performed by three researchers (A.J.M, C.R, and Z.H), to determine the impact of varying pre-, pro-, and syn-biotic supplements and supplementation period on markers of gastrointestinal integrity (i.e., intestinal epithelial injury, permeability, and bacterial endotoxin translocation), gastrointestinal functional responses (i.e., gastric emptying, gastrointestinal transit, and myoelectrical activity), systemic inflammatory responses, gastrointestinal symptoms (i.e., incidence, severity, stool frequency, and consistency), and variables relating to the gut microbiota (i.e., bacterial composition and SCFA profile), both at rest and in response to exercise. The review was completed in accordance with the Preferred Reporting Items for Systematic Review and Meta-Analyses (PRISMA) statement (51). The review was not pre-registered.

### Search strategy

The literature search was undertaken of English-language, original research studies, from inception to beginning March 2022, using the databases Ovid MEDLINE, EMBASE, Cinahl, SportsDISCUS, Web of Science, and Scopus. Reference lists of review papers found from the search, and others known to the authors, were searched to identify any studies missed in the original search. Keywords applied in the literature search are shown in Table 1A, with search strategy logic for each database shown in Table 1B.

**Table 1:** General search strategy (A) and search strategy logic by database (B) for the systematic review on the effect of pre-, pro-, and synbiotics on gastrointestinal outcomes in healthy adults and healthy active adults.

**(A) Field one (combine with OR): Population**		**Field two (combine with OR): Intervention and comparison**		**Field three (combine with OR): Outcome**
Keywords: Exercise, Run^*^, Cycling, Cyclist, Physical Activity	AND	Keywords: probiotic, prebiotic, synbiotic	AND	Keywords:, intestinal injury and damage, I-FABP, intestinal fatty acid, tight junction, mucosal barrier, zonulin, claudin, endotoxin, LPS, LAL, lipopolysaccharide, gram negative bacteria, LBP, sCD14, intestinal permeability, lactulose, rhamnose, mannitol, urinary sugars, gastrointestinal motility, OCTT, EGG, gastrointestinal symptoms, gut discomfort, short chain fatty acids, SCFA, gastrointestinal microbiota and microbial composition.
**(B) OVID EMBASE and Ovid MEDLINE(R) and Epub Ahead of Print**	
1.	(Exercise or Run^*^ or Cycling or Cyclist or “Physical Activity”).mp. (mp = title, abstract, heading word, drug trade name, original title, device manufacturer, drug manufacturer, device trade name, keyword heading word, floating subheading word, candidate term word)
2.	(probiotic or prebiotic or synbiotic).mp. (mp = title, abstract, heading word, drug trade name, original title, device manufacturer, drug manufacturer, device trade name, keyword heading word, floating subheading word, candidate term word)
3.	(“intestinal injury” or “intestinal damage” or I-FABP or “intestinal fatty acid” or “tight junction” or “mucosal barrier” or zonulin or claudin or endotoxin or LPS or LAL or lipopolysaccharide or “gram negative bacteria” or LBP or sCD14 or “intestinal permeability” or lactulose or rhamnose or mannitol or “urinary sugars” or “gastrointestinal motility” or OCTT or EGG or “gastrointestinal symptoms” or “gut discomfort” or “short chain fatty acid^*^” or SCFA or microbiota or “microbial composition”).mp. (mp = title, abstract, heading word, drug trade name, original title, device manufacturer, drug manufacturer, device trade name, keyword heading word, floating subheading word, candidate term word)
4.	1 and 2 and 3
**SCOPUS**	
	(TITLE-ABS-KEY (exercise OR run^*^ OR cycling OR cyclist OR {physical activity}) AND TITLE-ABS-KEY (probiotic OR prebiotic OR synbiotic) AND TITLE-ABS-KEY ({intestinal injury} OR {intestinal damage} OR i-fabp OR {intestinal fatty acid} OR {tight junction} OR {mucosal barrier} OR zonulin OR claudin OR endotoxin OR lps OR lal OR lipopolysaccharide OR {gram negative bacteria} OR lbp OR scd14 OR {intestinal permeability} OR lactulose OR rhamnose OR mannitol OR {urinary sugars} OR {gastrointestinal motility} OR octt OR egg OR {gastrointestinal symptoms} OR {gut discomfort} OR {short chain fatty acid^*^} OR scfa OR microbiota OR {microbial composition}))
**CINAHL plus and SPORTDiscus with full text**	
S7	S1 AND S2 AND S6
S6	S3 OR S4 OR S5
S5	gut discomfort OR short chain fatty acid^*^ OR SCFA OR microbiota OR microbial composition
S4	gram negative bacteria OR LBP OR sCD14 OR intestinal permeability OR lactulose OR rhamnose OR mannitol OR urinary sugars OR gastrointestinal motility OR OCTT OR EGG OR gastrointestinal symptoms
S3	intestinal injury OR intestinal damage OR I-FABP OR intestinal fatty acid OR tight junction OR mucosal barrier OR zonulin OR claudin OR endotoxin or LPS or LAL or lipopolysaccharide
S2	probiotic OR prebiotic OR synbiotic
S1	exercise OR run^*^ OR cycling OR cyclist^*^ OR physical activity
**Web of science**	
	Exercise OR run^*^ OR cycling OR cyclist OR “physical activity” (Topic) and probiotic or prebiotic or synbiotic (Topic) and “intestinal injury” or “intestinal damage” or I-FABP or “intestinal fatty acid” or “tight junction” or “mucosal barrier” or zonulin or claudin or endotoxin or LPS or LAL or lipopolysaccharide or “gram negative bacteria” or LBP or sCD14 or “intestinal permeability” or lactulose or rhamnose or mannitol or “urinary sugars” or “gastrointestinal motility” or OCTT or EGG or “gastrointestinal symptoms” or “gut discomfort” or “short chain fatty acid^*^” or SCFA or microbiota or “microbial composition” (Topic)

* Used to retrieve unlimited suffix variations.

### Eligibility criteria

**Figure 2:**
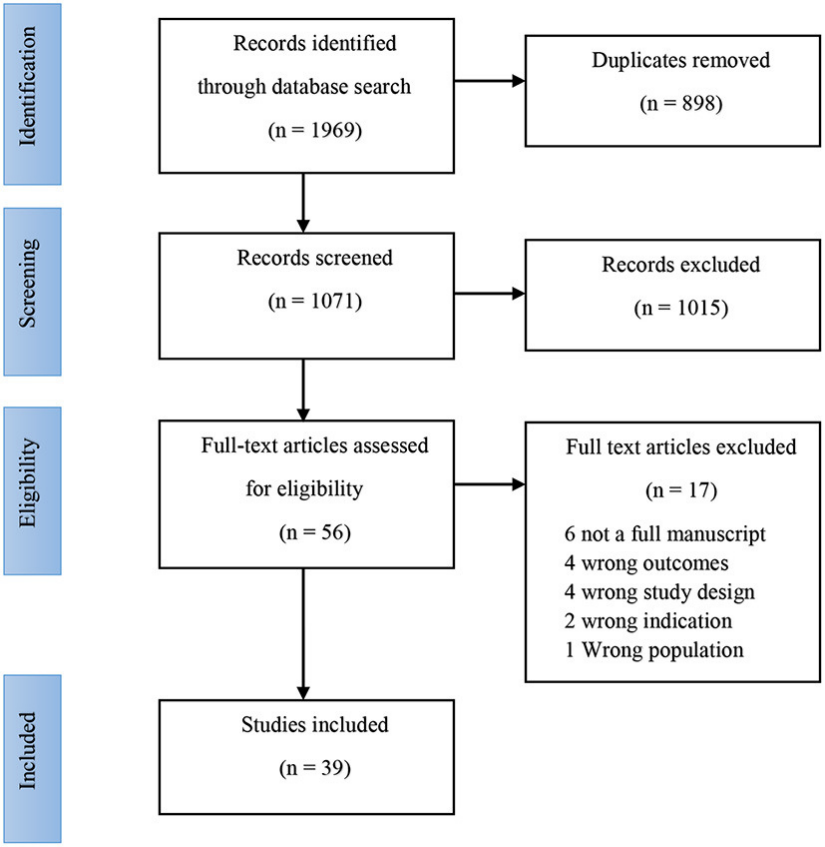
PRISMA diagram, showing the inclusion and exclusion of papers in the review.

Eligibility criteria were established a priori as per the Participant Intervention Comparator Outcomes Study (PICOS) design format (Table 2) (52). Original human research studies in healthy sedentary adults, and healthy active adults, involving supplementation and control or placebo groups, reporting quantified data on EIGS outcomes *in vivo* (i.e., gastrointestinal symptom description, stool frequency and consistency, intestinal integrity and permeability, systemic endotoxin and/or inflammatory cytokine profiles, gastrointestinal motility and/or other functional responses, fecal bacterial taxa and SCFA concentration) were considered for inclusion. Exclusion criteria included sedentary individuals with non-communicable disease risk or established gastrointestinal inflammatory or functional diseases/disorders, populations undergoing dietary modifications and/or supplementation, other than the pre-, pro-, or syn-biotic intervention, and a lack of a placebo or a control group. The inclusion and exclusion criteria were cross checked against the criteria reported within the reviewed studies. *Ex vivo* outcomes (i.e., antigen stimulated cytokine responses or other blood or tissue cultures) were excluded. After removal of duplicates, study titles and abstracts were reviewed by two researchers (Z.H and C.R) against the eligibility criteria, and verified by a third researcher (A.J.M) when required (i.e., disagreement between the primary reviewers) (Figure 2).

**Table 2:** PICOS table, showing the inclusion and exclusion criteria for study population, intervention, comparator, outcome/s, and study design.

**PICOS**	**Inclusion**	**Exclusion**
Population	Human Healthy community dwelling sedentary individuals. Sedentary individuals initiating a structured physical activity or exercise program. Recreational and competitive active adults (18–60 years). Male and female biological sex.	Animals and *in vitro* studies. Infants or children. Pregnancy or lactating. Sedentary individuals with non-communicable disease risk or established disease (i.e., cardiometabolic risk factors or established cardiovascular diseases, diabetes mellitus, and/or metabolic syndrome). Diagnosed disease or syndrome states (i.e., all clinical populations). Population adhering to dietary modifications and/or dietary supplementation, other than pre-/pro-/syn-biotic intervention.
Intervention	Acute and prolonged provisions of prebiotic/s, probiotic/s, and synbiotic blends (i.e., prebiotic + probiotic, with or without other nutrient inclusion) (e.g., vitamins, minerals, lipids, phytochemicals, and/or volatiles). With and without monitored and/or structures physical active and/or exercise program. Dietary control (monitoring or provisions).	Dietary interventions not containing acute and prolonged provisions of prebiotic/s, probiotic/s, and synbiotic blends. Acute and prolonged provisions of prebiotic/s, probiotic/s, and synbiotic blends that contain a pharmaceutics grade product or compound.
Comparator	Placebo group Control group	No placebo or control
Outcome	Gastrointestinal integrity markers: e.g., I-FABP, Claudin-3, dual sugars test for permeability, and other markers proposed to assess gastrointestinal epithelial integrity. Gastrointestinal functional markers: e.g., gastric aspiration, C^13^ breath test, OCTT, EGG, pH pill monitoring, H_2_ and CH_4_ malabsorption challenge. Systemic markers of compromised gastrointestinal integrity: e.g., CRP, systemic inflammatory response cytokine profile, systemic endotoxin profile (e.g., LPS, gram-negative endotoxin, ant-endotoxin antibody, sCD14, and/or LBP), systemic microbial identification (e.g., gene sequencing determination), immune cell functional responses and/or counts. Gastrointestinal signs and symptoms: e.g., stool habits and texture, QoL, and/or symptoms. Gastrointestinal microbiota: e.g., bacterial taxonomy (ASV or OTU) including α-diversity and relative abundance, bacterial functional markers including SCFA concentration (e.g., butyrate, propionate, and/or acetate).	
Study design	RCT or randomized crossover trial.	All other study designs

ASV, Amplicon sequence variant; CRP, c-reactive protein; EGG, electrogastrography; I-FABP, Intestinal fatty acid binding protein; LBP, lipopolysaccharide binding protein; LPS, lipopolysaccharide; OCTT, orocecal transit time; OTU, operational taxonomic units; QoL, quality of life; sCD14, soluble CD14; SCFA, short chain fatty acid; RCT, randomized control trial.

### Data extraction

Data was extracted by two researchers (C.R. and Z.H.) and cross-checked by a third (A.J.M.). The extracted variables included the number of participants, sample size determination, age, biological sex, training status (i.e., years of experience and *V*O_2max_ where available), intervention (i.e., quantity, food source and composition of prebiotic, and/or bacterial species/strain of probiotic), exercise protocol used where relevant, ambient conditions, physiological and thermoregulatory strain responses during the exercise protocol where relevant). EIGS outcomes extracted included concentrations of: I-FABP (blood), claudin-3 (urinary or blood), cytokine response (blood), dual sugars (urinary or blood) endotoxin response (blood LPS, LPB, gram negative endotoxin and anti-endotoxin antibodies). Gastrointestinal function measures extracted included: gastric antral sonography, EGG, breath hydrogen response for malabsorption, and OCTT. Other functional measures extracted include defecation frequency, stool consistency and GIS. Timepoints for data included resting pre- and post-exercise (i.e., immediately post-exercise or short-term recovery) where reported. Other timepoints reported were included and clearly specified, where resting pre- and post-exercise timepoints were not reported. Pre- to post-exercise Δ were calculated from extracted data. Between group differences and within group differences were extracted and tabulated. Where no baseline data was reported, this was included, but clearly specified. Data from tables was extracted and tabulated as presented. Graphical data was measured using WebPlotDigitizer (53) where appropriate. Only meaningful data was extracted, with heat mapping and other unclear data presentation methods excluded from extraction. Data was again checked during extraction, and inclusion/exclusion criteria applied as appropriate. Disagreements between the primary reviewers were resolved by discussion and consensus. Data was separated into two groups according to the study protocol; outcomes measured only at rest after a period of supplementation, and outcomes measured in response to acute exercise, also following a period of supplementation. Due to the heterogeneous nature of the interventions, study methodologies and outcome measures, data were not considered appropriate for meta-analysis. Despite the lack of a meta-analysis, certainty of evidence was not deemed necessary as clinical or policy recommendations have not been made and the limitations of the data and findings have been discussed at length.

### Risk of bias assessment

A risk of bias assessment was undertaken for identified studies, using the Cochrane Risk of Bias Assessment Tool (54). The tool is used to assess the likelihood of selection bias (i.e., random sequence generation and allocation concealment), performance bias (i.e., adequacy of participant blinding), detection bias (i.e., adequacy of researcher blinding), attrition bias (i.e., completeness of outcome data), reporting bias (completeness of outcome reporting), and other potential forms of bias.

## Results

### Search result

Results of the literature search are shown in Figure 2. The initial search returned 1,969 individual records, with 898 duplicates removed. No additional records were found from the reference lists of recent review papers on the topic. Title and abstract screening excluded 1,015 records, with full text screening excluding an additional 17 records. Data was therefore extracted from 39 records, and further categorized based on available outcome data. Outcomes are reported from a total of 1,204 participants. Due to the multifaceted nature of gastrointestinal status at rest and in response to exercise, results are presented according to each outcome type; including direct or indirect markers of intestinal injury and/or permeability, systemic endotoxin and/or inflammatory cytokine concentration, gastrointestinal functional responses, luminal microbial composition and SCFA concentration. Studies assessing resting gastrointestinal status to a period of pre-, pro-, and syn-biotic supplementation, *n* = 37 papers reported at least one of the gastrointestinal outcomes at rest, with or without GIS, before and after the biotic intervention period (Table 3). Of these, *n* = 8 provided a prebiotic intervention, *n* = 24 a probiotic intervention, and *n* = 5 a synbiotic intervention. Of the prebiotic studies, *n* = 1 provided the intervention substance in capsules, while all others incorporated the prebiotic ingredient into specifically formulated foods (i.e., bread, pasta, snack bar, or non-carbonated soft drink). Probiotic interventions varied from one to eight bacterial strains, given either in capsules, fermented dairy-based food or beverage, or sachets containing powder to be mixed in water. Synbiotic interventions consisted of either capsules, dairy based food, beverage or powder, containing between two to four probiotic strain mixtures, supplemented with either one or two prebiotic ingredients. The supplementation period ranged from 1 to 16 weeks. All of the included studies were conducted with adult participants (mean or median age < 45 years). *N* = 21 of the *n* = 37 studies characterized a specific exercise or sporting background in participants (i.e., rugby union, soccer, cycling, swimming, baseball, distance running, triathlon, or participants taken from a combination of team, endurance, and racquet sports). *N* = 2 studies were conducted with military recruits undergoing intense military training.

**Table 3:** Systematic review search results and study characteristics of included studies that attempted to determine the impact of prebiotic, probiotic and synbiotic supplementation on gastrointestinal outcomes at rest.

**References**	**Population and study design**	**Sample size determination**	**Supplementation protocol (vs. placebo or control)**	**Dietary control (DC)** ** Physical activity (PA)**	**Outcome/s reported**
**Prebiotic studies**					
Damen et al. (55)	*N* = 27 (10 M and 17 F), age: 25 (IQR 23–29) years, activity/fitness: not stated, study design: RXT	Not specified	Bread fortified with xylanase to produce Arabinoxylan oligosaccharides (AXOS) on baking. 180 g bread (7.2 g arabinoxylan) daily for 3 wk.	DC: Usual diet, not monitored. PA: Physical activity not stated	Gastrointestinal function GIS Bacterial taxa Fecal SCFA
Finegold et al. (56)	*n* = 32 (11M/21F), age (M 23–34 yrs, F 21–49 yrs) (activity not stated) RCT	Not specified	Xylooligosaccharides (XOS), up to 350 mg per cap. High (2.8 g) or low (1.4 g) dose from capsules, daily for 8 wk	Instructed to avoid high XOS/FOS/GOS foods and pre/probiotics and fermented foods. Twenty-four hour dietary recall used to compare between groups (data not reported).	Bacterial taxa Fecal SCFA GIS
François et al. (57)	*n* = 63 (M33/F30), age 42 yrs (activity not stated) RXT	Based on dataset of an earlier human intervention trial with WBE (58), an evaluable sample size of *n* = 40 was expected to provide 80% power (two-sided, α = 0·05) for detecting statistically significant difference in fecal bifidobacterial levels (primary outcome variable) between treatments.	Non-carbonated soft drink with wheat bran extract [containing Arabinoxylan oligosaccharides (AXOS)]. High (8 g/d) or Low (2.4 g/d) dose AXOS taken in a 70 mL drink, twice daily for 3 wk	Usual diet, 3 meals/day pattern, no pro/prebiotics. All food and beverage intake recorded for first 3 days of each study period. No activity monitoring stated.	Bacterial taxa Fecal SCFA GIS
Kleessen et al. (59)	*n* = 45 (10M/35F), age 23.5 yrs (activity not stated) RCT	Not specified	Snack bar with chicory inulin (CH) or Jerusalem artichoke inulin (JA), 7.7 g fructans per bar. 1 bar/d for 1 wk, 2 bars/d for 2 wk.	Asked to maintain usual habits (not monitored)	Bacterial taxa Fecal SCFA Gastrointestinal function GIS
Reimer et al. (60)	*N* = 48 (22M/28F at recruitment) Age 31.2 ± 9.1/30.5 ± 8.6 (Trial 1/ 2) (healthy untrained) RXT	The study was powered on the primary outcome of change in gut microbiota, which for the purposes of sample size calculation was based on changes in fecal Bifidobacterium from a previous trial (59). A sample size of *n* = 25 per group was determined to have 80% power to detect a difference between groups in fecal Bifidobacterium counts (CFU/g) assuming a 1.10-log difference with SD = 1.22 log, an α = 0.05, and a dropout rate of ~25%.	Snack bar with inulin type fructans (ITF) Int 1: moderate dose 7 g/d inulin + 2 g wheat based fiber Int 2: low dose 3 g/d inulin + 2 g oat based fiber Con 1: snack bar (0 g inulin, 0 g fiber) Con 2: snack bar (0 g inulin, 1 g fiber) 1 bar/d for 4 wk.	3 day weighed food record. Energy (kcal), protein, carbohydrate, fat @ baseline and within trials^NS^ Fiber time × treatment, trial 1 (*P* = 0.001), trial 2 (*P* = 0.019). Exercise across both trials, bw or within-group differences^NS^ (data not shown).	Bacterial taxa Fecal SCFA GIS Gastrointestinal function
Russo et al. (61)	*n* = 15 males, age 18.8 ± 0.7 yrs (activity not stated) RXT	Not specified	Inulin-enriched pasta: Int: 11.0 g/d fructans Pla: 1.4 g/d fructans 100 g/day pasta for 5 wk	All food provided, low inulin, amount calculated according to individual requirements. No activity monitoring stated.	Gastrointestinal function GIS
Russo et al. (45)	*n* = 20 males, age 18.8 ± 0.7 yrs (activity not stated) RXT	Not specified	Inulin-enriched pasta: Int: 11.0 g/d fructans Pla: 1.4 g/d fructans 100 g/day pasta for 5 wk	All food provided, low inulin, amount calculated according to individual requirements. No activity monitoring stated.	Gastrointestinal function
Russo et al. (62)	*n* = 20 males, age 18.8 ± 0.7 yrs (activity not stated) RXT	Sample size calculations based on data from Russo et al. (45) and Russo et al. (61). Probability that study would detect treatment difference with a 2-sided 0.05 significance level equal to 80% required enrolling only 17 subjects. This assumed true difference between treatments of 20% of urinary recovery of La, and standard deviation of the difference of 27%.	Inulin-enriched pasta: Int: 11.0 g/d fructans Pla: 1.4 g/d fructans 100 g/day pasta for 5 wk	All food provided, low inulin, amount calculated according to individual requirements. No activity monitoring stated.	Intestinal permeability
**Probiotic studies**					
Axelrod et al. (63)	*n* = 7 endurance runners, VO_2max_ 57.9 mL/kg/min RXT	A priori power analysis based on a previous investigation from healthy runners (64) estimated ~6 needed to obtain statistical power at the recommended 0.80 level based upon mean, between-groups comparison effect size (*d* = 1.2).	*L. salivarius* UCC118, 2 × 10^8^ CFU/cap 1 capsule daily for 4 wk	Normal lifestyle Activity not monitored	Intestinal permeability Cytokine responses Bacterial taxa
Batatinha et al. (65)	*n* = 27 male marathon runners, age: Int: 35.96 ± 5.81; Pla: 40.46 ±7.79 Fitness status not stated RCT	Not specified	*B. animalis. Lactis 10 × 10^9^;* *L. Acidophilus 10 × 10^9^* 1 sachet daily for 30 days	Dietary control not stated. Training volume monitored, ^NS^ between groups.	Cytokine responses
Burton et al. (66)	*n* = 13 males age 24 (22–27) yrs (activity not stated) RXT	The desired sample number could not be determined because of the absence of previous clinical studies with a similar intervention.	Yogurt containing: *S. thermophilus* (10^9^ CFU/g), *L. delbrueckii* spp. *bulgaricus* (10^8^ CFU/g) *L. rhamnosus GG* (10^7^ CFU/g) 400 g daily for 2 wk	Avoid dairy and fermented foods, 3 d food record each trial. 3 d control diet (provided) before each test day. Usual activity. Avoid intense activity 3 d prior to testing. ^NS^ difference between groups	Endotoxin responses Cytokine responses Bacterial taxa
Carbuhn et al. (67)	*n* = 17 female swimmers, age and fitness status not stated RCT	Not specified	*B. longum* 35624, 1 × 10^9^ CFU bacteria per capsule 1 capsule daily for 6 wk	Refrain from foods rich in probiotics (ex. Kefir) and caffeine. Three day food log (^NS^between groups). Standardized swim training program	Endotoxin responses Cytokine responses
Gill et al. (68)	*n* = 8 male runners and triathletes Age: 26 yrs VO_2max_ 59 ml/kg/min RXT	Based on the typical standard deviation of 0.7 EU/ml for circulatory endotoxin responses to exertional-stress (16, 69), and using standard alpha (0.05) and beta values (0.8) www.dssresearch.com), a sample size of *n* = 8 is estimated to provide adequate statistical precision to detect a >10% difference in circulatory endotoxin concentration in response to EHS in the target population.	*L. casei*, 1.0 × 10^11^ cells/bottle Commercial supplement, taken twice daily for 7 days	Dietary recall. ^NS^ between groups. Activity control not stated.	Cytokine responses Endotoxin responses
Gleeson et al. (70)	*n* = 58 (54M/30F recruited) athletes Age: Int: 32 ± 14, Pla: 25 ± 9 yrs Weekly training load: Int: 8.7 ± 4.1 h/week Pla: 9.3 ± 3.8 h/week RCT	Sample-size based on expected rate of 2.0 ± 1.0 URTI episodes (M ± SD) (71), a target 30% reduction in number of episodes, statistical power of 80%, and Type I error of 5%.	Fermented milk with L. casei Shirota, 6.5 × 10^9^ live cells/drink 65 ml drink twice daily for 16 wk	Diet not monitored, no other probiotics or fermented dairy products. Training validated by International Physical Activity Questionnaire, ^NS^between groups	Cytokine responses GIS
Haywood et al. (72)	*n* = 30 male rugby union players, age 24.7 ± 3.6 yrs RCT	In order to detect a 30% reduction in the number of infected days with 80% power and type 1 error of 5%, *n* = 25 participants were required.	Probiotica P3, Nutra-life: *L. gasseri*, 2.6 × 10^9^ CFU/cap *B. bifidum*, 0.2 × 10^9^ organisms/cap *B. longum*, 0.2 × 10^9^ organisms/cap 1 capsule daily for 4 wk	Dietary control not stated. Standardized training program.	GIS
Hoffman et al. (73)	*n* = 15 male military recruits, age: Int: 20.0 ± 0.6, Pla: 20.2 ± 0.6 Fitness status not stated; military training. RCT	Because of the small sample size per group, it was decided a priori to initially analyze PRE-to-POST changes (Δ scores) using the non-parametric Mann-Whitney U test. In addition, to make additional inferences on the true effect of the dietary intervention, and account for the small sample size per group, magnitude-based inferential analysis was also used.	Staimune, Kerry Inc. (St Paul, MN). *Inactivated Bacillus coagulans; 1.0 × 10^9^ CFU (powder form) Daily for 2 wk*	Participants were not permitted to use any additional dietary supplementation. All soldiers consumed their meals together, maintaining a similar dietary intake throughout the study. During study period, soldiers were garrisoned on base and participated in the same training tasks that included hand-to-hand combat skill development, working with and against various weapons and physical conditioning 5 times a week.	Cytokine responses
Huang et al. (74)	*n* = 20 male triathletes Age: Int: 21.6 ± 1.3 Pla: 21.9 ± 1.4 VO_2max_ mL/kg/min): Int: 55.5 ± 8.6 Pla: 56.6 ± 9.0 RCT	Not specified	*L. plantarum PS128, 2 capsules; 1.5 × 10^10^ CFU + 100 mg microcrystalline cellulose (per capsule)* 4 wk	Diet not monitored. Participants were required not to supplement with fermented food, probiotics, prebiotics, and antibiotics during the whole experimental process. Training not controlled. Maintain a regular lifestyle, avoiding any strenuous exercise, staying up late, smoking, or consuming alcoholic beverages.	Bacterial taxa Fecal SCFA
Klein et al. (75)	*n* = 26 (13M/13F), age 25 yrs (activity not stated) RXT	Power analysis performed using PASS 6.0 (NCSS Statistical Software, Kaysville, UT, USA). Based on data from the literature and study group to evaluate sample size. It resulted in a power of 90%.	300 g yogurt containing *B. lactis* 420 ×, 3.0 × 10^6^ CFU/g *L. acidophilus* 74-2, 9.3 × 10^8^ CFU/g 300 g daily for 5 wk	Food provided and additional foods aliquoted and analyzed (^NS^between groups). Activity not stated.	Bacterial taxa Fecal SCFA
Lamprecht et al. (76)	*n* = 23 male triathletes, runners and cyclists Age: Int: 37.6 yrs, Pla: 38.2 yrs VO_2max_: Int: 51.2 mL/kg/min Pla: 50.3 mL/kg/min RCT	Sample size calculation based on oxidation markers CP and MDA. Between 7 and 9 subjects estimated per group—depending on parameter, SD and effect size—to reach probability of error (alpha/2) of 5 and 80% power.	Ecologic^®^ Performance: *B. bifidum W23, B lactis W51, E. faecium W54, L. acidophilus W22, L. brevis W63, L. lactis W58*. 2 × 2 g sachets daily for 14 wk, providing (10^10^ CFU/day	Habitual diet, food diary and repeated for 7 days prior to each exercise trial. Habitual training, no exercise 3 days prior to each exercise test.	Intestinal permeability Cytokine responses
Lee et al. (77)	*n* = 16 healthy untrained males Age Int 24.6 ± 2.8, Pla: 25.6 ± 4.1 VO_2max_: Int: 47.3 ± 6.5, Pla: 46.6 ± 8.2 RCT	The required sample sizes for clinical trials based on expected change calculated using Harvard calculator (http://hedwig.mgh.harvard.edu/sample_size/size.html, accessed on 14 December 2020), assuming parallel design with 0.05 significance level, the change SD, power of 0.8, standard deviation of the difference with 3.2.	Synkefir: *L. paracasei DSM 32785 (LPC12), L. rhamnosus DSM 32786 (LRH10), L. helveticus DSM 32787 (LH43), L. fermentum DSM 32784 (LF26), and S. thermophilus DSM 32788 (ST30)* 20 g pouch daily for 28 days	All volunteers were prohibited from taking probiotics, prebiotic fermented products (yogurt or cheese), vitamins, minerals, herbal extracts, dietary supplements for exercise, or antibiotics to avoid unnecessary interference during the experiment. No significant difference on their daily nutritional intake and calories (data not shown). Activity not stated.	Bacterial taxa
Lin et al. (78)	*n* = 21 (14M, 7F) well trained middle and long distance runners. Fitness status not reported. Age: Pla: 21.2 ± 0.4 Int: 21.6 ± 0.7 RCT	Not specified	OLP-01, a human strain probiotic; Bifidobacterium longum subsp. Longum 3 capsules daily (1.5 × 10^10^ CFU/day) for 5 wk	Instructed not to consume nutritional supplements, yogurt, Yakult, other probiotic-related products, or antibiotics during the experiment. The team dietitian specified the diet and provided the same meal to ensure the consistency of the diet (Data not shown). Three weeks of regular training and 2 weeks of de-training. During the experiment, all the subjects cooperated with the team for work and rest (Data not shown).	Bacterial taxa
Pugh et al. (79)	*n* = 24 (20M/4F) marathon runners, age: Int: 34.8 ± 6.9 yrs Pla: 36.1 ± 7.5 yrs VO_2max_: Int: 57.6 ± 8.0 mL/kg/min Pla: 56.4 ± 8.6 mL/kg/min RCT	Not specified	Proven Probiotics Ltd, Port Talbot, Wales: *L. acidophilus* CUL60 *L. acidophilus* CUL21 *B. bifidum* CUL20 *B. animalis* subsp. *Lactis* CUL34 > 25 billion CFU/cap 1 capsule daily for 4 wk	Dietary control not stated outside of acute exercise. Training diary kept during supplementation period (data not presented).	GIS
Sánchez Macarro et al. (80)	*n* = 43 healthy male volunteers who performed aerobic physical exercise. VO2max= 51.1 (8.8)mL/kg/min Age: Int: 25.3 ± 7.2, Pla: 27.1 ± 8.4 RCT	The sample size was calculated for an expected mean difference between groups in serum levels of MDA of 1.34 nmol/mL with SD of 1.6 nmol/L (81), with significance level of 5% and statistical power of 80%, assuming a drop-out rate of 10% since the primary analysis was performed in the PP data set, 20 evaluable participants for each treatment group were required.	Bifidobacterium longum CECT 7347 *Lactobacillus casei* CECT 9104, and *Lactobacillus rhamnosus* CECT 8361 1 capsule daily (10^9^ CFU/day) for 6 wk	No dietary control: During the study period, there were no dietary restrictions, but medications that may affect the microbiome (e.g., antioxidants, statins) were not allowed. Physical activity not controlled	Bacterial taxa
Schreiber et al. (82)	*n* = 27 male cyclists, Age: Int: 25.9 ± 4.6 Pla: 29.5 ± 6.2 VO_2max_ (mL/kg/min) Int: 66.9 ± 6.4 Pla: 63.2 ± 5.0 ^NS^ difference between groups RCT	Not specified	*L. helveticus* Lafti L10, *B. animalis* ssp. lactis Lafti B94 *E. faecium* R0026, *B. longum* R0175 Bacillus subtilis R0179 15 × 10^9^ CFU of a probiotic blend: 1 capsule daily for 90 days	Diet not controlled. Continued with their normal training routine throughout the study duration. “Participants' characteristics analysis revealed difference in training hours during the study period.” Data not shown.	GIS Cytokine responses
Smarkusz-Zarzecka et al. (83)	*n* = 66 (46M/20F) runners, age: Int: F 37.21 ± 8.09 y M 40.85 ± 8.32 y Pla: F 33.33 ± 8.73 y M 38.61 ± 8.84 y VO_2max_ (mL/kg/min) Int: male: 38.22 ± 5.99 Female:34.02 ± 5.30 Pla: male: 42.34 ± 7.06 female:36.98 ±11.34 RCT	Not specified	Sanprobi Barrier, Sanprobi Ltd., Szczecin, Poland: *Bifidobacterium lactis W52, Lactobacillus brevis W63, Lactobacillus casei W56, Lactococcus lactis W19, Lactococcus lactis W58, Lactobacillus acidophilus W37, Bifidobacterium bifidum W23, Lactobacillus salivarius W24*. 2.5 × 10^9^ CFU/g (1 capsule); 2 capsules, twice daily for 3 months.	Diet not controlled. Avoid physical activity for at least 24 h before the test.	Cytokine responses
Son et al. (84)	*n* = 15 bodybuilders (biological sex and fitness status not stated) Age: Int: 26.50 ± 5.01 Pla: 27.14 ± 5.93 RCT	Not specified	*L. acidophilus, L. casei, L. helveticus, Bifidobacterium bifidum* 1 capsule consisting of 10^12^ CFU For 60 days	The subjects were periodically monitored to ensure that nutritional intake was not altered during the supplement intake period; There was no significant difference in the characteristics of the study subjects before and after the probiotic intake period. Data not shown. Physical activity not monitored.	Bacterial taxa Fecal SCFA
Strasser et al. (85)	*n* = 29 (13M/16F) cyclists Age: Int: 25.7 ± 3.5 yrs, Pla: 26.6 ± 3.5 yrs VO_2max_: Int: 55.1 ± 6.4 mL/kg/min Pla: 47.5 ± 7.1 mL/kg/min (*p* < 0.01) W_max_: Int: 325 ± 54.2 W Pla: 274 ± 51.6 W (*p* < 0.05) RCT	Sample size calculation was based on changes in exercise-induced Trp levels (86) from baseline to end of 12-wk intervention. Between 10 and 12 subjects per group estimated, depending on SD and effect size, to reach probability of error (alpha/2) of 5 and 80% power. Allowing for a drop-out rate of 30%, 16 subjects per group were recruited.	Ecologic^®^ Performance: *Bifidobacterium bifidum* W23 *Bifidobacterium lactis* W51 *Enterococcus faecium* W54 *Lactobacillus acidophilus* W22 *Lactobacillus brevis* W63 *Lactococcus lactis* W58 1 × 10^10^ CFU/sachet 1 sachet daily for 12 wk	No alcohol or fermented dairy products. 3 d food record at baseline and 12 wks. ^NS^Between groups. Maintained normal training. Weekly training log. Int: 8.0 ± 2.3 h/wk Pla: 6.6 ± 4.3 h/wk (*p* < 0.001)	GIS
Tavares-Silva et al. (87)	*n* = 14 male runners Age: Pla: 38.28 ± 3.09 Int: 41.57 ± 3.20 VO_2Peak_ (kg/mL/min): Pla: 54.53 ± 6.88 Int: 56.92 ± 8.35 RCT	Not specified	Gelatinous capsules: *Lactobacillus acidophilus-LB-G80, Lactobacillus paracasei-LPc-G110, Lactococcus subp. lactis-LLL-G25, Bifidobacterium animalis subp. lactis-BL-G101, Bifidobacterium bifidum-BB-G90* 5 × 10^9^ CFU 2.0 g/day, 1 capsule/d, 30 days	Dietary questionnaire 2x/wk + once on weekends: Kcal, carbohydrates, protein, lipids ^NS^between groups. Physical activity not reported.	Cytokine responses
Townsend et al. (88)	*n* = 25 male baseball players Age: 20.1 ± 1.5 yrs 1RM Squat (baseline, mean): Int: 116.8 kg, Pla: 133.0 kg Deadlift 1RM (baseline mean): Int: 139.9 kg, Pla: 162.8 kg RCT	Not specified, however study reported as statistically under-powered to detect modest effects in some biomarkers.	*Bacillis subtilis* DE111, 1.24 × 10^9^ CFU/cap 1 capsule daily for 12 wk	3 d food diary on wk 1, 9, 12 ^NS^between groups. 12 wk triphasic, undulating, periodized resistance training program	Intestinal permeability Cytokine responses
Vaisberg et al. (89)	*n* = 42 male runners Age: Int: 39.6 yrs, Pla: 40.1 yrs VO_2max_ (mL/kg/min): Int: 57.64, Pla: 57.86 RCT	Not specified	*Lactobacillus casei Shirota*, 40 × 10^9^ live cells/bottle 1 × 80 ml bottle daily for 30 days	Dietary control not stated. Instructed to maintain usual training exercise schedule—not reported.	Cytokine responses
West et al. (90)	*n* = 88 (62M/35F recruited) cyclists and triathletes Age: Int: M: 35.2 yrs, F: 36.5 yrs, Pla: M: 36.4 yrs, F 35.6 VO_2max_ (ml/kg/min): Int: M: 56.5, F: 53.0 Pla: M: 55.8, F: 51.6 RCT	A sample size of *n* = 80 required for identifying substantial changes in the incidence of illness (91). We assumed a rate of URTI symptoms of 60% in the placebo group, with sufficient power (86% at an alpha-level of 0.05) to detect a 50% reduction in symptoms.	*Lactobacillus fermentum* VRI-003 PCC^®^, 10^9^ CFU/cap 1 capsule daily for 11 wk	4 day food diary. Usual diet, without probiotic foods. Training log kept	Cytokine responses Bacterial taxa GIS
**Synbiotic studies**					
Coman et al. (92)	*n* = 10 (3M/7F), age (range) 20–45 yrs (activity not stated) RCT	Not specified	Synbiotec S.r.l., Camerino, Italy: *L. rhamnosus* IMC 501[R] *L. paracasei* IMC 502[R] plus oat bran fiber 200 ml fermented milk, containing 1 × 10^9^ CFU strain per portion. 200 ml Consumed daily for 4 wk	Not stated	Bacterial taxa GIS
Quero et al. (93)	*n* = 27 (14 sedentary males/13 professional male soccer players 2nd Div B level of the Spanish National League Age: Sedentary: Pla: 24.31 ± 3.94, Int: 23.04 ± 2.09 Athletes: Pla: 21.9 ± 2.77, Int: 20.66 ± 1.39 RCT	Not specified	Gasteel Plus^®^ (Heel España S.A.U laboratories) *B. lactis* CBP-001010, *L. rhamnosus* CNCM I-4036, *B. longum* ES1, Fructooligosaccharides (200 mg) 1.5 mg of zinc, 8.25 μg of selenium, 0.75 μg of vitamin, and maltodextrin as an excipient. 1 stick containing ≥1 × 10^9^ CFU daily for 30 days	Participants were prohibited from consuming probiotics, prebiotics, or fermented products (yogurt or other foods) and any medications that could interfere with the study protocol Subjects were asked to maintain, 2 weeks before and during the study, their regular lifestyle.	Cytokine responses
Roberts et al. (94)	*n* = 20 (18M/2F) long course triathletes Age 35 yrs VO_2max_: Int: 47.6 mL/kg/min Pla: 50.5 ml/kg/min RCT	Power calculation assessment for sample size [G*power3, Dusseldorf (95)]; using α = 0.05; 1 – β = 0.80; based on observed data.	Bio-Acidophilus Forte, Biocare Ltd., Birmingham, UK): *L. acidophilus* CUL-60 (NCIMB 30157), 10^10^ CFU/cap *L. acidophillus* CUL-21 (NCIMB 30156), 10^10^ CFU/cap *B. bifidum* CUL-20 (NCIMB 30172), 9.5^10^ CFU/cap *B. animalis* subspecies *lactis* CUL-34 (NCIMB 30153), 0.5^10^ CFU/cap Fructooligosaccharides, 55.8 mg per cap 1 capsule daily for 90 days	Habitual diet, food diary first and last wk of each month. ^NS^ between groups or over intervention time period. Prescribed triathlon training program, individualized. ^NS^Between groups for training load throughout intervention period.	Intestinal permeability Endotoxin responses GIS
Valle et al. (96)	*n* = 65 (39M/26F) Military recruits Age: Int: 19·69 ± 1·25 Pla: 19·5 ± 1·22 RCT	Sample calculation in G * Power 3.1.9.2 software was based on the following data: 5% sample error, 95% CI and 0.72 effect size considering pre and post-intervention IgA values. The effect size was estimated based on the study by Olivares et al. (97).	*60 g ice cream containing:* *Lactobacillus acidophilus LA-5, 10.3 log CFU; Bifidobacterium animalis BB^−1^2, 11.0 log CFU 2.3 g of inulin daily for 30 days*	We recommended participants not to consume any foods containing prebiotics and probiotics (e.g., probiotic yogurts, fermented milk) 15 d before the beginning of the research period, particularly over the weekend, when they are released to go home. This consumption was controlled during the week as all food was provided (data not shown). Not stated during the supplementation period however the participants were undergoing training in a military boarding school.	Bacterial taxa Fecal SCFA GIS
West et al. (98)	*N* = 22 male cyclists Age: Syn: 34.4 ± 3.5 yrs, Pre: 31.4 ± 4.9 yrs VO_2max_: Syn: 57.9 ± 7.3 ml/kg/min Pre: 56.4 ± 4.9 ml/kg/min RCT	Sample size was determined based on variance analysis (standard deviations) from previous studies on the parameters of interest. To demonstrate a difference of 0.20 of the pooled between-subject standard deviation in the salivary immune parameters, which have previously shown the largest variance, a total of nine subjects per group were required to give 80% power at an α level of 0.05.	Synbiotic capsules (Biosource™ Gut Balance, Probiotech Pharma): *L. paracasei* subs Paracasei (*L. casei* 431^®^), 4.6 × 10^8^ per cap *Bifidobacterium animalis* ssp. lactis (BB-12^®^), 6 × 10^8^ per cap *L. acidophilus* LA-5, 4.6 × 10^8^ per cap *L. rhamnosus* GG, 4.6 × 10^8^ per cap Raftiline, 90 mg per cap Raftilose GR, 10 mg per cap Prebiotic capsules: Acacia powder, 116 mg per cap 3 capsules daily for 3 wk	14 days run-in, no yogurt or products influencing microbiome. Training log kept: Training load/wk: (duration × intensity) Syn: 21.3 ± 18.5 Pro: 21.4 ± 16.8^NS^	Cytokine responses Intestinal permeability Bacterial taxa Fecal SCFA GIS

RCT, Randomized control trial; RXT, randomized crossover trial; SCFA, short chain fatty acids; wk, weeks; NS, not significant.

### Intestinal epithelial injury at rest

No studies were identified as assessing intestinal epithelial injury at rest, before or after a period of pre-, pro-, or syn-biotic supplementation.

### Intestinal permeability at rest

*N* = 6 studies assessed markers of intestinal permeability at rest, before and after the supplementation period (Tables 3, 4). Reported markers included urinary lactulose:mannitol ratio (62, 94, 98), fecal (62, 63, 76) and serum zonulin (62, 88). One study observed a reduction in both urinary lactulose:mannitol ratio and serum zonulin, pre- to post-supplement period with inulin-enriched pasta ingestion, and no change was observed in the placebo trial (62). *N* = 1 study reported a 20% reduction in fecal zonulin following 14 weeks supplementation with a multi-strain probiotic (*B. bifidum* W23, *B. lactis* W51, *E. faecium* W54, *L. acidophilus* W22, *L. brevis* W63, and *L. lactis* W58), with the post-supplementation intervention value significantly lower than placebo that remained unchanged from baseline (76). No statistically significant differences were observed for other outcomes or interventions.

**Table 4:** Systematic review study outcomes of included studies that attempted to determine the impact of prebiotic, probiotic, and synbiotic supplementation on gastrointestinal outcomes at rest.

**References**	***N* and study design**	**Supplement/** **comparator**	**Intervention ingredient/s and supplement duration**	**Outcome measure/s (Δ in mean/median from pre- to post-supplementation period unless otherwise indicated)**
**Intestinal permeability**				
Russo et al. (62)	*N* = 20 Study design: RXT	Prebiotic vs. placebo	Inulin-enriched pasta, 5 wk	Urinary lactulose/mannitol ratio: Int ↓ 0.02, Pla ↔ 0.00 (*p* < 0.05). Serum zonulin: Int ↓ 1.61 ng/ml, Pla ↑ 0.35 ng/ml (*p* < 0.05). *Fecal zonulin:* Int ↑ 0.01 μg/g, Pla ↔ 0.00 μg/g^NS^
Axelrod et al. (63)	*n* = 7 RXT	Probiotic vs. placebo	*L. salivarius* UCC118, 4 wk	Fecal zonulin: Int ↓ 0.18 mg/dL, Pla ↓ 0.2 mg/dL ^NS^
Lamprecht et al. (76)	*n* = 23 RCT	Probiotic vs. placebo	*B. bifidum W23, B lactis W51, E. faecium W54, L. acidophilus W22, L. brevis W63, L. lactis W58*, 14 wk	Fecal zonulin: Int ↓ 8.8 ng/ml, Pla ↑ 1.6 ng/ml (*p* = 0.019)
Townsend et al. (88)	*n* = 25 RCT	Probiotic vs. placebo	*Bacillis subtilis* DE111, 12 wk	Serum zonulin: Int ↑ 0.2 ng/ml, Pla ↑ 0.2 ng/ml^NS^
Roberts et al. (94)	*n* = 20 RCT	Synbiotic vs. prebiotic	*L. acidophilus* CUL-60 (NCIMB 30157), *L. acidophillus* CUL-21 (NCIMB 30156), *B. bifidum* CUL-20 (NCIMB 30172), *B. animalis* subspecies *lactis* CUL-34 (NCIMB 30153, Fructooligosaccharides, 12 wk	Urinary lactulose/mannitol ratio: Int ↑ 0.011, Pla ↑ 0.029^NS^
West et al. (98)	*n* = 22 RCT	Synbiotic vs. prebiotic	*L. paracasei* subs Paracasei (*L. casei* 431^®^), *B. animalis* ssp. lactis (BB-12^®^), *L. acidophilus* LA-5, *L. rhamnosus* GG, Raftiline, Raftilose GR, 3 wk	Lactulose/mannitol ratio: ^NS^between groups (data not reported)
**Endotoxin responses**				
Burton et al. (66)	*n* = 13 RXT	Probiotic vs. placebo	*S. thermophilus, L. delbrueckii* spp. *Bulgaricus, L. rhamnosus GG*, 2 wk	LPS: Int ↓ 0.3 pg/ml, Pla ↓ 0.05 pg/ml^NS^
Gill et al. (68)	*n* = 8 RXT	Probiotic vs. placebo	*L. casei*, 1.0 × 10^11^ cells/bottle Commercial supplement, taken twice daily for 7 days	Gram negative endotoxin: Int ↑ 0.1 EU/ml, Pla ↑ 0.3 EU/ml^NS^
Carbuhn et al. (67)	*n* = 17 RCT	Probiotic vs. placebo	*B. longum* 35624, 6 wk	LPS: ^NS^between groups (data not reported) LBP: ^NS^between groups (data not reported)
Roberts et al. (94)	*n* = 20 RCT	Synbiotic vs. placebo	*L. acidophilus* CUL-60 (NCIMB 30157), *L. acidophillus* CUL-21 (NCIMB 30156), *B. bifidum* CUL-20, *B. animalis* subspecies *lactis* CUL-34 (NCIMB 30153), Fructooligosaccharides, 12 wk	Endotoxin units: Int ↓ 2.30 pg/ml, Pla ↓ 0.84 pg/ml ^NS^ IgG endotoxin antibodies (anti-LPS): Int ↑ 42 MU/ml, Pla ↓ 42 MU/ml^NS^
**Cytokine responses**				
Axelrod et al. (63)	*n* = 7 RXT	Probiotic vs. placebo	*L. salivarius* UCC118, 4 wk	IL-6: (ΔΔ pre to post-exercise, pre to post-intervention) Int ↑ 0.5 pg./ml, Pla: ↑ 1.4pg/ml^NS^
Batatinha et al. (65)	*n* = 27	Probiotic vs. placebo	*B. animalis. Lactis 10 × 10^9^; L. Acidophilus 10 × 10^9^* 1 sachet daily for 30 days	IL-10: (baseline to pre-ex Δ) Int: ↓ 5.5 ng/ml, Pla: ↓ 3.2 ng/ml^NS^ IL-4: Int: ↓ 3.0 ng/ml, Pla: ↓ 0.9 ng/ml^NS^ IL-6: Int: ↔ 0 ng/ml, Pla: ↓ 2.5 ng/ml^NS^ IL-2: Int: ↓ 0.4 ng/ml, Pla: ↓ 2.6 ng/ml^NS^ IL-15: Int: ↓ 0.4 ng/ml, Pla: ↓ 0.6 ng/ml^NS^ IL-8 (ng/ml): Int: ↑ 0.4 ng/ml, Pla: ↓ 3.8 ng/ml^NS^ IL-1β: Int: ↓ 0.7 ng/ml, Pla: ↓ 0.8 ng/ml^NS^ TNF-α: Int: ↓ 2.2 ng/ml, Pla: ↓ 3.7 ng/ml^NS^ IFN-γ : Int: ↓ 2.6 ng/ml, Pla: ↓ 9.0 ng/ml^NS^
Burton et al. (66)	*n* = 13 RXT	Probiotic vs. placebo	*S. thermophilus, L. delbrueckii* spp. *Bulgaricus, L. rhamnosus GG*, 2 wk	TNF-α: Int ↑ 0.75 pg/ml, Pla ↑ 0.95 pg/ml^NS^ IL-6: Int ↓ 0.45 pg/ml, Pla ↑ 0.65 pg/ml^NS^ CCL2: Int ↑ 1.8 pg/ml, Pla ↑ 12.55 pg/ml (*p* = 0.01) CCL5: Int ↓ 12.75 pg/ml, Pla ↓ 7.6 pg/ml^NS^
Carbuhn et al. (67)	*n* = 17 RCT	Probiotic vs. placebo	*B. longum* 35624, 6 wk	IL-1ra: Int ↓ 107 pg/ml, Pla ↓ 37 pg/ml^NS^ IFN-γ, IL-1B, IL-2, IL-4, IL-5, IL-6, IL-10, IL-13, IL-17, IL-17F, and IL-22, TNF-α_ were below detectable levels in assay.
Gill et al. (68)	*n* = 8 RXT	Probiotic vs. placebo	*L. casei*, 1 wk	IL-6: Int ↑ 0.1 pg/ml, Pla ↑ 0.4 pg/ml^NS^ IL-1β: Int ↓ 0.05 pg/ml, Pla ↓ 0.02 pg/ml^NS^ TNF-α: Int ↓ 0.1 pg/ml, Pla ↑ 0.2 pg/ml^NS^ IFN-γ: Int ↑ 0.1 pg/ml, Pla ↑ 0.7 pg/ml^NS^ IL-10: Int ↑ 1.2 pg/ml, Pla ↑ 4.8 pg/ml^NS^ IL-8: Int ↔ 0.0 pg/ml, Pla ↑ 0.3 pg/ml^NS^
Hoffman et al. (73)	*n* = 15 RCT	Probiotic vs. placebo	*Inactivated Bacillus coagulans; 2 wk*	IFN-γ : Int: ↓ 0.2 pg/ml, Pla: ↓ 3.6 pg/ml^NS^ IL-10 : Int: ↑ 0.4 pg/ml, Pla: ↓ 1.4 pg/ml^NS^ IL1-B: Int: ↑ 0.3 pg/ml, Pla: ↑ 2.8 pg/ml^NS^ IL-2: Int: ↓ 0.3 pg/ml, Pla: ↓ 0.3 pg/ml^NS^ IL-6: Int: ↓ 0.2 pg/ml, Pla: ↓ 1.0 pg/ml^NS^ IL-8: Int: ↓ 2.4 pg/ml, Pla: ↓ 3.6 pg/ml^NS^ TNF-α: Int: ↓ 1.7 pg/ml, Pla: ↓ 4.5 pg/ml^NS^
Lamprecht et al. (76)	*n* = 23 RCT	Probiotic vs. placebo	*B. bifidum W23, B lactis W51, E. faecium W54, L. acidophilus W22, L. brevis W63, L. lactis W58*, 14 wk	TNF-α: Int ↓ 17.1 pg/ml, Pla ↑ 4.7 pg/ml^NS^ IL-6: Int ↓ 1.0 pg/ml, Pla ↑ 0.1 pg/ml^NS^
Schreiber et al. (82)	*n* = 27 RCT	Probiotic vs. placebo	*L. helveticus* Lafti L10, *B. animalis* ssp. lactis Lafti B94 *E. faecium* R0026, *B. longum* R0175 *Bacillus subtilis* R0179, 90 days	ANCOVA, (Δ) changes from baseline, adj. for training loads. IL-6 adj: Int: 0.11 ± 0.64, Pla: −0.25 ± 0.6^NS^ TNF-α adj: Int: −0.02 ± 0.23, Pla: 0.06 ± 0.21^NS^ CRP adj: Int: 443.82 ± 238.73, Pla: 231.55 ± 381.28^NS^
Smarkusz-Zarzecka et al. (83)	*n* = 66 RCT	Probiotic vs. placebo	*B. lactis* W52, *L. brevis* W63, *L. casei* W56, *Lactococcus lactis* W19, *Lactococcus lactis* W58, *L. acidophilus* W37, *B. bifidum* W23, *L. salivarius* W24. 3 months	CRP: Male: Δ: Int: ↓0.12 mg/L, Pla: ↓ 0.31 mg/L^NS^ Female Δ: Int: ↓1.3 mg/L, Pla: ↓ 0.6 mg/L^NS^ TNF-α: Male: Δ: Int: ↓ 1.62 mg/L, Pla: ↓ 0.88 mg/L^NS^ Female: Δ: Int: ↓ 1.43 mg/L, Pla: ↓1.72 mg/L^NS^
Tavares-Silva et al. (87)	*n* = 14 RCT	Probiotic vs. placebo	Gelatinous capsules: *Lactobacillus acidophilus-LB-G80, Lactobacillus paracasei-LPc-G110, Lactococcus* subp. *lactis-LLL-G25, Bifidobacterium animalis* subp. *lactis-BL-G101, Bifidobacterium bifidum-BB-G90;* 30 days	IL-2 (Baseline to 24 h before marathon) Int: ↓ 0.37 pg/ml (*p* < 0.04), Pla: ↓ 0.2 pg/ml^NS^ IL-4 (Baseline to 24 h before marathon) Int: ↓ 0.73 pg/ml^NS^, Pla: ↓ 0.89 pg/ml (*p* < 0.04) IL-10 (Baseline to 24 h before marathon) Int: ↓ 0.97 pg/ml (*p* < 0.001), Pla: ↓ 0.05 pg/ml^NS^ TNF-α (Baseline to 24 h before marathon) Int: ↑ 0.09 pg/ml^NS^, Pla: ↓0.05 pg/ml^NS^
Townsend et al. (88)	*n* = 25 RCT	Probiotic vs. placebo	*Bacillis subtilis* DE111, 12 wk	TNF-α: Int: Δ: ↓ 0.25 pg/ml, Pla: Δ: ↑ 0.36 pg/ml Int ↓ Pla, *p* = 0.024 IL-10: Int: Δ: ↑ 0.1 pg/ml, Pla Δ: ↑ 0.15 pg/ml^NS^
Vaisberg et al. (89)	*n* = 42 RCT	Probiotic vs. placebo	*L. casei Shirota*, 30 days	IL-1β: Int ↑ 22.7 pg/ml, Pla ↑ 20.9 pg/ml^NS^ IL-1ra: Int ↑ 16.3 pg/ml, Pla ↑ 10.5 pg/ml^NS^ IL-4: Int ↑ 9.4 pg/ml, Pla ↑ 11.3 pg/ml^NS^ IL-5: Int ↑ 7.2 pg/ml, Pla ↑ 6.7 pg/ml^NS^ IL-6: Int ↑ 4.4 pg/ml, Pla ↑ 0.9 pg/ml^NS^ IL-10: Int ↑ 5.7 pg/ml, Pla ↑ 2.6 pg/ml^NS^ IL-12p70: Int ↑ 6.9 pg/ml, Pla ↑ 3.6 pg/ml^NS^ IL-13: Int ↑ 7.3 pg/ml, Pla ↑ 6.9 pg/ml^NS^ TNF-α: Int ↑6.6 pg/ml, Pla ↑ 22.2 pg/mL^NS^
Quero et al. (93)	*n* = 27 RCT	Synbiotic vs. placebo	*B. lactis* CBP-001010, *L. rhamnosus* CNCM I-4036, *B. longum* ES1, Fructooligosaccharides, 30 days	IL-1β: Sedentary: Int: ↑ 0.3 pg/mL (*p* < 0.01), Pla: ↑ 0.1 pg/mL^NS^ Athletes: Int: ↓ 0.2 pg/mL^NS^, Pla: ↓ 0.2 pg/mL^NS^ IL-10 Sedentary: Int: ↓ 0.3 pg/mL (*p* < 0.01), Pla: ↓ 0.4 pg/mL (*p* < 0.05) Athletes: Int: ↑ 0.05 pg/mL^NS^, Pla: ↓ 0.05 pg/mL^NS^
West et al. (98)	*n* = 22 RCT	Synbiotic vs. prebiotic	*L. paracasei* subs Paracasei (*L. casei* 431^®^), *B. animalis* ssp. lactis (BB-12^®^), *L. acidophilus* LA-5, *L. rhamnosus* GG, Raftiline, Raftilose GR, 3 wk	IL-16: 50% greater increase in Pre vs. Syn (*p* = 0.02) IL-18: ^NS^between pre and syn, no additional data shown IL-12 and IFN-γ: Undetectable in assay
**Gastrointestinal function**				
Damen et al. (55)	*n* = 27 RXT	Prebiotic vs. placebo	Arabinoxylan oligosaccharides (AXOS), 3 wk	Defecation frequency: Int ↓ 0.1/day, Pla ↑ 0.1/day (*p* < 0.05) Bristol stool form scale: Int ↓ 0.1/day, Pla ↑ 0.2/day^NS^
Kleessen et al. (59)	*n* = 45 RCT	Prebiotic vs. prebiotic vs. placebo	Chicory inulin (CH) or Jerusalem artichoke inulin (JA), 3 wk	Defecation frequency: CH ↑ 3/wk (*p* < 0.05), JA ↑ 2/wk (*p* < 0.05), Pla ↑ 2/wk (*p* < 0.05) Stool consistency (1–4 scale, hard to soft): CH: ↑ 2 (*p* < 0.05), JA: ↑ 3 (*p* < 0.05), Pla: ↑ 1^NS^
Russo et al. (61)	*n* = 20 RXT	Prebiotic vs. placebo	Inulin-enriched pasta, 5 wk	Ultrasound full gastric emptying time: Int ↑ 30 min (*p* < 0.05), Pla ↔ 0 min^NS^ Electrogastrography (% normal slow waves): Pre-Prandial: Int ↑ 12.5%, Pla ↑ 6.5% (*p* = 0.05) Post-prandial: Int ↑ 5.6%, Pla ↑ 2.0% (*p* = 0.03)
Russo et al. (45)	*n* = 20 RXT	Prebiotic vs. placebo	Inulin-enriched pasta, 5 wk	Ultrasound half gastric emptying time: Int ↑ 8.3 min (*p* < 0.05), Pla ↑ 1.4 min^NS^
Reimer et al. (60)	*N* = 48 RXT	Prebiotic vs. Prebiotic vs. placebo	Chicory inulin type fructans (ITF), 4 wk	Stools/d (Δ c/f baseline): Int 1: −0.1 ± 0.2, Con 1: 0.3 ± 0.2^NS^ Int 2: −0.1 ± 0.2, Con 2: 0 ± 0.1^NS^ Bristol Stool Rating [(1–7) Δ c/f baseline]: Int 1: −0.1 ± 0.3, Con 1: −0.4 ± 0.3^NS^ Int 2: −0.1 ± 0.3, Con 2: 0 ± 0.3^NS^
**Bacterial taxa**				
Damen et al. (55)	*n* = 27 RXT	Prebiotic vs. placebo	Arabinoxylan oligosaccharides (AXOS), 3 wk	FISH analysis to count number of different bacterial groups. Total bacteria cell counts were determined by 4′-6-diamidino-2-phenylindole. ^NS^Changes in abundance or diversity between groups or pre-post supplementation in the same group
Finegold et al. (56)	*n* = 32 RCT	Prebiotic vs. Prebiotic vs. placebo	Xylooligosaccharides (XOS), 8 wk	Bacterial diversity (Operational Taxonotic Units, species level and Shannon index): ^NS^in α-diversity (OTU) or Shannon index. 16S rRNA gene sequencing/log_10_ scale of bacterial counts (CFU/g) ↑*Bifidobacterium* count in high dose XOS only (*p* < 0.05) ↑*Bacteroides fragilis* in high dose XOS only (*p* < 0.05) ↑ total anaerobes count in high dose XOS only (*p* < 0.05) ^NS^For total aerobes, *Lactobacillus, Enterobacteriaceae*, and *Clostridium* counts cf. baseline in all groups ↓*Enterobacteriaceae* count *cf* placebo after washout (*p* < 0.05)
François et al. (57)	*n* = 63 RXT	Prebiotic vs. Prebiotic vs. placebo	Arabinoxylan oligosaccharides (AXOS), 3 wk	FISH analysis to count number of different bacterial groups. Total bacteria cell counts were determined by 4'-6-diamidino-2-phenylindole. Percentage of bifidobacterial calculated as the ratio of the absolute amounts of bifidobacteria to the total bacterial cell count. *Bifidobacteria* (*log_10_ counts/g dry weight feces):* High: 9.3, Low: 9.0, Pla: 8.9 High vs. Low *p* < 0.05 High vs. Pla *p* < 0.001^NS^ for *Lactobacilli, Faecalibacterium prausnitzii, Clostridium histolyticum–lituseburense* or *Roseburia–Eubacterium rectale*
Kleessen et al. (59)	*n* = 45 RCT	Prebiotic vs. prebiotic vs. placebo	Chicory inulin (CH) or Jerusalem artichoke inulin (JA), 3 wk	Bacterial counts were assessed by fluorescent *in situ* hybridization or colony forming units, as assessed by conventional culture methods.
				*All data expressed in log^10^ counts/g wet weight feces* Total bacteria: CH: ↑ 0.1, JA: ↔0, Pla: ↔0, ^NS^Clostridium coccoides/Eubacterium rectale cluster : CH: ↓ 0.6 (*p* < 0.05), JA: ↓ 0.6 (*p* < 0.05), Pla: ↓0.3, ^NS^Bacteroides/Prevotella: CH: ↓ 0.4 (*p* < 0.05), JA: ↓ 0.6 (*p* < 0.05), Pla: ↑ 0.1 CH and JA both > Pla (*p* < 0.05) Faecalibacterium prausnitzii: CH: ↓ 0.2, JA: ↓ 0.2, Pla: ↓ 0.1, ^NS^Bifidobacterium: CH: ↑ 1.2, JA: ↑ 1.2, Pla: ↑ 0.3 CH and JA both > Pla (*p* < 0.05) Atopobium group: CH:↔ 0, JA: ↓ 0.2, Pla: ↔ 0, ^NS^Lactobacillus: CH: ↓ 0.9, JA: ↓ 0.5, Pla: ↓ 0.7, ^NS^Enterococcus: CH: ↓ 0.9, JA: ↓ 0.4, Pla: ↑ 0.4, ^NS^Enterobacteriaceae: CH: ↓ 0.4, JA: ↓ 0.9, Pla: ↓ 0.7^NS^
Reimer et al. (60)	*n* = 48 RXT	Prebiotic vs. Prebiotic vs. placebo	Chicory inulin type fructans (ITF), 4 wk	Results are expressed as relative abundance (%) of *Bifidobacterium* per total bacteria (*Bifidobacterium* 16S rRNA gene copies × 100/total 16S rRNA gene copies). Bacterial diversity ^NS^in α-diversity. Community Structure ^NS^ in β-diversity Microbial abundance (phylum) *(Con = > Int 1/Int 1 = > Con)* Actinobacteria: Con: 6.02 ± 5.26 Int 1: 15.23 ± 12.37, ↑ 153% *p* < 0.01(adj) Int: 11.70 ± 8.65 Con: 6.36 ± 3.95 ↓ 83% *p < * 0.01(< adj) Firmicutes Con: 85.91 ± 9.02 Int 1: 78.72 ± 10.96, ↓ 8% *p* < 0.01(adj) Int: 82.52 ± 9.58 Con: 88.13 ± 4.37 ↑ 6% *p* < 0.01(adj) Bacteroidetes Con: 6.51 ± 7.96 Int 1: 3.48 ± 4.26, ↓ 46% *p* = 0.05(adj) *(Con = > Int 2/Int 2 = > Con)* Actinobacteria Con: 8.07 ± 7.38 Int 2: 13.19 ± 12.37 0.01 ↑ 63% ^NS^Proteobacteria Con: 0.79 ± 1.29 Int 2: 0.38 ± 0.39 0.04 ↓ 51%^NS^ *Family (Con ≥ Int 1/Int 1 ≥ Con)* Bifidobacteriaceae Con: 2.52 ± 2.90 Int 1: 10.28 ± 9.09, *p* < 0.01(adj) ↑ 308% *p* < 0.01(adj) 7.57 ± 8.08 2.63 ± 1.88 ↓ 65% *p* < 0.01(adj)
				Actinomycetaceae Con: 0.06 ± 0.09 Int 1: 0.24 ± 0.29 ↑ 300% *p* < 0.01(adj) Int 1: 0.23 ± 0.24 Con: 0.09 ± 0.08 ↑ 60% *p* < 0.01(adj) Microbacteriaceae Con: 0.003 ± 0.01 Int 1: 0.01 ± 0.02 ↑ 233% *p* < 0.01(adj) Int 1: 0.01 ± 0.02 0.0006 ± 0.003 ↓ 94% *p* < 0.05(adj) Cellulomonadaceae Con: 0.0003 ± 0.002 Int 1: 0.01 ± 0.01 ↑ 3,233% *p* < 0.01(adj) Micrococcaceae Con: 0.06 ± 0.10 Int 1: 0.19 ± 0.33 ↑ 216% *p* < 0.01(adj) Brevibacteriaceae Con: 0.01 ± 0.03 Int 1: 0.03 ± 0.05 ↑ 200% *p* < 0.01(adj) *Family (Con ≥ Int 2/Int 2 ≥ Con)* Micrococcaceae Con: 1.18 ± 1.72 Int 2: 2.37 ± 2.67 ↑ 101%^NS^
				Vibrionaceae Con: 0.21 ± 0.44 Int 2: 0.05 ± 0.08 ↓ 76%^NS^ Bifidobacteriaceae Con: 1.17 ± 1.91 Int 2: 2.39 ± 3.63 ↑ 104%^NS^ Enterobacteriaceae Con: 0.42 ± 0.93 Int 2: 0.10 ± 0.17 ↓ 76^NS^ Actinomycetaceae Con: 0.44 ± 0.77 Int 2: 0.88 ± 1.44 ↑ 100%^NS^ *Genus (Con ≥ Int 1/Int 1 ≥ Con)* Bifidobacterium Con: 5.30 ± 5.87 Int 1: 18.73 ± 14.99, ↑ 253% p < 0.01(adj) Int 1: 11.91 ± 12.02 Con: 4.63 ± 3.42 ↓ 61% *p* < 0.01(adj) Actinomyces Con: 0.13 ± 0.18 Int 1: 0.45 ± 0.49, ↑ 246% *p* < 0.01(adj) Int 1: 0.37 ± 0.38 Con: 0.16 ± 0.15 ↓ 56% *p* < 0.02(adj) Cellulomonas Con: 0.0007 ± 0.01 Int 1: 0.01 ± 0.03 ↑ 1,328% *p* < 0.02(adj) Nesterenkonia Con: 0.12 ± 0.21 Int 1: 0.35 ± 0.54 ↑ 191% *p* < 0.03(adj) Lachnospira Con: 2.20 ± 2.70 Int 1: 0.93 ± 1.36 ↓ 57% *p* < 0.04(adj) Oscillospira Con: 1.11 ± 1.01 Int 1: 0.65 ± 0.54 ↓ 41% *p* < 0.04(adj) Brevibacterium Con: 0.03 ± 0.05 Int 1: 0.06 ± 0.08 ↑ 100% *p* < 0.04(adj) *Genus (Con = > Int 2)* Nesterenkonia Con: 2.46 ± 3.32 Int 2: 4.86 ± 4.75 ↑ 97% ^NS^ Vibrio Con: 0.50 ± 1.12 Int 2: 0.10 ± 0.16 ↓ 80% ^NS^ Bifidobacterium Con: 2.47 ± 3.83 Int 2: 4.62 ± 6.13 ↑ 87%^NS^ Actinomyces Con: 0.91 ± 1.53 Int 2: 1.67 ± 2.46 ↑ 83%^NS^
Axelrod et al. (63)	*n* = 7 RXT	Probiotic vs. placebo	*L. salivarius* UCC118, 4 wk	DNA extraction by shotgun metagenomic sequencing. Shannon and Simpson index ^NS^in α-diversity or richness. *Probiotic data only, no placebo data available* Phyla: *Verrucomicrobia* ↓ 0.144% (*q* = 0.001) Genus: *Prosthecobacter* ↓ 0.141% (*q* = 0.004) Species: *fusiformis ↓* 0.051% (*q* = 0.006)
Burton et al. (66)	*n* = 13 RXT	Probiotic vs. placebo	*S. thermophilus, L. delbrueckii* spp. *Bulgaricus, L. rhamnosus* GG, 2 wk	16S rRNA gene sequencing *Relative abundance compared to baseline* *S. salivarius* spp. *thermophilus*: Int ↑ 0.10%, Pla ↔ 0.0% (*p* < 0.05) *L. delbrueckii* spp. *Bulgaricus*: Int ↑ 0.02%, Pla ↔ 0.0% (*p* < 0.05) *L. rhamnosus* GG: Int ↔ 0.0%, Pla ↔ 0.0% ^NS^ *Bilophila wadsworthia*: Int ↓ 0.07%, Pla ↓ 0.27% ^NS^ *B. kashiwanohense*/*B. pseudocatenulatum*: Int ↓ 0.05%, Pla ↑ 0.05% (*p* < 0.05)
Huang et al. (74)	*n* = 20 male RCT	Probiotic vs. placebo	*L. plantarum PS128, 4 wk*	16S rRNA gene sequencing **No baseline data reported**. % Relative abundance (Phyla) *Int: Fermicutes 46.6%, Bacteriodetes 47.0%, Proteobacteria 3.8%, Actinobacteria 2.1%, Fusobacteria 0.3%* *Pla: Fermicutes 50.3%, Bacteriodetes 41.6%, Proteobacteria 4.9%, Actinobacteria 1.0%, Fusobacteria 1.5%* ^NS^ between groups. Relative abundance (Genus) *Anaerotruncus (× 10^−4^) Int 0, Pla 1.0; Caproiciproducens (× 10^−4^) Int 0.1, Pla 1.0; Coprobacillus (× 10^−5^) Int 0, Pla 3.3; Desulfovibrio (× 10^−5^), Int 0, Pla 5.9; Dielma (× 10^−5^), Int 0, Pla 2.6; Family_XIII_UCG_001 (× 10^−5^), Int 0.9, Pla 9.2; Holdemania (× 10^−5^), Int 0.6, Pla 7.2; Oxalobacter (× 10^−5^), Int 0, Pla 6.1; **Int** **<** **Pla (p** **<** **0.05)*** *Akkermansia (× 10^−3^), Int 5.0, Pla 1.3; Bifidobacterium (× 10^−2^), Int 1.5, Pla 0.8; Butyricimonas (× 10^−3^), Int 4.7, Pla 2.3; Lactobacillus (× 10^−3^), Int 1.7, Pla 0.7; **Int** **>** **Pla (p** **<** **0.05)***
Klein et al. (75)	*n* = 26 RXT	Probiotic vs. placebo	*B. lactis* 420x, *L. acidophilus* 74-2, 5 wk	Preparation of fecal samples by FISH analysis. Relative abundance compared to baseline *B. lactis*: Int ↑ 1.43%, Pla ↑ 0.39% (*p* < 0.05) *L. acidophilus*: Int ↑ 0.18%, Pla ↑ 0.02% (*p* < 0.05)
Lee et al. (77)	*n* = 16 RCT	Probiotic vs. placebo	*L. paracasei DSM 32785 (LPC12), L. rhamnosus DSM 32786 (LRH10), L. helveticus DSM 32787 (LH43), L. fermentum DSM 32784 (LF26), and S. thermophilus DSM 32788 (ST30)* *28 days*	qPCR method was used for the identification and quantification of gut microbiota. *Pre-post Δ in Log10 cells/g* *Lactobacillus:* *Int: ↑ 0.2, Pla: ↑ 0.5, ^*NS*^* *Bifidobacterium:* *Int: ↓ 0.2, Pla: ↑ 0.3 “Decreased in intervention group” (p < 0.05)* *Clostridium: Int: ↓ 1.0, Pla:↓ 1.0 ^*NS*^* *Bacteroides: Int: ↓ 0.3, Pla: ↑ 0.1 ^*NS*^*
Lin et al. (78)	*n* = 21	Probiotic vs. placebo	*Bifidobacterium longum* subsp. *Longum, 5 wk*	16S rRNA gene sequencing *Phylum:* *Int: Actinobacteria and Firmicutes greater abundance post-supplementation, compared with Pla. (p-value not shown)*. *Proteobacteria reduced abundance post-supplementation, compared with Pla. (p-value not shown)*. *Genus:* *Int: ↑ Bifidobacterium compared with Pla (p = 0.0027). 9-fold ↑ in Lactobacillus count*. *Species:* *Bifidobacterium longum subsp. longum relative abundance* *Int: 0.95%; ↑ 8.63-fold (p = 0.0178)*. *Pla: 0.11%* *^*NS*^ in amounts of common strains*
Sánchez Macarro et al. (80)	*n* = 43 RCT		*Bifidobacterium longum CECT 7347 Lactobacillus casei CECT 9104, and Lactobacillus rhamnosus CECT 8361 6 wk*	16S rRNA gene sequencing Bacterial diversity *Richness:* Int:↔ 0, Pla: ↓6 ^NS^ *Simpson index:* Int: ↔ 0, Pla: ↑0.03, ^NS^ *Shannon index:* Int: ↑0.01, Pla: ↑0.15 ^NS^ *Family (log relative counts)* *Rhodospirillaceae:* *Int < Pla, log2 fold = 2.71, p = 0.019 (adj)* *Streptococcaceae:* *Int < Pla, log2 fold = 2.20, p = 0.019(adj)* *Genera (log relative counts)* *Rhodospirillum:* *Pla > Int, p = 0.007(adj)* *Streptococcus:* *Pla > Int, p = 0.007(adj)* *Within group differences noted in genera*.
Son et al. (84)	*n* = 15 RCT	Probiotic vs. placebo	*L. acidophilus, L. casei, L. helveticus, B. bifidum, 60 days*	16S rRNA gene sequencing Shannon and Simpson index ^NS^ in α-diversity, pre and post *Species:* ^NS^ changes in the abundance of the four microorganisms present (three Lactobacilli and one Bifidobacterium).
West et al. (90)	*n* = 88 RCT	Probiotic vs. placebo	*Lactobacillus fermentum* VRI-003 PCC^®^, 11 wk	Microbiome Diversity (16SrRNA) ^NS^ changes in bacterial diversity (data not shown) *All data reported as raw bacterial counts—no statistical testing of between group changes*. Total bacteria: Males: Int ↓ 0.5 × 10^10^, Pla Pre: ↓ 0.5 × 10^10^ Females: Int ↑ 0.7 × 10^10^, Pla ↓ 1.0 × 10^10^ *C. coccoides*: Males: Int ↓ 2.3 × 10^8^, Pla ↓ 3.4 × 10^8^ Females: Int ↔ 0, Pla ↓ 1.54 × 10^9^ *E. coli*: Males: Int ↑ 6.4 × 10^5^, Pla ↑ 6.8 × 10^5^ Females: Int ↑ 1.36 × 10^7^, Pla ↑ 4.3 × 10^4^ *Bifibacteria*: Males: Int ↓ 0.3 × 10^7^, Pla ↓ 5.6 × 10^6^ Females: Int ↑ 0.7 × 10^6^, Pla ↓ 6.1 × 10^6^ *Bacteroides*: Males: Int ↑ 0.6 × 10^6^, Pla ↑ 1.6 × 10^6^ Females: Int ↑ 1.3 × 10^6^, Pla ↓ 4.4 × 10^7^ *Lactobacillus*: Males: Int ↑ 5.8 × 10^4^, Pla ↓ 2.8 × 10^6^ Females: Int ↑ 7.0 × 10^4^, Pla ↑ 6.9 × 10^4^
Coman et al. (92)	*n* = 10 RCT	Synbiotic vs. placebo	*L. rhamnosus* IMC 501[R], *L. paracasei* IMC 502[R], plus oat bran fiber, 4 wk	qPCR procedure for quantification of selected bacterial groups Log CFU/g feces *Bacteroides-Prev.-Porphyr*. spp.: Int ↓ 0.18 log CFU/g, Pla ↑ 0.21 log CFU/g ^NS^ *Staphylococcus* spp.: Int ↓ 0.08 log CFU/g, Pla ↑ 0.16 log CFU/g ^NS^ *Cl. coccoides-Eubact. rectale* group*:* Int: ↓ 0.33 log CFU/g, Pla ↓ 0.01 log CFU/g ^NS^ *Lactobacillus* spp.: Int: ↑ 1.44 log CFU/g (*p* < 0.05), Pla ↓ 0.43 log CFU/g ^NS^ *Bifidobacterium* spp.: Int: ↑ 1.52 log CFU/g (*p* < 0.05), Pla ↑ 0.16 log CFU/g ^NS^ *Enterobacteriaceae:* Int: ↓ 0.14 log CFU/g, Pla ↑ 0.35 log CFU/g ^NS^
Valle et al. (96)	*n* = 65 RCT	Synbiotic vs. placebo	*Lactobacillus acidophilus LA-5; Bifidobacterium animalis BB-12* *2.3 g of inulin, 30 days*	16S gene sequencing *α–Diversity (Shannon index):* *Int:*↓ 0.125 *Pla:* ↑*0.027 ^*NS*^* *α–Diversity (Simpson index): Int:* ↓*0.017, Pla:* ↑*0.01 ^*NS*^*
West et al. (98)	*n* = 22 RCT	Synbiotic vs. prebiotic	*L. paracasei* subs *Paracasei* (*L. casei* 431^®^), *B. animalis* ssp lactis (BB-12^®^), *L. acidophilus* LA-5, *L. rhamnosus* GG, Raftiline, Raftilose GR, 3 wk	*Microbiome Diversity (16SrRNA)* ^NS^ changes in bacterial diversity (data not shown) *All data reported as raw bacterial counts*. Total bacteria: Syn ↔ 0, ^NS^, Pre ↑ 2 × 10^8^ Total *Lactobacillus* (mean): Syn ↔ 0, Pre ↑ 1.5 × 10^4^ ^NS^ L. *paracasei* (mean): Syn ↑ 8 × 10^2^, Pre ↓ 2 × 10^2^ (“large” 9-fold difference) B. *lactis* (mean): Syn ↑ 2.7 × 10^4^, Pre ↑ 4.8 × 10^3NS^
**Short chain fatty acids**				
Damen et al. (55)	*n* = 27 RXT	Prebiotic vs. placebo	Arabinoxylan oligosaccharides (AXOS), 3 wk	*All data reported in μmol/g wet feces* Total SCFA: Int: ↑ 25.3 (*p* < 0.05), Pla ↑ 9.6 ^NS^ Acetic acid: Int: ↑ 10.2, Pla ↑ 4.8^NS^ Butyric acid: Int: ↑ 7.6 (*p* < 0.05), Pla ↑ 2.3^NS^ Propionic acid: Int: ↑ 3.2, Pla ↑ 1.3^NS^
Finegold et al. (56)	*n* = 32 RCT	Prebiotic vs. Prebiotic vs. placebo	High and Low dose Xylooligosaccharides (XOS), 8 wk	Total SCFA (μmol/g dry feces): High ↓ 0.01, Low ↓ 0.06, Pla ↓ 0.06^NS^
François et al. (57)	*n* = 63 RXT	Prebiotic vs. Prebiotic vs. placebo	Arabinoxylan oligosaccharides (AXOS), 3 wk	*All data reported in μmol/g dry feces* Total SCFA: High vs. Pla: ↑ 53.1 (*p* = 0.001), Low vs. Pla: ↑ 7.8 ^NS^ Acetic acid: High vs. Pla: ↑ 38.5 (*p* = 0.003), Low vs. Pla: ↑ 8.9 ^NS^ Butyric acid: High vs. Pla: ↑ 5.0 (*p* = 0.05), Low vs. Pla: ↓ 3.9 ^NS^ Propionic acid: High vs. Pla: ↑ 9.7 (*p* = 0.003), Low vs. Pla: ↑ 2.9 ^NS^
Kleessen et al. (59)	*n* = 45 RCT	Prebiotic vs. prebiotic vs. placebo	Chicory inulin (CH) or Jerusalem artichoke inulin (JA), 3 wk	Total SCFA post-intervention (μmol/g wet feces): CH: 142.4, JA: 135.2, Pla: 138.8 ^NS^
Reimer et al. (60)	*n* = 48 RXT	Prebiotic vs. Prebiotic vs. placebo	Chicory inulin type fructans (ITF), 4 wk	Fecal acetate Int 1: ↑ 2.9 umol/g, Con 1: ↑ 6.7 umol/g ^NS^ Int 2: ↑ 6.7 umol/g, Con 2: ↑ 9.2 umol/g ^NS^ Fecal proprionate Int 1: ↑ 3.8 umol/g, Con 1: ↑ 2.4 umol/g ^NS^ Int 2: ↓ 1.3 umol/g, Con 2: ↑ 1.3 umol/g ^NS^ Fecal butyrate Int 1: ↑ 2.5 umol/g, Con 1: ↑ 0.3 umol/g ^NS^ Int 2: ↑ 4.4 umol/g, Con 2: ↑ 6.7 umol/g ^NS^ Fecal Isobutyrate Int 1: ↔ 0 umol/g, Con 1: ↑ 0.6 umol/g ^NS^ Int 2: ↔ 0 umol/g, Con 2: ↔ 0 umol/g ^NS^ Fecal Isovalerate Int 1: ↔ 0 umol/g, Con 1: ↔ 0 umol/g ^NS^ Int 2: ↔ 0 umol/g, Con 2: ↑ 0.4 umol/g ^NS^
Huang et al. (74)	*n* = 20 male RCT	Probiotic vs. placebo	*L. plantarum PS128, 4 wk*	Acetic acid (mean, post only): Int: 4.7 ng/ml, Pla: 3.8 ng/ml Int > Pla (*p* < 0.05) Proprionic acid (mean, post only): Int: 1.18 ng/ml, Pla: 0.5 ng/ml Int > Pla (*p* < 0.05) Butyric acid (mean, post only): Int: 0.5 ng/ml, Pla: 0.3 ng/ml Int > Pla (*p* < 0.05) Decanoic acid (mean, post only): Int: 0.005 ng/ml, Pla: 0.002 ng/ml ^NS^ Heptanoic acid (mean, post only): Int: 0.6 ug/ml, Pla: 0.4 ug/ml ^NS^ Hexanoic acid (mean, post only): Int: 1.7 ug/ml, Pla: 4.0 ug/ml ^NS^ Isobutyric acid (mean, post only): Int: 0.050 ng/ml, Pla: 0.052 ng/ml ^NS^ Isovaleric acid (mean, post only): Int: 0.03 ng/ml, Pla: 0.04 ng/ml ^NS^ Octanoic acid (mean, post only): Int: 1.1 ug/ml, Pla: 0.7 ug/ml ^NS^ Valeric acid (mean, post only): Int: 0.07 ng/ml, Pla: 0.07 ng/ml ^NS^
Son et al. (84)	*n* = 15 RCT	Probiotic vs. placebo	*L. acidophilus, L. casei, L. helveticus, B. bifidum, 60 days*	Acetic acid Int: ↓ 40 umol/g, Pla: ↓ 85 umol/g ^NS^ Buytric acid Int: ↓ 142 umol/g, Pla: ↑ 125 umol/g Int > Pla at baseline (*p-*value not shown) Propionic acid Int: ↓ 1.31 umol/g, Pla: ↓ 1.51 umol/g ^NS^
Klein et al. (75)	*n* = 26 RXT	Probiotic vs. placebo	*B. lactis* 420 × , *L. acidophilus* 74-2, 5 wk	*All data post-intervention concentration (μmol/g feces)* Total SCFAs: Int 85.0 μmol/g, Pla 88.5 μmol/g ^NS^ Acetic acid Int: 46.7 μmol/g, Pla 49.5 μmol/g ^NS^ *i*-Butyric acid Int 1.9 μmol/g, Pla 2.0 μmol/g ^NS^ n-Butyric acid Int 14.6 μmol/g, Pla 15.1 μmol/g ^NS^ Propionic acid Int: 16.7 μmol/g, Pla 16.9 μmol/g ^NS^ Valeric acid: Int: 2.1 μmol/g, Pla 2.0 μmol/g ^NS^ Isovaleric acid: Int: 2.3 μmol/g, Pla 2.4 μmol/g ^NS^ Caproic acid: Int: 0.7 μmol/g, Pla 0.6 μmol/g ^NS^
Valle et al. (96)	*n* = 65	Synbiotic vs. placebo	*Lactobacillus acidophilus LA-5; Bifidobacterium animalis BB-12* *2.3 g of inulin, 30 days*	Fecal acetate (mmol/L): Int: OR 0.34, 95%CI −0.06, 0.74 Pla: OR 0.16, 95%CI −0.25, 0.57 ^NS^ between groups
				Fecal proprionate (mmol/L): Int: OR 0.20, 95%CI −0.01, 0.41 Pla: OR 0·31, 95%CI −0.02, 0.63 ^NS^ between groups Fecal butyrate (mmol/L): Int: OR 0.39, 95%CI 0.20, 0.59 Pla: OR 0.25, 95%CI −0.03, 0.47 ^NS^ between groups Fecal ammonia (mmol/l): Pla: OR 0.09, 95%CI 0.01, 0.17 Int: OR 0.11, 95%CI 0.04, 0.18 ^NS^ between groups
West et al. (98)	*n* = 22 RCT	Synbiotic vs. prebiotic	*L. paracasei* subs Paracasei (*L. casei* 431^®^), *B. animalis* ssp. lactis (BB-12^®^), *L. acidophilus* LA-5, *L. rhamnosus* GG, Raftiline, Raftilose GR, 3 wk	*All data reported in μmol/g feces* Acetic acid: Syn ↓ 2, Pre ↓ 6 ^NS^ Butyric acid: Syn ↓ 2, Pre ↓ 3 ^NS^ Propionic acid: Syn ↓ 2, Pre ↓ 1.5 ^NS^
**Gastrointestinal symptoms**				
Damen et al. (55)	*n* = 27 RXT	Prebiotic vs. placebo	Arabinoxylan oligosaccharides (AXOS), 3 wk	GIS: Insufficient incidence to analyze abdominal pain or bloating. Flatulence: Int: ^NS^ difference pre-to-post Pla: ↑ 0.53 on 0–4 scale pre-to-post (*P* = 0.02)
Finegold et al. (56)	*n* = 32 RCT	Prebiotic vs. Prebiotic vs. placebo	Xylooligosaccharides (XOS), 8 wk	*Symptoms rated from 0 (no symptoms) to 3 (severe)* Excess flatus: High ↑ 0.27, Low ↑ 0.26, ↑ Pla 0.19 ^NS^ Borborygmi: High ↑ 0.26, Low ↑ 0.11, ↑ Pla 0.02 ^NS^ Bloating: High ↑ 0.28, Low ↑ 0.22, ↑ Pla 0.06 ^NS^ Abdominal pain: High ↓ 0.01, Low ↑ 0.27, ↑ Pla 0.10 ^NS^
François et al. (57)	*n* = 63 RXT	Prebiotic vs. Prebiotic vs. placebo	Arabinoxylan oligosaccharides (AXOS), 3 wk	↑ Occurrence frequency + ↑ Distress severity, flatulence only cf Pla (*P* = 0·02) Flatulence (*mild/moderate/very disturbing symptoms, %*): High 27/7/2, Low 16/7/2, Pla 11/6/2 ^NS^ ^NS^ all other symptoms.
Kleessen et al. (59)	*n* = 45 RCT	Prebiotic vs. prebiotic vs. placebo	Chicory inulin (CH) or Jerusalem artichoke inulin (JA), 3 wk	*All data reported as incidence (%) post-supplementation* Flatulence: CH 87, JA 93, Pla 47 (CH and JA > Pla, *p* < 0.05) Abdominal bloating: CH: 0, JA: 27, Pla: 27 ^NS^ Abdominal pain or cramps: CH: 20, JA: 7, Pla: 7 ^NS^ Bowel Rumbling: CH: 13, JA: 13, Pla: 13 ^NS^ Bowel Cramps: CH: 20, JA: 20, Pla: 13 ^NS^
Reimer et al. (60)	*n* = 48 RXT	Prebiotic vs. Prebiotic vs. placebo	Chicory inulin type fructans (ITF), 4 wk	Abdominal pain, 0–4 Int 1: 0.3 ± 0.2, Con 1: 0.3 ± 0.2 ^NS^ Int 2: 0.4 ± 0.2, Con 2: 0.3 ± 0.2 ^NS^ Distension/bloating (0–4) Int 1: 0.5 ± 0.3, Con 1: 0.4 ± 0.2 (*P* = 0.025) Int 2: 0.6 ± 0.2 (*P* = 0.023), Con 2: 0.2 ± 0.1 (*P* = 0.048) ^NS^ between groups Flatulence (0–4) Int 1: 0.3 ± 0.2, Con 1: 0.3 ± 0.2 ^NS^ Int 2: 0.3 ± 0.2, Con 2: 0.0 ± 0.2 ^NS^ Stomach rumbling (0–4) Int 1: 0.1 ± 0.2, Con 1: 0.1 ± 0.2 ^NS^ Int 2: 0.2 ± 0.2, Con 2: 0.1 ± 0.1 ^NS^
Russo et al. (45)	*n* = 20 RXT	Prebiotic vs. placebo	Inulin-enriched pasta, 5 wk	^NS^Differences and no major symptoms (data not reported)
Gleeson et al. (70)	*n* = 58 RCT	Probiotic vs. placebo	L. casei Shirota, 16 wk	GIS Incidence: Int 54%, Pla 57% ^NS^ Proportion of days with GIS: Int 2%, Pla 3% (*p* = 0.008)
				Severity Score: Int 9, Placebo 12 ^NS^ Symptom duration (days): Int 4.2, Pla 5.9 ^NS^
Haywood et al. (72)	*n* = 30 RCT	Probiotic vs. placebo	*L. gasseri, B. bifidum, B. longum*, 4 wk	GIS Incidence: Int: 13%, Pla: 13% ^NS^
Pugh et al. (79)	*n* = 24	Probiotic vs. placebo	*L. acidophilus* CUL60, *L. acidophilus* CUL21, *B. bifidum* CUL20, *B. animalis* subsp. *Lactis* CUL34, 4 wk	Number of GI scores ≥ 4: Days 1–14: Int 4, Pla 5^NS^ Days 15–28: Int 2, Pla 11 ^NS^
Schreiber et al. (82)	*n* = 27 RCT	Probiotic vs. placebo	*L. helveticus* Lafti L10, *B. animalis* ssp. lactis Lafti B94 *E. faecium* R0026, *B. longum* R0175 *Bacillus subtilis* R0179, 90 days	GIS incidence at rest (ΔGI): Int: −30 ± 48%, Pla: −27 ± 47% ^NS^ Categorized as: Nausea incidence at rest (ΔGI): Int −16 ± 43%, Pla: 71 ± 119% Int < Pla, *P* = 0.01, *d* = 0.9 Belching incidence at rest (ΔGI): Int: −14 ± 53%, Pla: 62 ± 115%, Int < Pla, *P* = 0.04, *d* = 1 Vomiting incidence at rest (ΔGI): Int: −7 ± 30%, Pla: 49 ± 114%, Int < Pla, *P* = 0.04, *d* = 0.7 Other sub-categories not reported.
Strasser et al. (85)	*n* = 29 RCT	Probiotic vs. placebo	*Bifidobacterium bifidum* W23 *Bifidobacterium lactis* W51 *Enterococcus faecium* W54 *Lactobacillus acidophilus* W22 *Lactobacillus brevis* W63 *Lactococcus lactis* W58 12 wk	Incidence: “Only one participant in the placebo group experienced GI-discomfort symptoms during the study period.”
West et al. (90)	*n* = 88 RCT	Probiotic vs. placebo	*Lactobacillus fermentum* VRI-003 PCC^®^, 11 wk	No. of GIS episodes: Male: Int 1.01, Pla: 0.49 (Likely ↑) Female: Int 1.44, Pla 0.48, (Likely ↑) Duration of GIS episodes (days): Male: Int 3.3, Pla 1.3, (Likely ↑) Female: Int 3.9, Pla 2.1, (Possible ↑) GIS severity (1–3 scale): Male: Int 1.31, Pla 1.78 (Possible ↓) Female: Int 1.44, Pla 1.75 (Possible ↓) Symptom Load (severity-days): Male: Int 4.4, Pla 2.5 (Possible ↑) Female: Int 5.2, Pla 2.9 (Possible ↑)
Coman et al. (92)	*n* = 10 RCT	Synbiotic vs. placebo	*L. rhamnosus* IMC 501[R], *L. paracasei* IMC 502[R], oat bran fiber, 4 wk	*Data reported as change from baseline, Likert scale (0–5)* Intestinal regularity: Int ↑ 2.6, Pla ↑ 1.8 (*p* < 0.05) Stool volume: Int ↑ 1.8, Pla ↑ 2.2^NS^ Ease at defecation: Int ↑ 2.2, Pla ↑ 0.6 (*p* < 0.05) Bloating: Int ↑ 0.2, Pla ↑ 0.4^NS^ Abdominal pain: Int ↔ 0.0, Pla ↓ 0.2^NS^ Intestinal cramps: Int ↔ 0.0, Pla ↓ 0.4^NS^
Roberts et al. (94)	*n* = 20 RCT	Synbiotic vs. placebo	*L. acidophilus* CUL-60 (NCIMB 30157), *L. acidophillus* CUL-21 (NCIMB 30156), *B. bifidum* CUL-20, *B. animalis* subspecies *lactis* CUL-34 (NCIMB 30153), Fructooligosaccharides, 12 wk	Mean total GIS score during supplemental period: Int 7.00, Pla 13.9 (*p* < 0.001) Mean GIS severity score during supplemental period: Int 8.00, Pla 16.7 (*p* < 0.001)
Valle et al. (96)	*n* = 65	Synbiotic vs. placebo	*Lactobacillus acidophilus LA-5; Bifidobacterium animalis BB-12* *2·3 g of inulin, 30 days*	Sum of symptoms (nausea, vomiting, diarrhea, abdominal pain, flatulence, loss of appetite, burning and dysphagia) Int: Δ Post-supp.: OR −2·24, 95%CI −3·15, −1·34 Pla: Δ Post-supp: OR −1·16, 95%CI −2·51, 0·18 ^NS^Between groups.

CFU, colony forming units; CRP, c-reactive protein; FISH, fluorescence *in situ* hybridization; GIS, Gastrointestinal symptoms; IFN, interferon; IL, interleukin; Int, Intervention; LBP, lipopolysaccharide binding protein; LPS, lipopolysaccharide; NS, Not significant; OTU, operational taxonomy units; Pla, Placebo; Pre, Prebiotic intervention; RCT, Randomized control trial; RXT, Randomized crossover trial; Syn, Synbiotic intervention; wk, weeks.

### Systemic bacterial endotoxin profile at rest

*N* = 4 studies assessed systemic endotoxin responses pre- and post- supplementation period (Tables 3, 4). LPS and LBP were not influenced by 6 weeks supplementation with *B. longum* 35624 (88), or following 2 weeks multi-strain supplementation with *S. thermophilus, L. delbrueckii* spp. *bulgaricus* and *L. rhamnosus* GG (66). Gram negative endotoxin units (using a *Limulus* amoebocyte lysate endotoxin kit) (66) and anti-LPS endotoxin-core antibodies (i.e., IgG) were unaffected by 12 weeks of a synbiotic (multi-strain probiotic plus fructo-oligosaccharide) supplementation (94). Seven days of supplementation with *L. casei* (strain not specified) resulted in no change in plasma gram negative endotoxin concentration and no difference compared with placebo (68).

### Systemic inflammatory cytokine profile at rest

*N* = 14 studies assessed systemic inflammatory cytokine responses or systemic inflammatory regulating factors, before and after the supplementation period (Tables 3, 4); of which, *n* = 12 used probiotics (63, 65–67, 69, 73, 76, 82, 83, 87–89) and *n* = 2 synbiotic (93, 98) as the intervention. Of these, *n* = 3 studies observed a positive effect of probiotic supplementation compared with placebo. An attenuated rise in C-C Motif Chemokine Ligand 2 (CCL2) was observed following 2 weeks supplementation with *S. thermophilus, L. delbrueckii* spp. *Bulgaricus*, and *L. rhamnosus* GG, compared with placebo (66). An attenuated rise in tumor necrosis factor alpha (TNF-α) was observed following 12 weeks supplementation with *B. subtilis*, compared with placebo (88). A significant reduction was observed in interleukin (IL)-2 and IL-10 compared with baseline in the intervention group only, and a significant drop in IL-4 was observed in the placebo group only compared with baseline, following 30 days of supplementing with *Lactobacillus acidophilus-LB-G80, Lactobacillus paracasei-LPc-G110, Lactococcus* subp. *lactis-LLL-G25, Bifidobacterium animalis* subp. *lactis-BL-G101* and *Bifidobacterium bifidum-BB-G90* (87). A multi-strain synbiotic (*L. paracasei* subs *Paracasei* (*L. casei* 431^®^), *B*. *animalis* ssp. *lactis* (BB-12^®^), *L. acidophilus* LA-5, *L. rhamnosus* GG, *Raftiline*, and *Raftilose* GR) for 3 weeks resulted in a 50% lower increase in circulating IL-16 concentration, compared to a prebiotic control (i.e., acacia gum) (98). One study with a multi-strain synbiotic (*B. lactis* CBP-001010, *L. rhamnosus* CNCM I-4036, *B. longum* ES1, and Fructooligosaccharides) for 30 days reported greater reduction in circulating IL-10 concentration in the placebo than the intervention group (93). Otherwise, no other effects on resting systemic inflammatory cytokines were reported.

### Gastrointestinal functional markers at rest

*N* = 4 studies, all prebiotic supplementation interventions, reported outcomes relating to gastrointestinal functional responses at rest (55, 59–61) (Tables 3, 4). *N* = 1 study reported a reduction in frequency of bowel movements with the consumption of bread fortified with arabinoxylan oligosaccharides (AXOS) (55). Five weeks consumption of inulin enriched pasta increased ultrasound-measured gastric half emptying time at rest by a median 8.3 min, and full emptying time by a median 30 min, with no effect observed with placebo (45). Presumably the same intervention reported in a separate paper (61), a significantly greater increase in the median proportion of normal resting slow waves (i.e., normogastria) from pre- to post-intervention with electrogastrography (EGG) was observed. No other effects of supplementation intervention were observed on gastrointestinal functional markers.

### Gastrointestinal symptoms at rest

The incidence of GIS throughout the period of supplementation was measured in *n* = 16 studies; of which, *n* = 6 utilized prebiotic (45, 55–57, 59, 60, 62), *n* = 7 probiotic (70, 72, 79, 82, 85, 90, 99), and *n* = 3 synbiotic (92, 94, 96) interventions (Tables 3, 4). Prebiotics supplementation did not influence GIS incidence at rest, other than a doubling of flatulence during 28 days of chicory or Jerusalem artichoke inulin supplementation, compared to placebo (59). A mild increase in flatulence was also seen following high dose AXOS bread consumption for 3 weeks, compared with placebo (57). In contrast with this, following 3 weeks of consumption of AXOS fortified bread, flatulence was only increased during the control bread period (55). Probiotic supplementation in all but two studies, did not influence GIS incidence at rest. Supplementation with *L. fermentum* VRI-003 PCC^®^ for 11 weeks increased GIS incidence, and also resulted in a ~2-day increased duration of symptoms, but a small reduction in symptom severity (90). Whilst overall GIS incidence at rest remained unchanged following 90 days of supplementation with a multistrain probiotic (*Lactobacillus helveticus Lafti L10, Bifidobacterium animalis ssp. lactis Lafti B94 Enterococcus faecium, Bifidobacterium longum R0175* and *Bacillus subtilis R0179*), the subcategories of nausea, belching and vomiting were significantly lower in the probiotic group compared to placebo (82). The majority of participants in the synbiotic studies reported no GIS incidence at rest (96). However, *n* = 1 study observed GIS severity scores at rest that were approximately half of those reported following placebo supplementation, following 12 weeks of a multi-strain synbiotic with added fructooligosaccharides (94). Increased intestinal regularity and ease of defecation was also reported following 4 weeks supplementation with *L. rhamnosus* IMC 501[R], *L. paracasei* IMC 502[R], plus oat bran fiber (92).

### Fecal microbial composition at rest

A total of *n* = 17 studies reported on resting microbial composition of stool samples (i.e., representative of luminal microbial composition) before and after the supplementation intervention period, of which *n* = 5 prebiotic (55–57, 59, 60), *n* = 9 probiotic (63, 66, 74, 75, 77, 78, 80, 84, 90), and *n* = 3 synbiotic interventions (92, 96, 98) (Tables 3, 4). Supplementation with xylooligosaccharides (XOS) (56), high dose (8.0 g/d) AXOS (57), chicory or Jerusalem artichoke inulin (59) or moderate dose (7.0 g/d) chicory inulin type fructans (60) resulted in significant increases in *Bifidobacterium* relative abundance (i.e., proportion of total bacterial counts), with a trend for the same effect in the fourth study (55) as determined by 16S rRNA sequencing (56, 60), and fluorescent *in situ* hybridization (FISH) analysis (57, 59). In contrast, low dose chicory inulin type fructan supplementation showed no increase in *Bifidobacterium* relative abundance (60). AXOS supplementation was also observed to increase *Bacteroides fragilis* group count, and total anaerobes count, compared to baseline (56). Reductions in *Bacteroides/Prevotella* were observed for both chicory and Jerusalem artichoke inulin supplementation, as assessed by grams of wet feces (59). Moderate dose chicory inulin-type fructan supplementation showed increases in microbial abundance at the phylum level in *Actinobacteria*, with reductions in *Firmicutes* and *Bacteroidetes*, with non-significant changes in the low dose group compared with control (60). At the family level, moderate dose chicory inulin-type fructan supplementation resulted in increases in *Bifidobacteriaceae, Actinomycetaceae, Microbacteriaceae, Cellulomonadaceae, Micrococcaceae*, and *Brevibacteriaceae*, but this effect was not observed in low doses of the supplement. At the genus level, increases were observed in *Bifidobacterium, Actinomyces, Cellulomonas*, and *Nesterenkonia*, with reductions in *Lachnospira, Oscillospira*, and *Brevibacterium* following moderate dose chicory inulin-type fructan supplementation (60). No effects of prebiotic supplementation were seen for other fecal sample bacterial taxa analyses, including α-diversity (56, 60).

Of the probiotic studies, *n* = 4 demonstrated significant increases in the supplemented species (*S. salivarius* spp. *thermophilus and L. delbrueckii* spp. *Bulgaricus, B. lactis* 420 × and *L. acidophilus, L. fermentum* VRI-003 PCC^®^, and *B. longum* subsp. *Longum*) (66, 75, 78, 90) as assessed by FISH analysis (75), and 16S gene sequencing (66, 78, 90). The only other species-level change reported was a reduction in *Fusiformis* following 4 weeks of supplementation with *L. salivarius* UCC118 (63), with no other significant changes in bacterial species reported (66, 75, 78, 84). At the phylum level, a decrease was observed in *Verrucomicrobia*, following 4 weeks of supplementation with *L. salivarius* UCC118 (63). Five weeks of supplementation with *Bifidobacterium longum* subsp. *Longum* showed a greater abundance of *Actinobacteria* and *Firmicutes*; with a reduction in *Proteobacteria*, albeit statistical significance or lack thereof was not reported (78). No other changes were observed at the phylum level following probiotic supplementation (74, 78, 94). Genus level changes observed following probiotic supplementation include a reduction in *Prosthecobacter* following 4 weeks of supplementation with *L. salivarius* UCC118 (63). A significant increase in *Bifidobacterium and Lactobacillus* was observed following 5 weeks of supplementation with *Bifidobacterium longum subsp. Longum* (78). However, 4 weeks of supplementation with a multi-strain probiotic (*L. paracasei, L. rhamnosus, L. helveticus, L. fermentum*, and *S. thermophilus)*, showed no change in *Lactobacillus* and a comparative decrease in *Bifidobacterium* (77). Four weeks of supplementation with *L. plantarum* showed a lower relative abundance in *Anaerotruncus, Caproiciproducens, Coprobacillus, Desulfovibrio, Dielma, Family_XIII_UCG_001, Holdemania*, and *Oxalobacter* compared with placebo. In addition, a greater relative abundance in *Akkermansia, Bifidobacterium, Butyricimonas*, and *Lactobacillus* was observed, however baseline data was not reported (74). Probiotic supplementation did not result in any further changes in other fecal sample bacterial taxa groups, including α-diversity (63, 80, 84, 90). The *n* = 2 synbiotic studies demonstrated increases in the genus of some of the supplemented strains, reported as raw bacterial count (62) or log CFU/g feces (92), but no differences in other determined bacterial groups (62, 92). No change in the α-diversity of the supplementation or placebo groups was detected in any of the synbiotic studies (96, 98).

### Fecal short chain fatty acid concentration at rest

Fecal SCFA concentrations were measured before and after supplementation in *n* = 9 included studies (Tables 3, 4), which included *n* = 5 prebiotic (55–57, 59, 60), *n* = 2 probiotic (75, 84), and *n* = 2 synbiotic interventions (96, 98). An additional probiotic study only reported post-intervention values for SCFA (74). *N* = 2 studies that provided 3 weeks AXOS supplementation demonstrated significant increases in total SCFA and butyric acid concentration (55, 57). Additionally, significant increases in acetic and propionic acid were observed following high (8.0 g/d), but not low dose (2.4 g/d) AXOS supplementation for 3 weeks in one study (57), but this finding was not replicated in another study that administered high dose (7.2 g/d) AXOS (55). No prebiotic intervention using XOS or inulin, resulted in increases in fecal SCFA (56, 59, 60). The figure presented in *n* = 1 multi-strain probiotic study appeared to show a drop in fecal butyric acid following 60 days of supplementation with *L. acidophilus, L. casei, L. helveticus*, and *B. bifidum*, compared with a rise in the placebo group (84). However, this study only reported a statistical difference at baseline, albeit with no indication of the *p*-value and did not report whether or not the changes over time were statistically significant. Another study reported greater concentrations in fecal acetic acid, propionic acid and butyric acid compared with the placebo group; however, whether these are genuine changes following supplementation cannot be determined as baseline data was not collected (74). No probiotic or synbiotic study observed and reported positive differences in SCFA concentrations at rest, as a result of supplementation.

### Studies assessing markers of exercise-induced gastrointestinal syndrome after a period of pre-, pro-, and syn-biotic supplementation

*N* = 13 studies reported at least one of the review outcomes, prior to, during, and/or following an acute exercise bout (Table 5). All except *n* = 2 studies investigated probiotic supplementation, the remaining papers investigated a synbiotic supplement intervention. No study of prebiotics being provided prior to an acute exercise bout was found in the search strategy. Probiotic supplements varied from single to multi-strain (i.e., up to nine different species), given either as capsules, powder sachets, or dairy-based beverage. The synbiotic supplements included two to four probiotic strains, plus additional fructo-oligosaccharides or inulin (94, 96). The acute exercise bouts varied substantially, and included *n* = 3 studies of treadmill running (either 2 h steady state with or without environmental heat exposure (e.g., 35°C), or time to exhaustion test) (63, 68, 100); *n* = 4 studies investigated supplementation prior to an outdoor marathon (65, 79, 87, 89); *n* = 3 studies on a cycle ergometer (either an incremental exercise test, time to fatigue tests or 2 h steady state cycle ergometer followed by 1 h time trial) (63, 90, 100); and, in one study participants completed an ultra-distance triathlon event (94). One study observed participant outcomes in response to 5 days of continuous intense military training, day and night including marching 8–30 km whilst carrying a pack up to 30 kg, sleep deprivation, and a range of environmental conditions (96). Another used online questionnaires to assess included measures (82). Outcome measures were taken prior to the exercise bout, but the timing of post-exercise measures varied from immediately to 6 days post-exercise. In all studies participants mean or median age was ≤ 42 years, and in all studies participants were from an endurance sport background (i.e., mean or median *V*O_2max_ range 47–64 ml/kg/min) or military training background.

**Table 5:** Systematic review search results, included studies that investigated the impact of prebiotic, probiotic and synbiotic supplementation on markers of EIGS and associated GIS in response to an acute exercise bout.

**References**	**Population and study design**	**Sample size determination**	**Supplementation protocol**	**Physical activity/dietary control**	**Exercise protocol**	**Outcome/s reported**
**Probiotic studies**						
Axelrod et al. (63)	*n* = 7 endurance runners, VO_2max_ 57.9 mL/kg/min RXT	A priori power analysis based on a previous investigation from healthy runners (64) estimated ~6 needed to obtain statistical power at the recommended 0.80 level based upon mean, between-groups comparison effect size (*d* = 1.2).	*L. salivarius* UCC118, 2 × 10^8^ CFU/cap 1 capsule daily for 4 wk	Normal lifestyle (not monitored)	Treadmill running: 2 h at 60% VO_2max._ T_amb_: 25°C, RH: 31%	Intestinal permeability Cytokine responses
Batatinha et al. (65)	*n* = 27 male marathon runners, age: Int: 35.96 ± 5.81; Pla: 40.46 ± 7.79 Fitness status not stated RCT	Not specified	*B. animalis. Lactis 10 × 10^9^; L. Acidophilus 10 × 10^9^* 1 sachet daily for 30 days	Training volume monitored, ^NS^ between groups. Dietary control not stated.	Marathon race: Race time was 4.08 ± 0.55 h T_abm_ not stated	Cytokine responses
Gill et al. (68)	*n* = 8 male runners and triathletes Age: 26 yrs VO_2max_ 59 ml/kg/min RXT	Based on the typical standard deviation of 0.7 EU/ml for circulatory endotoxin responses to exertional-stress (16, 69), and using standard alpha (0.05) and beta values (0.8) (www.dssresearch.com), a sample size of *n* = 8 is estimated to provide adequate statistical precision to detect a >10% difference in circulatory endotoxin concentration in response to EHS in the target population.	*L. casei*, 1.0 × 10^11^ cells/bottle Commercial supplement, taken twice daily for 7 days	Dietary recall. Activity control not stated.	Treadmill running: 2 h at 60% VO_2max._ T_amb_: 34°C, RH: 32%	Endotoxaemia Cytokine responses
Kekkonen et al. (99)	*n* = 119 (125M/16F recruited) marathon competitors, Best marathon time Int: 3:10 (2:35–3:42) Pla: 3:11 (2:23–3:40) Age 40 yrs. RCT	Not specified	Milk-based fruit drink or capsules (participant choice) containing *L. rhamnosus* GG, 4.0 × 10^10^ CFU 2 × 65 mL bottles or capsules daily for 3 months	Diary questionnaire with ready-made questions Training diaries, ^NS^ for running sessions or weekly distance.	Helsinki Marathon, 2003: Int: 3 h 32 min Pla: 3 h 30 min	GIS
Lamprecht et al. (76)	*n* = 23 male triathletes, runners and cyclists Age, Int: 37.6 yrs, Pla: 38.2 yrs VO_2max_: Int: 51.2 mL/kg/min Pla: 50.3 mL/kg/min RCT	Sample size calculation based on oxidation markers CP and MDA. Between 7 and 9 subjects estimated per group—depending on parameter, SD and effect size—to reach probability of error (alpha/2) of 5 and 80% power.	Ecologic^®^ Performance: *B. bifidum W23, B lactis W51, E. faecium W54, L. acidophilus W22, L. brevis W63, L. lactis W58*. 2 × 2 g sachets daily for 14 wk, providing (10^10^ CFU/day)	Habitual diet, food diary and repeated for 7 days prior to each exercise trial. Habitual training, no exercise 3 days prior to each exercise test.	3 × incremental “step” tests on cycling ergometer to exhaustion, with 15 min active recovery between each test. Total test time ~80–90 min. T_amb_: 20°C, RH: 60% RH	Cytokine responses
Pugh et al. (79)	*n* = 24 (20M/4F) marathon runners, age: Int: 34.8 ± 6.9 yrs Pla: 36.1 ± 7.5 yrs VO_2max_: Int: 57.6 ± 8.0 mL/kg/min Pla: 56.4 ± 8.6 mL/kg/min RCT	Not specified	Proven Probiotics Ltd., Port Talbot, Wales: *L. acidophilus* CUL60 *L. acidophilus* CUL21 *B. bifidum* CUL20 *B. animalis* subsp. *Lactis* CUL34 (). >25 billion CFU/cap 1 capsule daily for 4 wk	Dietary control not stated outside of acute exercise. Training not stated.	Non-sanctioned marathon (outdoor running track). Finish time: Int: 234 ± 38 min, Pla: 247 ± 47 ^NS^ % LT: Int: 90.2 ± 9.1, Pla: 91.3 ± 8.7 ^NS^	Intestinal permeability Intestinal injury Cytokine responses GIS
Pugh et al. (100)	*n* = 7 male cyclists Age: 23 ± 4 yrs VO_2peak_ 64.0 ± 2.2 mL/kg/min RXT	To detect a meaningful increase in exogenous CHO oxidation of 0.1 g/min with SD = 0.05 g/min (101) at 80% power, a minimum *n* = 5 required.	Proven Probiotics Ltd., Port Talbot, Wales: *L. acidophilus* CUL60 *L. acidophilus* CUL21 *B. bifidum* CUL20 *B. animalis* subsp. *Lactis* CUL34 (). >25 billion CFU/cap 1 capsule daily for 4 wk	Not stated	Cycle ergometer: 2 h at 55% W_max_ followed by 100 kJ time trial. Ambient conditions not stated	Intestinal permeability Intestinal injury Cytokine responses GIS
Schreiber et al. (82)	*n* = 27 male cyclists Age: Int: 25.9 ± 4.6 Pla: 29.5 ± 6.2 VO2_max_ (mL/kg/min) Int: 66.9 ± 6.4 Pla: 63.2 ± 5.0 RCT	Not specified	1 capsule containing: *L. helveticus* Lafti L10 (28.6%), *B. animalis* ssp. lactis Lafti B94 (28.6%), *E. faecium* R0026 (25.7%), *Bifidobacterium longum* R0175 (14.3%) *B. subtilis* R0179 (2.8%) 15 × 10^9^ CFU/cap 1 capsule daily for 90 days	Diet not controlled. “Difference in training hours during the study period” (Data not shown)	Evaluation (online survey) at training, competition, and during the first 2 h recovery from training or competition.	GIS
Shing et al. (102)	*n* = 10 male runners Age: 27 ± 2 yrs VO_2max_: 62.6 ± 2.1 mL/kg/min RXT	Sample size was determined to detect a treatment difference at a two-sided 5% significance level with a probability of 80% from primary outcome variables of lactulose/rhamnose and LPS. The lactulose:rhamnose ratio following exercise is reported to be 0.0625 (SD 0.0125) (103). Assuming that probiotics reduced the lactulose:rhamnose by 20% (similar to reduction following bovine colostrum supplementation) (104), a total of eight runners were required. Recent literature has shown a 20% increase in LPS concentration following running in the heat with an increase in training load (105). Assuming that probiotics reduced LPS concentration by 20% (post-exercise LPS concentration of 27 pg mL^−1^ with a within-subject standard deviation of 5 pg mL^−1^), a total of nine runners were required. Based on these calculations, 10 runners were recruited for the present study.	UltraBiotic45, BioCeuticals™: *L. acidophilus*, 7.4 × 10^9^ CFU/cap *L. rhamnosus*, 15.55 × 10^9^ CFU/cap *L. casei*, 9.45 × 10^9^ CFU/cap *L. plantarum*, 3.15 × 10^9^ CFU/cap *L. fermentum*, 1.35 × 10^9^ CFU/cap *B. lactis*, 4.05 × 10^9^ CFU/cap *B. breve*, 1.35 × 10^9^ CFU/cap *B. bifidum*, 0.45 × 10^9^ CFU/cap *S. thermophilus*, 2.25 × 10^9^ CFU/cap 1 capsule daily for 4 wk	Food diary ^NS^ between trials. Required to avoid strenuous exercise for 24 h prior to each testing session.	Treadmill running: time to fatigue at 80% ventilatory threshold. T_amb_: 35°C, RH: 40%	Intestinal permeability Intestinal injury Endotoxaemia Cytokine responses GIS
Tavares-Silva et al. (87)	*N* = 14 male marathon runners Age: Pla: 38.28 ± 3.09 Int: 41.57 ± 3.20 VO_2Peak_: Pla: 54.53 ± 6.88 kg/mL/min Int: 56.92 ± 8.35 kg/mL/min RCT	Not specified	*L. acidophilus-LB-G80, L. paracasei-LPc-G110, Lactococcus* subp. *lactis-LLL-G25*, *B. animalis* subp. *lactis-BL-G101, B. bifidum-BB-G90* Capsules containing 5 × 10^9^ CFU / day	Physical activity control not stated. Questionnaire 2 × /wk + once on weekends: Energy intake (kcal) Pla: 1,994.46 ± 365.73 Int: 2,434.69 ± 505.53 ^NS^ difference between groups	Marathon race Race Time (min) Pla: 243.0 ± 33.73 Int: 252.87 ± 39.77 ^NS^ Difference Tamb: 21.5°C, RH: 67%.	Cytokine responses
Vaisberg et al. (89)	*n* = 42 male marathon runners Age: Int: 39.6 ± 8.8 yrs, Pla: 40.1 ± 10.3 yrs VO_2max_: Int: 57.64 ± 6.89 mL/kg/min, Pla: 57.86 ± 6.85 mL/kg/min RCT	Not specified	*Lactobacillus casei Shirota*, 40 × 10^9^ live cells/bottle 1 × 80 g bottle daily for 30 days	Dietary control not stated. Instructed to keep usual training/physical exercise schedules—not reported	Marathon race Ambient conditions not stated	Cytokine responses
West et al. (90)	*n* = 88 cyclists and triathletes Age: Int: M: 35.2 yrs, F: 36.5 yrs, Pla: M: 36.4 yrs, F 35.6 VO_2max_ (mL/kg/min): Int: M: 56.5, F: 57.6 Pla: M: 55.8, F: 51.6 RCT	A sample size of 80 subjects was required for identifying substantial changes in the incidence of illness, assuming a rate of URTI symptoms of 60% in the placebo group, with sufficient power (86% at an alpha-level of 0.05) to detect a 50% reduction in symptoms.	*Lactobacillus fermentum* VRI-003 PCC^®^, 10^9^ CFU/cap 1 capsule daily for 11 wk	4 d food diary. Usual diet, without probiotic foods. Training log kept	Incremental cycling ergometer test (VO_2max_ protocol). Ambient conditions not stated	Cytokine responses
**Synbiotic studies**						
Roberts et al. (94)	*n* = 20 (9M/1F) long course triathletes VO_2max_: Int: 47.6 mL/kg/min Pla: 50.5 mL/kg/min RCT	Power calculation assessment for sample size (G*power3, Dusseldorf (95); using α = 0.05; 1 – β = 0.80; based on observed data.	Bio-Acidophilus Forte, Biocare Ltd., Birmingham, UK): *L. acidophilus* CUL-60 (NCIMB 30157), 10^10^ CFU/cap *L. acidophillus* CUL-21 (NCIMB 30156), 10^10^ CFU/cap *B. bifidum* CUL-20 (NCIMB 30172), 9.5^10^ CFU/cap *B. animalis* subspecies *lactis* CUL-34 (NCIMB 30153), 0.5^10^ CFU/cap. Fructooligosaccharides, 55.8 mg per cap 1 capsule daily for 12 wk	Habitual diet, food diary first and last wk of each month. ^NS^ between groups or over intervention time period. Prescribed triathlon training program, individualized. ^NS^ between groups for training load throughout intervention period.	Ironman triathlon. Mean finish time: Int: 12 h 47 min, Pla: 14 h 12 min ^NS^	Endotoxin responses Intestinal permeability
Valle et al. (96)	*n* = 65 (39M/26F) military personnel Age: Pla: 19.5 ± 1.22, Int: 19.69 ± 1.25 Fitness status not stated RCT	Sample calculation in G * Power 3.1.9.2 software was based on the following data: 5% sample error, 95% CI and 0.72 effect size considering pre and post-intervention IgA values. The effect size was estimated based on the study by Olivares et al. (97).	*Lactobacillus acidophilus LA-5, 10·3 log CFU* *Bifidobacterium animalis BB-12, 11·0 log CFU* *Inulin, 2.3 g* *60g serve of ice cream, daily for 30 days*	Participants recommended not to consume any foods containing prebiotics and probiotics 15 d before the beginning of the research period, particularly over the weekend, when they are released to go home. During the week all food was provided. Not reported, however participants were undergoing military training.	5 d military training, including physical and psychological exhaustion, marching 8–20 km, carrying ~ 30 kg equipment, sleep deprivation. Subject to various weather extremes.	Bacterial taxa Fecal SCFA GIS

RCT, Randomized control trial; RXT, Randomized crossover trial; Cap, capsule; T_amb_, Ambient temperature; RH, Relative humidity; Int, Intervention; Pla, Placebo; NS, Not significant; wk, week.

### Intestinal epithelial injury in response to acute exercise

*N* = 3 studies (all probiotics) assessed the effect of the intervention on markers of intestinal injury, none of which observed differences in plasma or serum I-FABP concentration (79, 100), or urinary claudin-3 concentration (102) (Tables 5, 6).

**Table 6:** Systematic review study outcomes for included studies that investigated the impact of prebiotic, probiotic and synbiotic supplementation on markers of EIGS in response to an acute exercise bout.

**References**	***n* and study design**	**Supplement/** **comparator**	**Intervention ingredient/s and supplement duration**	**Exercise protocol**	**Outcome measure/s (Δ in mean/median from pre-exercise to immediately post-exercise unless otherwise indicated)**
**Intestinal permeability**					
Axelrod et al. (63)	*n* = 7 RXT	Probiotic vs. placebo	*L. salivarius* UCC118, 4 wk	Treadmill running: 2 h at 60% VO_2max._ Tamb: 25°C, RH: 31%	Sucrose permeability (Δ in iAUC from baseline): Int ↓ 38%, Pla ↑ 169% (*p* = 0.029) Rhamnose permeability (Δ in iAUC from baseline): Int ↓ 0.1-fold, Pla ↑ 0.5-fold ^NS^ Lactulose permeability (Δ in iAUC from baseline): Int ↓ 0.1-fold, Pla ↑ 0.4-fold ^NS^ L/R Ratio (Δ in iAUC from baseline): ^NS^ (data not reported)
Pugh et al. (79)	*n* = 24 RCT	Probiotic vs. placebo	*L. acidophilus* CUL60 *L. acidophilus* CUL21 *B. bifidum* CUL20 *B. animalis* subsp. *Lactis* CUL34, 4 wk	Non-sanctioned marathon (outdoor running track). Finish time: Int: 234 ± 38 min, Pla: 247 ± 47 ^NS^ % LT: Int: 90.2 ± 9.1, Pla: 91.3 ± 8.7 ^NS^ T_amb_: 16–17°C; Wind: 8–16 km/h	Serum L/R ratio (Δ from baseline test): Int ↑ 0.04, Pla ↑ 0.02 ^NS^
Pugh et al. (100)	*n* = 7 RXT	Probiotic vs. placebo	*L. acidophilus* CUL60 *L. acidophilus* CUL21 *B. bifidum* CUL20 *B. animalis* subsp. *Lactis* CUL34, 4 wk	Cycle ergometer: 2 h at 55% W_max_ followed by 100 kJ time trial. Ambient conditions not stated.	Serum L/R ratio (Δ from baseline test): Int ↑ 0.045, Pla ↑ 0.052 ^NS^
Shing et al. (102)	*n* = 10 RXT	Probiotic vs. placebo	*L. acidophilus, L. rhamnosus, L. casei, L. plantarum, L. fermentum, B. lactis, B. breve, B. bifidum, S. thermophilus*, 4 wk	Treadmill running: time to fatigue at 80% ventilatory threshold. Tamb: 35°C, RH: 40%	Urinary L/R ratio: 8% lower following Int cf Pla ^NS^–other data not reported
Roberts et al. (94)	*n* = 20 RCT	Synbiotic vs. placebo	*L. acidophilus* CUL-60 (NCIMB 30157), *L. acidophillus* CUL-21 (NCIMB 30156), *B. bifidum* CUL-20 (NCIMB 30172), *B. animalis* subspecies *lactis* CUL-34 (NCIMB 30153), Fructooligosaccharides, 12 wk	Ironman triathlon. Mean finish time: Int: 12 h 47 min, Pla: 14 h 12 min ^NS^	Urinary lactulose/mannitol ratio (Δ from pre- to 6 d post-race): Int ↑ 0.005, Pla ↑ 0.020 ^NS^
**Intestinal injury**					
Pugh et al. (79)	*n* = 24 RCT	Probiotic vs. placebo	*L. acidophilus* CUL60 *L. acidophilus* CUL21 *B. bifidum* CUL20 *B. animalis* subsp. *Lactis* CUL34, 4 wk	Non-sanctioned marathon (outdoor running track). Finish time: Int: 234 ± 38 min, Pla: 247 ± 47 ^NS^ % LT: Int: 90.2 ± 9.1, Pla: 91.3 ± 8.7 ^NS^ T_amb_: 16–17°C; Wind: 8–16 km/h	Serum I-FABP: Int ↑ 1,359 pg/mL, Pla ↑ 932 pg/mL ^NS^
Pugh et al. (100)	*n* = 7 RXT	Probiotic vs. placebo	*L. acidophilus* CUL60 *L. acidophilus* CUL21 *B. bifidum* CUL20 *B. animalis* subsp. *Lactis* CUL34, 4 wk	Cycle ergometer: 2 h at 55% W_max_ followed by 100 kJ time trial. Ambient conditions not stated.	Plasma I-FABP: Post-exercise: Int ↓ 207 pg/mL, Pla ↓ 295 pg/mL ^NS^ 1 h post-exercise: Int ↓ 182 pg/mL, Pla ↓ 263 pg/mL ^NS^
Shing et al. (102)	*n* = 10 RXT	Probiotic vs. placebo	*L. acidophilus, L. rhamnosus, L. casei, L. plantarum, L. fermentum, B. lactis, B. breve, B. bifidum, S. thermophilus*, 4 wk	Treadmill running: time to fatigue at 80% ventilatory threshold. Tamb: 35°C, RH: 40%	Urinary Claudin 3 (absolute values): Int 6.1 ± 3.3 ng/mmol creatinine, Pla 8.1 ± 5.1 ng/mmol creatinine ^NS^
**Bacterial endotoxin responses**					
Gill et al. (68)	*n* = 8 RXT	Probiotic vs. placebo	*L. casei*, 7 days	Treadmill running: 2 h at 60% VO_2max._ Tamb: 34°C, RH: 32%	Gram-negative bacterial endotoxin (ΔPre-ex - 1-h Post-ex): Int ↑ 0.5 EU/mL (23%), Pla ↓ 0.2 EU/mL (8%) (*p* = 0.05)
Shing et al. (102)	*n* = 10 RXT	Probiotic vs. placebo	*L. acidophilus, L. rhamnosus, L. casei, L. plantarum, L. fermentum, B. lactis, B. breve, B. bifidum, S. thermophilus*, 4 wk	Treadmill running: time to fatigue at 80% ventilatory threshold. Tamb: 35°C, RH: 40%	Serum LPS: Int ↑ 0.03 EU, Pla ↑ 0.05 EU ^NS^ Anti-LPS IgM: Int ↓ 1.0 MU/mL, Pla ↑ 0.3 MU/mL ^NS^
Roberts et al. (94)	*n* = 20 RCT	Synbiotic vs. placebo	*L. acidophilus* CUL-60 (NCIMB 30157), *L. acidophillus* CUL-21 (NCIMB 30156), *B. bifidum* CUL-20 (NCIMB 30172), *B. animalis* subspecies *lactis* CUL-34 (NCIMB 30153), Fructooligosaccharides, 12 wk	Ironman triathlon. Mean finish time: Int: 12 h 47 min, Pla: 14 h 12 min ^NS^	Endotoxin units (Δ from pre- to 6 d post-exercise): Int ↓ 1.6 pg/mL (*p* = 0.047), Pla ↓ 0.44 pg/mL ^NS^ Anti-LPS IgG (Δ from pre- to 6 d post-exercise): Int ↓ 90 MU/mL, Pla ↑ 27 MU/mL ^NS^
**Cytokine responses**					
Axelrod et al. (63)	*n* = 7 RXT	Probiotic vs. placebo	*L. salivarius* UCC118, 4 wk	Treadmill running: 2 h at 60% VO_2max._ Tamb: 25°C, RH: 31%	IL-6: (ΔΔ pre to post-exercise, pre to post-intervention) Int ↑ 0.5 pg/mL, Pla ↑ 1.4 pg/mL ^NS^
Batatinha et al. (65)	*n* =27 RCT	Probiotic vs. placebo	*B. animalis. Lactis 10 × 10^9^; L. Acidophilus 10 × 10^9^* 1 sachet daily for 30 days	Marathon race: Race time was 4.08 ± 0.55 h T_abm_ not stated	IL-10: Int: ↑ 254 ng/ml, Pla: ↑ 219 ng/ml ^NS^ IL-4: Int: ↑ 6.9 ng/ml, Pla:↑ 2.2 ng/ml ^NS^ IL-6: Int: ↑ 14.0 ng/ml, Pla: ↑ 14.1 ng/ml ^NS^ IL-2: Int: ↓ 2.6 ng/ml, Pla: ↓ 0.3ng/ml ^NS^ IL-15: Int: ↓ 0.7 ng/ml, Pla: ↓ 0.5 ng/ml ^NS^ IL-8: Int: ↑ 7.1 ng/ml, Pla: ↑ 10.4 ng/ml ^NS^ IL-1β: Int: ↓ 1.1 ng/ml, Pla: ↑ 0.1 ng/ml ^NS^ TNF-α: Int: ↑ 3.9 ng/ml, Pla: ↑ 3.8 ng/ml ^NS^ IFN-γ: Int: ↓ 5.3 ng/ml, Pla: ↓ 2.8 ng/ml ^NS^
Gill et al. (68)	*n* = 8 RXT	Probiotic vs. placebo	*L. casei*, 7 days	Treadmill running: 2 h at 60% VO_2max._ Tamb: 34°C, RH: 32%	IL-6: Post-exercise: Int ↑ 3.6 pg/mL, Pla ↑ 3.1 pg/mL ^NS^ 1 h post-exercise: Int ↑ 2.1 pg/mL, Pla ↑ 1.2 pg/mL ^NS^ 2 h post-exercise: Int ↑ 1.1 pg/mL, Pla ↑ 0.4 pg/mL ^NS^ 4 h post-exercise: Int ↑ 0.4 pg/mL, Pla ↔ 0.0 pg/mL ^NS^ 24 h post-exercise: Int ↔ 0.0 pg/mL, Pla ↓ 0.3 pg/mL ^NS^ IL-1β: Post-exercise: Int ↑ 0.09 pg/mL, Pla ↑ 0.02 pg/mL ^NS^ 1 h post-exercise: Int ↑ 0.03 pg/mL, Pla ↓ 0.03 pg/mL ^NS^ 2 h post-exercise: Int ↓ 0.01 pg/mL, Pla ↓ 0.01 pg/mL ^NS^ 4 h post-exercise: Int ↓ 0.01 pg/mL, Pla ↓ 0.01 pg/mL ^NS^ 24 h post-exercise: Int ↑ 0.02 pg/mL, Pla ↔ 0.0 pg/mL ^NS^ TNF-a: Post-exercise: Int ↑ 0.5 pg/mL, Pla ↑ 0.3 pg/mL ^NS^ 1 h post-exercise: Int ↑ 0.3 pg/mL, Pla ↓ 0.3 pg/mL ^NS^ 2 h post-exercise: Int ↔ 0.0 pg/mL, Pla ↓ 0.4 pg/mL ^NS^ 4 h post-exercise: Int ↑ 0.1 pg/mL, Pla ↓ 0.3 pg/mL ^NS^ 24 h post-exercise: Int ↑ 0.3 pg/mL, Pla ↑ 0.1 pg/mL ^NS^
					IFN-γ: Post-exercise: Int ↔ 0.0 pg/mL, Pla ↑ 0.1 pg/mL ^NS^ 1 h post-exercise: Int ↓ 0.1 pg/mL, Pla ↓ 0.2 pg/mL ^NS^ 2 h post-exercise: Int ↓ 0.3 pg/mL, Pla ↓ 0.4 pg/mL ^NS^ 4 h post-exercise: Int ↑ 0.3 pg/mL, Pla ↓ 0.4 pg/mL ^NS^ 24 h post-exercise: Int ↑ 0.2 pg/mL, Pla ↔ 0.0 pg/mL ^NS^ IL-10: Post-exercise: Int ↑ 10.8 pg/mL, Pla ↑ 7.2 pg/mL ^NS^ 1 h post-exercise: Int ↑ 10.6 pg/mL, Pla ↑ 9.0 pg/mL ^NS^ 2 h post-exercise: Int ↑ 2.8 pg/mL, Pla ↑ 2.7 pg/mL ^NS^ 4 h post-exercise: Int ↓ 0.1 pg/mL, Pla ↔ 0.0 pg/mL ^NS^ 24 h post-exercise: Int ↔ 0.0 pg/mL, Pla ↑ 0.2 pg/mL ^NS^ IL-8: Post-exercise: Int ↑ 1.8 pg/mL, Pla ↑ 1.2 pg/mL ^NS^ 1 h post-exercise: Int ↑ 1.1 pg/mL, Pla ↑ 0.6 pg/mL ^NS^ 2 h post-exercise: Int ↑ 0.1 pg/mL, Pla ↑ 0.1 pg/mL ^NS^ 4 h post-exercise: Int ↔ 0.0 pg/mL, Pla ↓ 0.1 pg/mL ^NS^ 24 h post-exercise: Int ↓ 0.1 pg/mL, Pla ↓ 0.4 pg/mL ^NS^
Lamprecht et al.	*n* = 23 RCT	Probiotic vs. placebo	*B. bifidum W23, B lactis W51, E. faecium W54, L. acidophilus W22, L. brevis W63, L. lactis W58*, 14 wk	3 x incremental “step” tests on cycling ergometer to exhaustion, with 15 min active recovery between each test. Total test time ~80–90 min. T_amb_: 20°C, RH: 60% RH	IL-6: Int ↑ 3.3 pg/mL, Pla ↑ 1.9 pg/mL ^NS^ TNF-α: Int ↑ 2.3 pg/mL, Pla ↑ 0.24 pg/mL ^NS^
Pugh et al. (79)	*n* = 24 RCT	Probiotic vs. placebo	*L. acidophilus* CUL60 *L. acidophilus* CUL21 *B. bifidum* CUL20 *B. animalis* subsp. *Lactis* CUL34, 4 wk	Non-sanctioned marathon (outdoor running track). Finish time: Int: 234 ± 38 min, Pla: 247 ± 47 ^NS^ % LT: Int: 90.2 ± 9.1, Pla: 91.3 ± 8.7 ^NS^ T_amb_: 16–17°C; Wind: 8–16 km/h	Serum CD14: Int ↑ 5.9 μg/mL, Pla ↑ 5.4 μg/mL ^NS^ IL-6: Int ↑ 9.95 μg/mL, Pla ↑ 12.76 μg/mL ^NS^ IL-8: Int ↑ 11.21 μg/mL, Pla ↑ 9.98 μg/mL ^NS^ IL-10: Int ↑ 4.36 μg/mL, Pla ↑ 5.05 μg/mL ^NS^
Pugh et al. (100)	*n* = 7 RXT	Probiotic vs. placebo	*L. acidophilus* CUL60 *L. acidophilus* CUL21 *B. bifidum* CUL20 *B. animalis* subsp. *Lactis* CUL34, 4 wk	Cycle ergometer: 2 h at 55% W_max_ followed by 100 kJ time trial. Ambient conditions not stated.	IL-1α: Int ↑ 1.15 μg/mL, Pla ↑ 0.45 μg/mL ^NS^ IL-6: Int ↑ 1.05 μg/mL, Pla ↑ 1.37 μg/mL ^NS^ IL-8: Int ↑ 1.96 μg/mL, Pla ↑ 2.21 μg/mL ^NS^ IL-10: Int ↑ 2.11 μg/mL, Pla ↑ 1.18 μg/mL ^NS^
Shing et al. (102)	*n* = 10 RXT	Probiotic vs. placebo	*L. acidophilus, L. rhamnosus, L. casei, L. plantarum, L. fermentum, B. lactis, B. breve, B. bifidum, S. thermophilus*, 4 wk	Treadmill running: time to fatigue at 80% ventilatory threshold. Tamb: 35°C, RH: 40%	IL-1ra: Post-exercise: Int ↑ 74 pg/mL, Pla ↑ 79 pg/mL ^NS^ 1 h post-exercise: Int ↑ 183 pg/mL, Pla ↑ 188 pg/mL ^NS^ TNF-α: Post-exercise: Int ↑ 0.62 pg/mL, Pla ↑ 1.65 pg/mL ^NS^ 1 h post-exercise: Int ↓ 0.33 pg/mL, Pla ↑ 1.47 pg/mL ^NS^
					IL-6: Post-exercise: Int ↑ 0.91 pg/mL, Pla ↑ 1.32 pg/mL ^NS^ 1 h post-exercise: Int ↑ 1.04 pg/mL, Pla ↑ 1.45 pg/mL ^NS^ IL-10: Post-exercise: Int ↑ 1.96 pg/mL, Pla ↓ 0.22 pg/mL ^NS^ 1 h post-exercise: Int ↑ 7.61 pg/mL, Pla ↑ 9.89 pg/mL ^NS^ Neutrophil elastase: Post-exercise: Int ↓ 269 fg/cell, Pla ↑ 74 fg/cell ^NS^ 1 h post-exercise: Int ↓ 259 fg/cell, Pla ↓ 12 fg/cell ^NS^
Tavares-Silva et al. (87)	*n* = 14 RCT	Probiotic vs. placebo	*Lactobacillus acidophilus-LB-G80, Lactobacillus paracasei-LPc-G110, Lactococcus subp. lactis-LLL-G25, Bifidobacterium animalis subp. lactis-BL-G101, Bifidobacterium bifidum-BB-G90 5 × 10^9^ CFU 2.0 g/day for 30 days*	Marathon race Race Time (min) Pla: 243.0 ± 33.73 Int: 252.87 ± 39.77 ^NS^ Difference Tamb: 21.5°C, RH: 67%.	All changes relative to 24 h pre resting value IL-2 Post-ex: Pla: ↑ 0.01 pg/ml ^NS^, Int: ↑0.06 pg/ml ^NS^ 1 h post-ex.: Pla: ↓ 0.05 pg/ml ^NS^, Int: ↑ 0.11 pg/ml ^NS^ IL-4 Post-ex: Pla: ↑0.59 pg/ml ^NS^, Int: ↑0.32 pg/ml ^NS^ 1 h post-ex.: Pla: ↓ 0.05 pg/ml ^NS^, Int: ↑0.21 pg/ml ^NS^ IL-10 Post-ex : Pla: ↑ 1.05 pg/ml (*p* < 0.05), Int: ↑1.31 pg/ml^NS^ 1 h post-ex.: Pla: ↑ 0.7 pg/ml ^NS^, Int: ↑ 1.3 pg/ml (*p* < 0.05) TNF-α Post-ex: Pla: ↑0.99 pg/ml (*p* < 0.05), Int: ↑1.2 pg/ml (*p* < 0.05) 1 h post-ex: Pla: ↑ 1.1 pg/ml (*p* < 0.05), Int: ↑ 0.2 pg/ml ^NS^
Vaisberg et al. (89)	*n* = 42 RCT	Probiotic vs. placebo	*Lactobacillus casei Shirota, 3*0 days	Marathon race Ambient conditions not stated	IL-1β: Int ↑↓1.8 pg/mL^NS^, Pla ↑13.5 pg/mL ^NS^ IL-1ra: Int ↑18.7 pg/mL^NS^, Pla ↑ 24.7 pg/mL *p* < 0.01 IL-4: Int ↑1.4 pg/mL^NS^, Pla ↑ 6.6 pg/mL ^NS^ IL-5: Int ↑3.1 pg/mL^NS^, Pla ↓ 34.5 ↑ 31.8 pg/mL ^NS^ IL-6: Int ↑ 34.8 pg/mL*p* < 0.01, Pla ↑ 36.5 pg/mL *p* < 0.001 IL-10: Int ↑ 15.0 pg/mL *p* < 0.01, Pla ↑ 19.0 pg/mL *p* < 0.01 IL-12p70: Int ↑ 19.2 pg/mL *p* < 0.05, Pla ↑ 23.0 pg/mL *p* < 0.01 IL-13: Int ↑ 2.1 pg/mL^NS^, Pla ↑ 1.2 pg/mL ^NS^ TNF-α: Int ↑ 20.0 pg/mL^NS^, Pla ↑ 123.4 pg/mL*p* < 0.05 Pla > Int, *p* < 0.01 between groups
West et al. (90)	*n* = 88 RCT	Probiotic vs. placebo	*Lactobacillus fermentum* VRI-003 PCC^®^, 11 wk	Incremental cycling ergometer test (VO_2max_ protocol). Ambient conditions not stated	*Factor changes in acute post-exercise cytokine responses:* IL-1ra: Male: Int 0.84, Pla 1.39 (very likely ↓) Female: Int 0.80, Pla 1.88 (very likely ↓)
					IL-10: Male: Int 0.95, Pla 1.16 (possible ↓) Female: Int 0.89, Pla 1.45 (possible ↓) IL-6: Male: Int 0.92, Pla 1.22 (likely ↓) Female: Int 0.71, Pla 2.29 (likely ↓) IL-8: Male: Int 0.80, Pla 0.87 (unclear) Female: Int 0.71, Pla 1.15 (probably ↓) GM-CSF: Male: Int 0.78, Pla 1.75 (very likely ↓) Female: Int 0.85, Pla 3.3 (very likely ↓) IFN-γ: Male: Int 1.2, Pla 1.49 (likely ↓) Female: Int 1.07, Pla 1.56 (likely ↓) TNF-α: Male: Int 1.27, Pla 1.66 (likely ↓) Female: Int 1.15, Pla 1.72 (likely ↓)
**Short chain fatty acids**					
Valle et al. (96)	*n* = 65 RCT	Synbiotic vs. Placebo	*Lactobacillus acidophilus LA-5, Bifidobacterium animalis BB-12, Inulin 2.3 g* 60 g serve of ice cream, daily for 30 days	5 d military training, including physical and psychological exhaustion, marching 8–20 km, carrying ~ 30 kg equipment, sleep deprivation. Subject to various weather extremes.	Fecal acetate (mmol/L): Pla: Pre: 3·07 ± 1·64, Post: OR 0·16, 95%CI −0·25, 0·57 Post-training: OR −0·71, 95%CI−1·08, −0·34 Int: Pre: 2·82 ± 1·78 Post: OR 0·34, 95%CI −0·06, 0·74 Post-training: OR −0·80, 95%CI −1·14, −0·46 Main effect of time, *P* < 0·001 ^NS^ between groups Fecal proprionate (mmol/L): Pla: Pre: 0·97 ± 0·61, Post: OR 0·31, 95%CI −0·02, 0·63 Post-training: OR−0·08, 95%CI −0·24, 0·08 Int: Pre: 0·83 ± 0·50 Post: OR 0·20, 95%CI −0·01, 0·41 Post-training: OR −0·12, 95%CI −0·26, 0·02 Main effect of time, *P* = 0·004 ^NS^ between groups Fecal butyrate (mmol/L): Pla: Pre: 1·18 ± 0·85 Post: OR 0·25, 95%CI −0·03, 0·47 Post-training: OR −0·09, 95%CI −0·28, 0·10 Int: Pre: 1·04 ± 0·73 Post: OR 0·39, 95%CI 0·20, 0·59 Post-training: OR −0·17, 95%CI −0·33 −0·01 Main effect of time, *P* = 0·002 ^NS^ between groups
**Bacterial taxa**					
Valle et al. (96)	*n* = 65 RCT	Synbiotic vs. Placebo	*Lactobacillus acidophilus LA-5, Bifidobacterium animalis BB-12*, Inulin 2.3 g 60 g serve of ice cream, daily for 30 days	5 d military training, including physical and psychological exhaustion, marching 8–20 km, carrying ~ 30 kg equipment, sleep deprivation. Subject to various weather extremes.	16S gene sequencing *α - Diversity (Shannon index):* *Int:*↑ 0.119 *Pla:* ↓ 0.095 *^*NS*^* *α - Diversity (Simpson index):* *Int:* ↑ 0.015*, Pla:* ↓ 0.021 *^*NS*^* ^NS^ group by time effect ^NS^ α-diversity between groups or periods
**Gastrointestinal symptoms**					
Kekkonen et al. (99)	*n* = 119 RCT	Probiotic vs. placebo	*L. rhamnosus* GG, 3 months	Helsinki Marathon Finish time: Int: 3 h 32 min (range 2 h 24 min to 4 h 35 min) Pla: 3 h 30 min (range 2 h 52 min to 4 h 19 min) ^NS^ between groups Ambient conditions not specified.	During training period: Subjects with GIS episodes: Int 27%, Pla 30% ^NS^ No. of GIS episodes/subject: Int 0.4, Pla 0.6 ^NS^ GIS episode duration: Int 2.9 days, Pla 4.2 days ^NS^ During 2 wk after marathon: Subjects with GIS episodes: Int: 6%, Pla: 6% ^NS^ No. of GIS episodes/subject: Int 0.1, Pla 0.1 ^NS^ GIS episode duration: Int 1.0 days, Pla 2.3 days (*p* < 0.05)
Pugh et al. (79)	*n* = 24 RCT	Probiotic vs. placebo	*L. acidophilus* CUL60 *L. acidophilus* CUL21 *B. bifidum* CUL20 *B. animalis* subsp. *Lactis* CUL34, 4 wk	Non-sanctioned marathon (outdoor running track). Finish time: Int: 234 ± 38 min, Pla: 247 ± 47 ^NS^ % LT: Int: 90.2 ± 9.1, Pla: 91.3 ± 8.7 ^NS^ T_amb_: 16–17°C; Wind: 8–16 km/h	Global GIS score during marathon (median): 1st third: Int 1.3, Pla 1.6 ^NS^ 2nd third: Int 3.0, Pla 3.2 ^NS^ 3rd third: Int 3.5, Pla 6.1 (*p* = 0.01) GIS Score Post-Race (median): Total GIS: Int: 13, Pla 15 ^NS^ Lower GIS: Int 10, Pla 7 ^NS^ Upper GIS: Int 6, Pla 5 ^NS^ GIS Score 24 h Post-Race (median): Total GIS: Int 16, Pla 12 ^NS^ Lower GIS: Int 7, Pla 5 ^NS^ Upper GIS: Int 6, Pla 4 ^NS^
Pugh et al. (100)	*n* = 7 RXT	Probiotic vs. placebo	*L. acidophilus* CUL60 *L. acidophilus* CUL21 *B. bifidum* CUL20 *B. animalis* subsp. *Lactis* CUL34, 4 wk	Cycle ergometer: 2 h at 55% W_max_ followed by 100 kJ time trial. Ambient conditions not stated.	^NS^ between. trials, data not reported
Schreiber et al. (82)	*n* = 27 RCT	Probiotic vs. placebo	l. helveticus Lafti L10, b. animalis ssp. lactis Lafti B94, e. faecium R0026, b. longum R0175, Bacillus subtilis R0179, 90 days	Evaluation (online survey) at training, competition, and during the first 2 h recovery from training or competition.	GIS incidence by slider questionnaire: GIS incidence during training (ΔGI): Int: −27 ± 47%, Pla: 8 ± 29%, Int < Pla, *P* = 0.04, *d* = 0.9 GIS incidence during competition (ΔGI): Int: 0 ± 47%, Pla: 9 ± 30%, ^NS^ GIS incidence after training (ΔGI): Int: −10 ± 32%, Pla: 9 ± 54%, ^NS^ GIS incidence after competition (ΔGI): Int: −20 ± 42%, Pla: 9 ± 54%, ^NS^
Shing et al. (102)	*n* = 10 RXT	Probiotic vs. placebo	*L. acidophilus, L. rhamnosus, L. casei, L. plantarum, L. fermentum, B. lactis, B. breve, B. bifidum, S. thermophilus*, 4 wk	Treadmill running: time to fatigue at 80% ventilatory threshold. Tamb: 35°C, RH: 40%	GIS Symptom Severity Score: Int: 1.4, Pla 1.6 ^NS^
Valle et al. (96)	*n* = 65 RCT	Synbiotic vs. Placebo	*Lactobacillus acidophilus LA-5, Bifidobacterium animalis BB-12*, Inulin, 2.3 g, 30 days	5 d military training, including physical and psychological exhaustion, marching 8–20 km, carrying ~ 30 kg equipment, sleep deprivation. Subject to various weather extremes.	GIS Sum of different symptoms: Int: pre: 8.06 ± 5.65 Δ Post-supp.: OR −2.24, 95%CI −3.15, −1.34 Δ Post-military training: OR −4.31, 95%CI −5.31, −3.30 *P* < 0.05, Δ post-supplementation v. Δ post-military training. Pla: pre: 8.48 ± 5.09 Δ Post-supp.: OR −1.16, 95%CI −2.51, 0.18 Δ Post-military training: OR −3.91, 95%CI −5.01, −2.82 *P* < 0.05, Δ post-supplementation v. Δ post-military training ^NS^ Group × time effect “Both groups showed a decreased number of gastrointestinal symptoms at post-military training (*P* < 0·001; main effect of time) with no differences between them (*P* = 0·37; group × time effect)”

Int, Intervention; Pla, Placebo; RCT, Randomized control trial; RXT, Randomized crossover trial; I-FABP, Intestinal fatty acid binding protein; NS, No significant difference between groups/trials; LPS, Lipopolysaccharide; wk, week.

### Intestinal permeability in response to acute exercise

*N* = 4 probiotic (63, 79, 100, 102) and *n* = 1 synbiotic study (94) assessed the effects of supplementation on intestinal permeability in response to an acute exercise bout (Tables 5, 6). None of the studies observed differences between intervention and placebo for urinary lactulose:mannitol or lactulose:rhamnose ratio, indicative of small intestinal permeability. *N* = 1 study assessed sucrose permeability, indicative of gastroduodenal permeability, at baseline and immediately post-exercise, reporting a 38% significantly lower incremental area under the curve from baseline in the intervention trial, and a 169% increase from baseline in the placebo trial, after 4 weeks *Lactobacillus salivarius* UCC118 (2 × 10^8^ CFU daily) (63). The effect of a synbiotic supplement (four probiotic strains plus fructooligosaccharides) was investigated on urinary lactulose:mannitol ratio, before and 6 days after a long course triathlon event, with no effect of trial observed (94).

### Systemic bacterial endotoxin profile in response to acute exercise

*N* = 2 probiotic studies (68, 100) and *n* = 1 synbiotic study (94) investigated changes in circulating bacterial endotoxin concentration in response to acute exercise (Tables 5, 6). Biomarkers included overall endotoxin units, gram-negative endotoxin concentration, serum LPS, anti-endotoxin antibodies (i.e., IgM and IgG), and neutrophil elastase (*in vitro E. coli* LPS stimulation). No supplement intervention reduced markers of endotoxemia compared with the study's respective placebo, whilst one intervention (7 days *Lactobacillus casei*) reported an increased gram-negative bacterial endotoxin concentration in response to 2 h steady-state treadmill running (60% *V*O_2max_) in hot ambient conditions (34.0°C, 32% RH), compared to a modest reduction in the placebo group (68). *N* = 1 study reported a significant reduction in endotoxin units compared to pre-supplementation (94); however this data was compared to the pre-exercise and not the pre-supplementation time point (i.e., a sample taken the day before an ultra-distance triathlon event), then assessed 6 days post-race.

### Systemic inflammatory cytokine profile in response to acute exercise

*N* = 10 studies assessed systemic inflammatory cytokine responses to acute exercise, all of which utilized probiotic supplementation interventions (63, 65, 68, 76, 79, 87, 89, 90, 100, 102) (Tables 5, 6). Only *n* = 3 studies observed differences in the cytokine response to exercise between probiotic and placebo trials (87, 89, 90). One study reported statistical analysis using magnitude-based inferences, suggesting probiotic supplementation (11 weeks *Lactobacillus fermentum* VRI-003 PCC^®^, 10^9^ CFU/day) resulted in possible and very likely reductions in IL-10 and IL-1ra, respectively, a likely reduction in IL-6, and likely or very likely reductions in GM-CSF, IFN-γ, and TNF-α, respectively (90). In contrast with this, a significant increase in IL-10 was observed only in the probiotic group at 1 h post-exercise, and TNF-α increased significantly only in the placebo group at 1 h post-exercise compared with 24 h pre-exercise levels, following 30 days of supplementing with *Lactobacillus acidophilus-LB-G80, Lactobacillus paracasei-LPc-G110, Lactococcus subp. lactis-LLL-G25, Bifidobacterium animalis subp. lactis-BL-G101* and *Bifidobacterium bifidum-BB-G90* (87). Following 30 days of supplementation with *L. casei Shirota*, a significant rise in IL-1ra was observed on the intervention group only (within group difference) but the only significant between group difference was observed in TNF-α, whereby a significant increase in TNF-α only occurred in the placebo group and not the intervention group (89).

### Gastrointestinal functional markers in response to acute exercise

None of the included studies assessed the effect of pre-, pro-, syn-biotic supplementation on aspects of gastrointestinal function (e.g., gastric emptying, gastrointestinal motility, intestinal transit, intestinal nutrient absorption, and/or malabsorption) in response to acute exercise.

### Gastrointestinal symptoms in response to acute exercise

*N* = 5 studies, of which *n* = 4 were probiotic and *n* = 1 synbiotic supplementation interventions, included an assessment of GIS during the acute exercise bout (79, 82, 96, 100, 102). Of the probiotic interventions, *n* = 2 reported no effect of supplementation on GIS during exercise (100, 102). *N* = 1 study reported no difference in median global GIS score (GIS severity) during the first two-thirds of a simulated marathon. However, the authors emphasized a greater score in the placebo group during the final third of the simulated marathon, although this discrepancy appears likely due to lack of experimental control to confounding factors (refer to risk of bias assessment) (79). Additional data presented shows slightly higher overall GIS incidence in the probiotic group (90%) compared with the placebo group (88%) in response to the simulated marathon, and median GIS score immediately post-race was not different between groups. *N* = 1 study using online surveys to report GIS incidence by participants during training, reported a greater improvement in symptoms in the probiotic group, although GIS incidence after training, and during and after competition, showed no changes following probiotic supplementation (82). Similarly, 3 months of supplementation with a probiotic showed no significant effects on GIS incidence or duration in training, however during 2 weeks after the marathon, the category of GIS episode duration was more than double the number of days in the placebo group than the probiotic group (99). The *n* = 1 synbiotic study reporting GIS during an exercise bout (5 days continuous military training exercise) following supplementation showed no difference between groups, but an effect of time was observed, whereby symptoms reduced in both groups following the military training exercise bout (96).

### Fecal bacterial taxa changes and short chain fatty acid concentration in response to acute exercise

Only *n* = 1 included studies assessed changes on bacterial taxa and fecal SCFA concentration in response to acute exercise (96) (Tables 5, 6). Following 30 days of supplementation with a synbiotic containing *L. acidophilus LA-5, B. animalis BB-12*, and 2.3 g of inulin, military recruits participated in 5 days of continuous combat simulation. No difference in fecal acetate, propionate or butyrate was observed. Some changes in bacterial taxa were noted in text, however due to the method of presentation in the manuscript (i.e., heat map), logical conclusions could not be drawn. Measures of bacterial diversity (Shannon and Simpson Index) before and after the exercise bout showed no difference in α-diversity following synbiotic supplementation compared with placebo, and no group by time effect was observed.

### Risk of bias assessment

Results of the risk of bias assessment appear in Table 7. *N* = 18 out of the *n* = 39 included studies were judged as high risk of bias in at least one criterion. This included *n* = 2 due to a sequenced allocation as part of a counterbalanced randomization (59, 88), *n* = 10 due to inadequate reporting of outcome assessor blinding (72–74, 77, 78, 80, 82, 84, 87, 93), *n* = 5 due to incomplete outcome reporting (74, 78, 87, 100, 102), and *n* = 4 due to selective data reporting (78, 79, 84, 98). Other potential sources of bias were identified in *n* = 5 studies (79, 84, 88, 94, 102). *N* = 1 study reported increased GIS in the placebo group during the final third of a non-sanctioned marathon, however closer inspection of the data suggested that relative incidence (i.e., 91 vs. 89% of total group, respectively) and severity (i.e., 63 vs. 44% of total group above the mean of the assigned global GIS score, respectively) of GIS on the 4-weeks probiotic supplementation group was greater than the placebo group throughout the simulated marathon. Furthermore, the severity of GIS findings may have also been confounded due to large differences in total fluid volume intake between groups (e.g., varied completion times and total intake volumes (i.e., carbohydrate gel and water) that were not systematically assessed, discrepancies in reported plasma volume changes between groups, the absence of validated hydration status or change markers and body mass data), rather than any effect of the intervention itself (79), as highlighted in Costa et al. (19), Costa et al. (46), and Hoffman et al. (106). The remaining studies failed to provide evidence of correction of blood-based biomarkers for changes in plasma volume (107) as would be expected to occur in the exercise and/or heat stress models used (63, 65, 87, 89, 90, 94, 102). More than half of the included studies were either directly funded by, had intervention supplementation and/or placebo substances supplied by, or were authored by employees or paid consultants of, the manufacturer of the pre-, pro-, or syn-biotic product studied (56, 57, 62, 63, 70, 76, 77, 79, 82, 85, 88–90, 92–94, 99, 100, 102).

**Table 7:** Risk of bias assessment.

**References**	**Sequence generation**	**Allocation concealment**	**Participant/** **personnel**	**Outcome assessment blinding**	**Incomplete outcome data**	**Selective reporting**	**Other potential sources of bias**
Axelrod et al. (63)	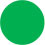	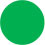	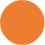	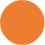	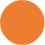	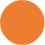	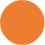
Batatinha et al. (65)	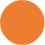	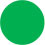	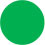	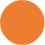	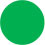	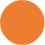	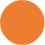
Burton et al. (66)	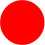	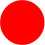	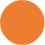	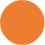	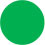	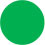	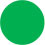
Carbuhn et al. (67)	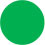	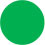	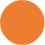	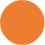	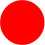	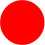	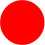
Coman et al. (92)	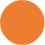	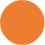	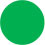	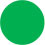	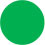	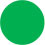	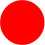
Damen et al. (55)	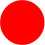	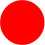	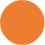	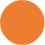	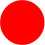	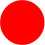	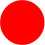
Finegold et al. (56)	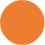	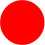	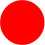	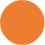	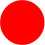	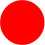	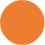
François et al. (57)	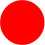	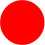	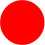	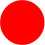	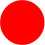	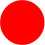	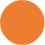
Gill et al. (68)	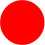	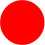	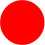	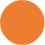	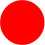	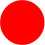	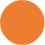
Gleeson et al. (70)	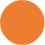	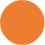	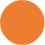	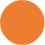	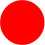	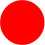	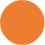
Haywood et al. (72)	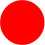	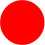	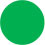	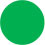	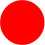	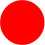	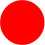
Huang et al. (74)	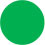	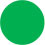	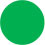	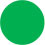	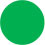	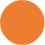	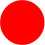
Hoffman et al. (73)	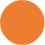	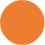	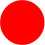	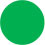	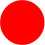	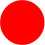	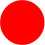
Kekkonen et al. (99)	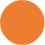	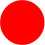	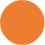	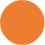	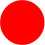	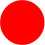	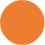
Kleessen et al. (59)	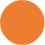	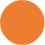	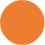	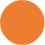	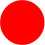	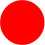	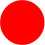
Klein et al. (75)	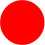	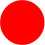	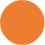	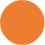	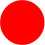	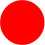	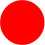
Lamprecht et al. (76)	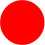	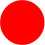	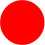	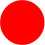	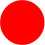	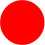	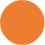
Lee et al. (77)	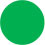	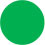	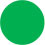	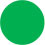	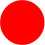	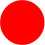	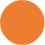
Lin et al. (78)	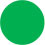	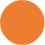	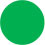	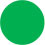	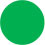	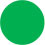	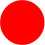
Pugh et al. (79)	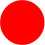	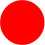	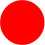	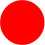	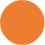	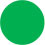	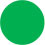
Pugh et al. (100)	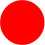	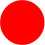	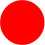	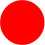	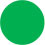	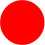	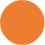
Quero et al. (93)	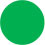	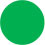	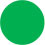	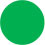	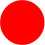	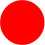	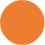
Reimer et al. (60)	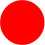	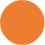	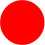	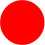	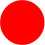	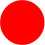	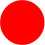
Roberts et al. (94)	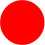	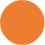	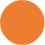	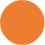	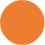	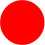	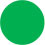
Russo et al. (61)	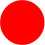	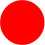	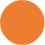	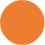	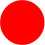	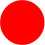	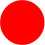
Russo et al. (45)	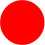	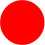	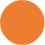	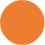	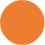	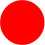	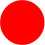
Russo et al. (62)	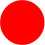	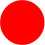	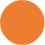	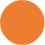	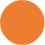	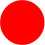	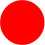
Sánchez Macarro et al. (80)	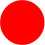	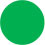	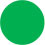	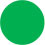	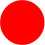	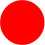	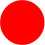
Schreiber et al. (82)	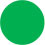	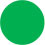	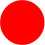	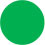	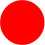	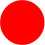	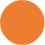
Shing et al. (102)	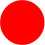	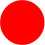	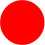	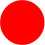	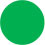	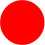	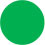
Smarkusz-Zarzecka et al. (83)	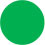	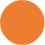	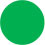	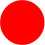	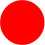	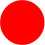	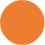
Son et al. (84)	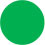	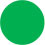	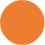	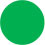	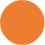	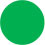	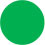
Strasser et al. (85)	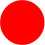	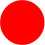	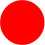	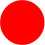	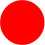	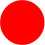	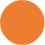
Tavares-Silva et al. (87)	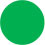	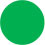	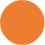	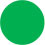	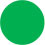	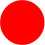	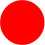
Townsend et al. (88)	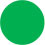	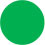	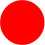	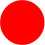	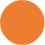	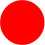	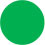
Vaisberg et al. (89)	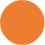	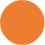	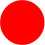	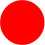	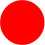	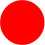	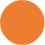
Valle et al. (96)	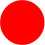	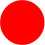	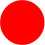	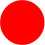	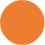	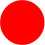	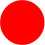
West et al. (90)	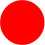	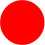	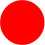	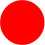	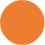	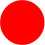	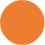
West et al. (98)	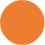	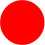	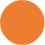	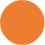	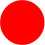	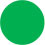	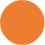

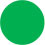
 Low risk of bias, 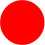 High risk of bias, 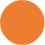 Unclear risk of bias.

## Discussion

The aim of this systematic literature review was to determine the beneficial, detrimental, or neutral effects of differing supplementation periods and dosages of pre-, pro- and syn-biotic supplementation, taken by healthy active adults, on gastrointestinal outcomes at rest and in response to exercise, with a specific focus on markers characteristic of EIGS and associated GIS. At rest, positive outcomes have been reported on measures of reduced intestinal permeability in 2/6 studies (*n* = 1 pre- and *n* = 1 pro-biotic interventions), improvements in functional measures in 2/4 prebiotic studies, improvements in gastrointestinal symptoms in 2/7 probiotic studies, and improvements in resting systemic cytokines in 3/15 studies. No changes were detected in all other studies assessing these measures at rest. In response to exercise, where the gastrointestinal tract is acutely perturbed (2, 3, 108), the effects were even more modest with 0/3 studies showing a reduction in intestinal injury following probiotic supplementation. Only 1/5 studies showing a significant reduction in measures of intestinal permeability, 1/3 studies suggested an increase in systemic bacterial endotoxin profile, and only 3/10 studies suggested an effect of supplementation on systemic inflammatory cytokine profile in response to exercise. Improvements in selectively reported measures of GIS in response to exercise following probiotic supplementation were reported in 2/5 studies. It is important to highlight the magnitude of exercise-associated gastrointestinal disturbances and differences between intervention and placebo groups in studies reporting positive effects of supplementation interventions, are modest in nature and study conclusions suggesting beneficial effects of supplementation (i.e., lower intestinal permeabiluty, endotoxaemia, cytokine responses) are to be interpreted with caution given methodological issues and concerns identified, as recently discussed in Costa et al. (44). Only a limited number of studies have assessed GIS during exercise, with either minimal or no effect of probiotic supplementation observed, and with likely distorted outcomes associated with a lack of control of established confounding factors (79). The effect of prebiotics on gastrointestinal outcomes during exercise have not yet been studied, preventing any conclusions being drawn. The data synthesized in this review suggest pre-, pro-, and syn-biotic supplementation exerts inconsistent effects on gastrointestinal integrity, function, symptoms and resultant systemic response, at rest. In response to exertional or exertional heat stress, no consistent and substantial beneficial effects are seen with probiotics or synbiotics on gastrointestinal status.

### Pre-, pro-, and syn-biotics and markers of gastrointestinal integrity

The role of intestinal barrier integrity, in both adequate nutrient absorption and in preventing unwanted translocation of bacterial endotoxins into circulation, is seen as one key factor influencing the likelihood of EIGS (2). There is now substantial evidence that exercise-associated epithelial enterocyte injury, measured through the surrogate marker I-FABP, is accompanied by an increase in systemic bacterial endotoxin from luminal origin, and subsequent systemic inflammatory responses (109, 110), similar to those values observed in clinical populations (e.g., medical complications of the gastrointestinal tract) (2, 19, 34, 111–118). These gastrointestinal integrity outcomes are relatively asymptomatic during exercise, but may instigate GIS in the post-exercise recovery period (e.g., abdominal pain, osmotic diarrhea, urge to regurgitate, regurgitation, and/or fecal blood loss), as a result of acute reversible colitis (2, 3). Such perturbations to gastrointestinal integrity (e.g., plasma I-FABP concentration: Δ pre- to post-exercise ≥1,000 pg/ml) are consistently seen with exercise stress loads ≥2 h of endurance exercise at 60% *V*O_2max_ in hot ambient conditions (≥35.0°C) where peak core temperature reaches ≥39.0°C, irrespective of relative humidity (33, 35, 109, 110, 119), or with ≥3 h of endurance exercise at 60% *V*O_2max_ in temperate conditions (~20°C) with minimal rise in core body temperature (19, 34). Any lesser exertional or exertional-heat stress appears to result in no or minimal perturbations to gastrointestinal integrity, or perturbations of no clinical relevance. It is therefore unsurprising that the studies included in this review, almost universally failed to substantially influence aspects of intestinal integrity, endotoxemia or cytokinemia at rest, given that these mechanisms are unlikely to occur to any significant extent in the absence of a medical gastrointestinal condition, or a bout of substantial exercise stress. However, this may be in part due to the insufficient exercise or heat stress required to significantly perturb the intestinal barrier in most studies. In addition, one study reported no pre- to post-exercise increase in plasma I-FABP concentration in the probiotic or placebo groups as a result 2 h cycling at 55% W_max_ followed by a time trial in which a carbohydrate beverage was provided throughout the exercise bout (100). The authors purported this outcome was due to insufficient exercise stress load, but it is also likely that carbohydrate consumption during the exercise protocol was able to completely ameliorate exercise-associated epithelial injury [i.e., abolished plasma I-FABP response, as reported by Snipe et al. (38)], as observed during other exercise carbohydrate feeding studies (103, 120–123). This effect has been attributed to carbohydrate absorption-associated, nitric oxide-induced, villi microvascular dilation and perfusion (37, 103, 123). Together the data presented in this systematic review provides no evidence that probiotics exert an effect on gastrointestinal integrity, and to date no studies of synbiotics or prebiotics have investigated this aspect of EIGS.

### Pre-, pro-, and syn-biotics and markers of gastrointestinal permeability

Exercise-associated modulation to intestinal epithelial injury and intestinal epithelial permeability, and their respective biomarkers (i.e., direct or indirect surrogate biomarkers) are not the same, and outcome data for these cannot be used interchangeably, as discussed in a recent study by Gaskell et al. (34). Several recent studies have observed a mismatch between exercise-associated changes to plasma I-FABP concentration (i.e., epithelial enterocyte injury) and lactulose:mannitol or lactulose:rhamnose dual sugar test (i.e., intestinal epithelial tight-junction permeability), and/or plasma or fecal claudin-3 concentration that is a proposed surrogate marker for epithelial tight-junction damage (35, 109, 124, 125). Indeed, the magnitude of intestinal epithelial injury and permeability differs in response to the same exertional or exertional-heat stress (38, 109, 124, 125), with permeability measures not increasing in proportion to exercise stress, and not leading to post-permeability outcomes (i.e., increased plasma endotoxin, anti-endotoxin, and inflammatory cytokine concentrations) (2, 3). Considering increased intestinal permeability in response to exercise stress does not correlate with epithelial injury, systemic endotoxin and inflammatory cytokine profiles, and GIS; in studies that have included a global gastrointestinal assessment (35, 38, 109, 125), it appears increases in intestinal permeability is a habitual response to exercise stress, with a set threshold, and of little relevance to the key health outcomes of EIGS (e.g., aggressive acute or repetitive strain epithelial injury, systemic endotoxemia and inflammatory cytokinemia, and/or gastroparesis with or without paralytic ileus). Regardless, the studies included in this systematic literature review did not provide any substantial evidence, at rest or in response to exercise, that pre-, pro- or syn-biotic supplementation could reduce intestinal permeability. *N* = 1 study that reported improvements in permeability at rest following 14 weeks multi-strain probiotic supplementation (63) should be interpreted with caution given known limitations, including analysis procedures now identified as poor indicators of intestinal permeability (e.g., fecal or plasma zonulin concentration determination) (126, 127). Only *n* = 1 prebiotic study assessed permeability at rest, concluding an improvement in urinary lactulose:mannitol ratio following 5 weeks of consumption of inulin enriched pasta, compared with placebo (62). In response to exercise, *n* = 1 study assessed intestinal permeability 6-days after completion of the exercise stress (i.e., long course triathlon event) (94). Given the transient nature of exercise-induced changes in gastrointestinal permeability, it is not surprising that this study did not observe any substantial differences from pre-exercise values (i.e., sample time point was 1 day prior to the event). Despite one included study measuring claudin-3 in response to exercise, fecal sampling for biomarker determination was the included method (102). Considering gastrointestinal integrity perturbations of EIGS are transient in nature, as opposed to the consistent perturbation seen in inflammatory diseases of the gastrointestinal tract (e.g., Crohn's disease and ulcerative colitis), it is now well established that measuring fecal biomarkers to determine the extent of gastrointestinal permeability, with or without adjoining injury and inflammation biomarkers, risks erroneous interpretations due to issues surrounding sample collection timing, volume, and processing methods (44).

### Pre-, pro-, and syn-biotics and systemic endotoxin response

Since intestinal integrity is not compromised at rest in otherwise healthy individuals, endotoxemia is unlikely to occur to any significant extent (2, 3). Consistent with this, none of the *n* = 5 studies reporting resting endotoxin concentration showed any changes following supplementation with a pro- or syn-biotic. In response to an acute exercise bout, since intestinal injury was not significantly impacted, subsequent systemic endotoxin remained unaffected to any substantial degree by the supplementation with the pro- or syn-biotics studied (94, 102). In contrast, *n* = 1 study showed an increase in gram-negative bacterial endotoxin concentration during the recovery period of exertional-heat stress, as a result of *L. casei* supplementation (68). Thus, the evidence to date suggests that supplementation with pro- or syn-biotics show no benefit to endotoxin response at rest or following exercise stress.

### Pre-, pro-, and syn-biotics and cytokine response

Cytokine responses, which are consistently reported as the key pathophysiological endpoint for clinical significance (i.e., negative health affects), were largely unaffected by the majority of studied supplements. Where positive effects on cytokines following probiotic supplementation compared with placebo were observed, no consistent pattern was seen across the cytokine profile studied, but rather isolated changes were observed, such as; an attenuation in the rise in inflammatory C-C Motif Chemokine Ligand 2 (CCL2) following 2 weeks supplementation with *S. thermophilus, L. delbrueckii* spp. *Bulgaricus*, and *L. rhamnosus* GG, compared with placebo (66) but no change in other inflammatory cytokines observed (TNF-α, CCL5, IL-6); and an attenuated rise in tumor necrosis factor alpha (TNF-α) following 12 weeks supplementation with *B. subtilis*, compared with placebo, but no change in anti-inflammatory interleukin IL-10 (88). Furthermore, inconsistent results were shown following 30 days of supplementation with a multi-strain probiotic, whereby a greater reduction in pro-inflammatory interleukin IL-2, an attenuated reduction in anti-inflammatory IL-4, and a greater reduction in anti-inflammatory IL-10 was observed, compared with placebo (87). Multi-strain synbiotic supplementation also showed inconsistent results, namely a 50% lower circulating IL-16 concentration, compared to a prebiotic control (i.e., acacia gum) with no difference observed in Il-18, while IL-12 and IFN-γ were undetectable in assay (62). Another study with a multi-strain synbiotic for 30 days reported greater reduction in circulating IL-10 concentration in the placebo group than those on the intervention (93). In response to exercise, only two studies showed improvements and, in some cases, contradictory findings in cytokine response following exercise. Eleven weeks of supplementation with *Lactobacillus fermentum* VRI-003 PCC^®^, resulted in lower pre- to post-exercise increases in plasma IL-1ra, IL-6, IL-8, IL-10, GM-CSF, IFN-γ, and TNF-α concentrations (90) using magnitude based inferences, whereas a significant increase in IL-10 was observed 1 h post-exercise, following 30 days of supplementing with a multi-strain probiotic, and TNF-α increased only in the placebo group at 1 h post-exercise compared with 24 h pre-exercise levels (87). However, most important to note is that the magnitude of systemic cytokine responses in these studies were minimal in comparison to more aggressive exercise models and ultra-endurance field events (16, 69, 109), and are unlikely to be of clinical relevance. It therefore appears that there is no compelling evidence that probiotics or synbiotics exert any clinically relevant effect on resting cytokines and perhaps less so on cytokine responses to exercise at the intensities and exercise volumes observed. Whether these biotic interventions could show an attenuated systemic inflammatory effect at exercise interventions causing more activation of the immune system, as reported in Costa et al. (44), remains unknown.

### Pre-, pro-, and syn-biotics and markers of gastrointestinal function

Gastrointestinal functional responses are an important component of EIGS, and give rise to many of the unpleasant GIS experienced by active adults, both at rest and during exercise (128). Functional responses include measures of gastrointestinal motility and transit, such as gastric emptying rate, EGG, OCTT, defecation frequency, and stool consistency (45, 55, 59, 61). Other functional responses include magnitude of malabsorption to a nutrient challenge and subsequent bacterial fermentation of intestinal residue, typically assessed through breath hydrogen and/or methane responses (6, 38, 46, 129). It was somewhat surprising that only 4/39 included studies reported data pertaining to functional responses, and none in response to exercise. Of the measures included, only gastric emptying rate was reduced by the consumption of prebiotic inulin enriched pasta, compared with a placebo meal (61), overall gastrointestinal motility and function appears minimally affected, and only *n* = 1 study observed a beneficial reduction in GIS, with other studies suggesting a possible increase, which is consistent with bacterial fermentation of poorly absorbed nutrient/s increasing luminal content and pressure. Whilst this data was captured at rest, it appears consistent with a recent study, not included in this review's inclusion criteria, showing that a 24 h high FODMAP diet (46.9 g/day) and high FODMAP pre-exercise meal (26.2 g), both which contained a substantial fructan component (10.1 and 1.4 g, respectively) that is consistent with prebiotic supplementation doses, increased upper-GIS severity at rest and in response to exercise in a healthy active population compared to a 24 h low FODMAP diet (<5 g/day) (35). The authors speculated that reduced gastric motility was the likely mechanistic cause of such findings, as FODMAPs pass through the small intestine as residue, are readily fermentable by commensal bacteria, and the residue and fermentation contribute to increase intestinal lumen content and pressure. These outcomes are likely to activate the gastrointestinal braking mechanism that reduces gastric emptying rate and intestinal transit (6–10).

### Pre-, pro-, and syn-biotics and gastrointestinal symptoms

Of all the outcomes presented in this systematic review, the one of most interest and relevance to active adults is the experience of GIS, given this is likely one of the main reasons consumers would choose to consume a pre-, pro-, or syn-biotic product, and the factor that has performance implications (i.e., GIS directly linked to reduced distance test performance; and workload reduction, cessation or withdrawal from exercise activity) (19, 130). At rest, the majority of studies saw minimal GIS incidence and severity, and therefore minimal differences between supplement intervention and placebo. Of those that did show statistically significant differences in GIS, most were of low incidence and/or severity, or categories of symptoms were selectively reported, to the exclusion of others where no change in overall symptoms were observed (83); and in some cases, GIS were greater during consumption of the supplement intervention compared to placebo. Due to the potential health and performance debilitating effect of EIGS and associated GIS in active populations, there is substantial interest in manipulating factors that may reduce the incidence and/or severity of EIGS and associated symptoms. Using the EIGS model (Figure 1), it can be seen that interventions designed to reduce either the effect of primary causal mechanisms (i.e., splanchnic blood flow, and the neuroendocrine stress response to exercise), or the secondary outcomes (i.e., intestinal barrier integrity, nutrient absorption capability, and the presence or absence of undigested and/or fermentable residue in the gastrointestinal tract), should theoretically contribute to a reduction in unwanted outcomes. In the case of pre-, pro- and syn-biotics, such interventions are mostly aimed at targeting the secondary mechanisms of the gastrointestinal-circulatory pathway of EIGS, by potentially enhancing the stability and function of individual epithelial cells, and their bonded relationship with adjacent cells within the gastrointestinal epithelial layer. Only *n* = 5 studies were identified that have assessed GIS in response to exercise, none of which provided compelling evidence that probiotics could improve GIS incidence or severity. Moreover, it is important to note that the majority of studies did not use a validated or reliability-checked GIS assessment tool, instead using in-house or Likert-type rating scales, or online questionnaires with unclear origins and symptom types, possibly because GIS was a secondary outcome in many study designs. Validated and reliability-checked GIS assessment tools like the visual analog scales and ROME III criteria for symptom type were not consistently applied (131–133). The only study investigating GIS following synbiotic supplementation, in military recruits engaged in a 5-day continuous military training exercise following supplementation regime, showed no difference between groups, pre- to post-supplementation, or following the multi-day training exercise, but an effect of time was observed, whereby symptoms reduced in both groups following the military training exercise bout (96). This suggests that military training is more effective at reducing symptoms than the synbiotic supplement provided at rest. Currently there are no published studies that have assessed the impact of prebiotic supplementation on GIS during exercise, warranting further research.

### Pre-, pro-, and syn-biotics and gut microbial composition and short chain fatty acids

The interaction between the “*gut microbiota*” and human biological systems has gained much research interest and translational application traction. The role of commensal and pathogenic bacteria, and their metabolic by-products (e.g., SCFA) and structural residues (e.g., endotoxins) are increasingly being recognized as contributing to the attenuation or exacerbation of pathophysiologic pathways in numerous clinical conditions (e.g., cardiometabolic, mental health, gastrointestinal disease and disorders, and systemic inflammatory conditions) (43). Whilst most of the gastrointestinal mechanistic research has been conducted with *in vitro* or animal models, translation to interventions targeting human gut microbiota are growing rapidly (40–43, 134–136). From the current literature it appears that the beneficial role of the gut microbiota is associated with intestinal commensal bacteria producing SCFA (i.e., butyrate, acetate, and propionate) and other metabolic by-products (e.g., anti-inflammatory factors). The family groups *Lachnospiraceae* and *Ruminoccoccaceae*, and genus *Akkamensia, Bacteroides, Bifidobacterium, Clostridium* (e.g., species *leptum*), *Faecalibacterium, Lactobacillus*, and *Rosburia* are reported to stimulate luminal host immunity *via* intestinal secretion of anti-microbial proteins and activation of innate immune responses, enhance the intestinal epithelial structural barrier (i.e., mucus production, enterocyte cell proliferation, and tight-junction protein expression), reduce pathogenic adhesion to intestinal epithelial apical surface, and improved gastrointestinal motility, including facilitating peristalsis. Conversely, pathogenic bacteria including *Escherichia coli, Salmonella, Shigella*, and (or) *Campylobacter* and their structural residues (e.g., endotoxins- LPS, peptidoglycan, flagellin, lipoteichoic acid, and muramyldipeptide) are potent stimulators of local epithelial and systemic immune responses (*via* Nfκβ and phagocytic immune cell activation), through the TLR-4 activation pathway identifying PAMP on pathogenic bacterial surfaces. Therefore, it appears increased bacterial α-diversity, increased relative abundance of SCFA producing commensal bacteria, and decreased relative abundance of endotoxin-presenting pathogenic bacteria, meets the criteria for optimal “*gut health*” in respect to gut microbiota composition.

Changes in intestinal microbial composition, as determined by fecal bacterial counts as CFU/g feces *via* fluorescence *in situ* hybridization (FISH), traditional cell cultures, or quantitative polymerase chain reaction (qPCR), or determined by fecal bacterial taxa as relative abundance and α-diversity of operational taxonomic units (OTU) *via* more modern sequencing techniques (e.g., 16S or shotgun sequencing), were highly variable in the included studies. The direct comparison between studies is difficult to establish due to differences in the methods of reporting data at different levels of taxonomy, the diverse or limited bacterial types reported in various studies, and the differing reporting units used (i.e., absolute vs. relative values, and reporting bacterial counts per wet vs. dry mass of feces). For example, determination of bacterial composition using FISH and/or bacteria specific qPCR methods, as predominaly used in the older dated studies, provides a value for bacterial counts relative to the total identifiable bacterial counts as per weight of sampled feces (e.g., CFU/g). Whereas, the more recent studies used gene sequencing techniques, which are limited to the relative abundance of the total bacterial count detected (e.g., %). Thus, caution is warranted in interpreting the outcomes obtained in regards to the biotic interventions when comparing studies using different bacterial determination techniques. Furthermore, despite attempts to establish a “*healthy gut microbiota profile*”—or normative composition, as discussed by Bennett et al. (33), there is currently no well-established gut microbiota profile considered as a “healthy athlete” profile. This is likely due to the large individual variability within and between individuals, and from an experimental perspective the heterogeneous experimental designs and lack of confounding factor control (e.g., dietary, exercise, circadian, ambient conditions, etc.) within and between studies (33, 44). Within the current review, and taking these limitations into consideration, the most consistent changes in gut microbial composition came from prebiotic supplementation interventions, with increases in the relative abundance of *Bifidobacterium* reported in all included studies, except one, and no change in the abundance of *Lactobacillus* in any prebiotic study (Table 4). Probiotic and synbiotic supplement interventions appeared to significantly increase the relative abundance of the supplemented strains where measured. However, the effect on the abundance of other microbiota appeared inconsistent and mostly negligible, with the exception of a nine-fold increase in *Lactobacillus* following supplementation with *Bifidobacterium longum subsp. Longum* (78). In all included studies, any changes in selected bacterial taxa did not result in improvements in any measure of bacterial diversity reported (e.g., α-diversity (Shannon index), richness, Simpson index or 16SrRNA gene sequencing).

SCFA have previously been proposed as key by-products of bacteria metabolism that support intestinal epithelial integrity (137, 138). The presence of greater concentrations of SCFA in fecal samples following pre-, pro- or syn-biotic supplementation at rest may indicate a successful increase in the absolute or relative abundance of SCFA-producing bacteria. A clear delineation was made between pre- and pro-biotics with respect to SCFA concentration. AXOS prebiotics are produced as a by-product of the bread-making process by enzymatic reaction with naturally occurring arabinoxylans in grains, allowing bread manufacturers to manipulate prebiotic content of the baked product, without fortification (139). Higher dose AXOS based prebiotic supplemental protocols demonstrated an increase in fecal total SCFA, acetic and butyric acids (7.2 g/day AXOS for 3 weeks) (55) and in one study these changes also included an increase in propionic acid concentrations (8.0 g/day AXOS for 3 weeks) (57). These increases in SCFA were not seen in lower dose prebiotic supplemental protocols (2.4 g/day AXOS) (57), 2.8 g/day XOS (56) or any dose of inulin-based prebiotic. No positive effects in resultant fecal SCFA due to probiotic supplementation were observed. Only one of the included studies investigated fecal SCFA concentrations following synbiotic supplementation, that included an acute exercise component (5 days continuous intense military training exercise) but no change was reported between groups at rest or following the prolonged exercise bout (96). Further research targeting increases in SCFA producing bacteria, utilizing prebiotic ingredients shown to have such an effect, and including an exertional-heat stress component, is required to demonstrate if such supplements can consequently improve intestinal integrity and reduce EIGS outcomes in athletes. It has been noted in one of the included studies that a lack of change in fecal SCFA concentration may not necessarily reflect a lack of change in production, but instead be caused by increased metabolism of SCFA by the host (56), suggesting that any such study should also include measurement of changes in the abundance of SCFA producing bacteria. Indeed, changes in SCFA are best measured in blood, as increases in circulatory SCFA have been noted in the absence of fecal SCFA changes, indicating higher luminal absorption (140).

### Study limitations

The major limitation of this systematic review is the very small number of studies identified that took a comprehensive approach of well-validated biomarkers, in such a way that readers can establish the cause-and-effect relationship between the supplement intervention, and both the mechanisms and outcomes of EIGS in a systematic manner. Indeed, a significant number of included studies consisted of small sample sizes, with *n* = 10 studies consisting of *n* ≤ 15 participants and only *n* = 12 studies consisting of *n* ≥ 30 participants. Sample size determination was either not specified, or reported as underpowdered in *n* = 20/39 included papers. Additionally, the complete absence of studies that have provided prebiotic supplements and investigated the subsequent response to acute exercise, prevents us from drawing conclusions in this area. Whilst the possibility exists that we failed to identify all previous studies related to the research question, this risk was minimized through the use of six academic databases in the literature search. In addition, all recent review papers found during the search were scanned for additional papers, however no further records were identified. The lack of effective dietary control in the vast majority of studies included is a significant limitation to study interpretation, with no dietary control other than instruction of what foods or beverages to avoid in *n* = 15 studies, and habitual diet with the request that participants keep a food-fluid log (e.g., 1–3 days before exercise trial) or record pre-trial intake and attempt to duplicate intake on any subsequent trial/s in a further *n* = 15 studies, many of which only stated non-significant difference without reporting the actual data for energy and macronutrients, including fiber. Only *n* = 8 included studies provided food to participants to control dietary intake prior to measures being taken; one of which dietary control was inherent to the research setting (military barracks food service), with no indication of energy or macronutrient content controlled for. None of the included studies controlled for FODMAP content of the diet, which is a known prebiotic food constituent that is broadly represented in the western diet (141). It is now well established that dietary FODMAP intake leading into exercise (e.g., experimental trials) influences gastrointestinal integrity and functional outcomes, systemic responses, and GIS (35, 44). The absence of a meta-analysis may also be considered a limitation of this review, highlighting the heterogeneity in reporting findings of key gastrointestinal markers. Therefore, in accordance with the data presented in this systematic literature review the impact of pre-, pro- and syn-biotics on gastrointestinal outcomes in healthy and active adults at rest and in response to exercise remains largely negligible, with no substantial effect on markers of gastrointestinal integrity and systemic responses, and minimal and inconsistent effects on function and symptoms.

## Implications for research and practice

As already discussed, the data captured by this review does not provide any convincing evidence for beneficial effects, and/or the methodological issues acknowledged and raised in the included studies does not allow many definitive conclusions to be drawn regarding the impact of pre-, pro-, and syn-biotic supplementation on markers of gastrointestinal status at rest and in response to exercise. Future research would benefit from taking a bottom-up approach, utilizing existing findings in academic literature to build a pathway from supplementation to changes in gut microbiota, to mechanistic changes in the host, and finally to beneficial outcomes (e.g., barrier integrity, function, systemic responses, and symptoms). Given that symptoms are likely a main reason consumers would choose to consume a pre-, pro- or syn-biotic product, future studies should use exercise protocols of sufficient intensity, duration and ambient conditions to adequately provoke GIS, and purposefully recruit athletes with a history of GIS, thus making it more likely to observe improvements following a period of supplementation. Considering the acute and rapid plasticity of the gastrointestinal tract and emerging evidence that pre-exercise dietary intake can influence the magnitude of EIGS and exercise-associated GIS (35, 46), future research in this area should provide participants, and report on, all food and fluid consumed at least 24 h before experimental procedures and throughout the experimental period. Laboratory-based research targeting EIGS management strategies, application and reporting of at least a 24 h low FODMAP, and matching for fiber intake, to meet energy needs that is macronutrient balanced is recommended (34, 35, 38, 109, 110, 125); this may not necessarily apply to exploratory field-based research. Prospective food-fluid intake logs are best used to assess compliance with the control diet provided. Few studies have investigated the effect of synbiotic supplementation on EIGS mechanisms or outcomes, but those that have tended to produce results more closely resembling probiotics than prebiotics. This probably reflects the very small quantities of the included prebiotic, as many synbiotic supplements are consumed in capsule form, preventing larger quantities of prebiotic ingredients from being consumed. Mechanistically it appears that AXOS prebiotics exert beneficial effects on SCFA production and bacterial taxa, warranting further exploration. Well controlled studies using appropriate exercise stress models and a range of well validated EIGS markers would determine whether these changes indeed confer a benefit to gastrointestinal integrity and resultant systemic effects.

## Conclusion

The effect of pre-, pro- and syn-biotic supplementation, taken by healthy and active adults, on gastrointestinal outcomes at rest and in response to exercise are highly varied, however the following can be concluded: (i) Supplementation with prebiotic ingredients appears to alter the gut bacterial microbiota, particularly increasing the relative abundance of total *Bifidobacterium;* (ii) supplementation with probiotics usually results in an increase in the relative abundance of the supplemented species and/or strain, however the effect on other bacterial types is inconsistent and may be specific to the supplement chosen; (iii) Pre-, pro- and syn-biotic supplements do not significantly change bacterial α-diversity, as determined by Shannon index, Simpson index or 16S gene sequencing; (iv) supplementation with AXOS prebiotic ingredients, appears to increase fecal SCFA content at rest; (v) both pre- and pro-biotic supplements do not appear to significantly influence intestinal injury and permeability, systemic endotoxin and inflammatory cytokine responses, or GIS at rest, and have minimal impact on gastrointestinal motility and function at rest in otherwise, healthy, active adults, with the exception of gastric emptying which may be delayed (i.e., slower) with inulin supplementation; (vi) probiotic supplementation with the species studied to date do not substantially influence intestinal injury and permeability, and subsequent systemic endotoxin or inflammatory cytokine responses, or GIS in response to exercise, although many studies lack adequate exertional stress or heat stress, or appropriate biomarkers, to definitively make this conclusion; (vii) currently no studies have investigated the effect of prebiotic supplements on gastrointestinal responses to exercise; (viii) synbiotic supplements appear to more closely resemble the effects of probiotic than prebiotic supplements, due to the generally very small quantity of prebiotic ingredients included in them; (ix) the choice of supplements studied to date appears to lack a logical, evidence-based approach to finding the ideal prebiotic ingredient and/or probiotic strain/s, based on existing mechanistic or observational studies of gut microbiota and EIGS outcomes. Therefore, the above conclusions may reflect poor choice of supplement ingredients rather than a failure of pre-, pro- or syn-biotic products in general. In addition to a more evidence-based approach to ingredient selection, research methodologies, including biomarker choice, timing of biological sampling in relation to exercise, the chosen exercise protocol and ambient conditions, may all contribute to the success or failure to find suitable pre-, pro- and syn-biotic products that improve EIGS outcomes in active adults. Future research should be designed to maximize the likelihood of exercise-associated gastrointestinal disturbance, taking biological samples immediately before and after exercise, as well as in the hours following, and utilize a complete, well-validated suite of EIGS biomarkers to ensure data is correctly interpreted.

## Data availability statement

The original contributions presented in the study are included in the article/supplementary material, further inquiries can be directed to the corresponding author.

## Author contributions

CR, ASM, and ZH undertook the systematic review (search, screening, eligibility, and data extraction as primary or secondary reviewer) and cross-checked by AJM. CR and RC contributed to the final draft preparation of the manuscript. All authors contributed to the manuscript review, read, and approved the final manuscript.

## References

[1] Aziz I Simrén M. The overlap between irritable bowel syndrome and organic gastrointestinal diseases. Lancet Gastroenterol Hepatol. (2021) 6:139–48. 10.1016/S2468-1253(20)30212-0 33189181

[2] Costa RJS Snipe RMJ Kitic CM Gibson PR. Systematic review: exercise-induced gastrointestinal syndrome-implications for health and intestinal disease. Aliment Pharmacol Ther. (2017) 46:246–65. 10.1111/apt.14157 28589631

[3] Costa RJS Gaskell SK Mccubbin AJ Snipe RMJ. Exertional-heat stress-associated gastrointestinal perturbations during Olympic sports: management strategies for athletes preparing and competing in the 2020 Tokyo Olympic Games. Temperature. (2020) 7:58–88. 10.1080/23328940.2019.1597676 32166105 PMC7053925

[4] Barrett KE. Epithelial biology in the gastrointestinal system: insights into normal physiology and disease pathogenesis. J Physiol. (2012) 590:419–20. 10.1113/jphysiol.2011.227058 22298901 PMC3379689

[5] Holzer P Farzi A Hassan AM Zenz G Jačan A Reichmann F. Visceral inflammation and immune activation stress the brain. Front Immunol. (2017) 8:1613. 10.3389/fimmu.2017.01613 29213271 PMC5702648

[6] Miall A Khoo A Rauch C Snipe RMJ Camões-Costa VL Gibson PR . Two weeks of repetitive gut-challenge reduce exercise-associated gastrointestinal symptoms and malabsorption. Scand J Med Sci Sports. (2018) 28:630–40. 10.1111/sms.12912 28508559

[7] Layer P Peschel S Schlesinger T Goebell H. Human pancreatic secretion and intestinal motility: effects of ileal nutrient perfusion. Am J Physiol. (1990) 258:G196–201. 10.1152/ajpgi.1990.258.2.G196 1689548

[8] Van Citters GW Lin HC. Ileal brake: neuropeptidergic control of intestinal transit. Curr Gastroenterol Rep. (2006) 8:367–73. 10.1007/s11894-006-0021-9 16968603

[9] Shin HS Ingram JR Mcgill AT Poppitt SD. Lipids, CHOs, proteins: can all macronutrients put a ‘brake' on eating? Physiol Behav. (2013) 120:114–23. 10.1016/j.physbeh.2013.07.008 23911804

[10] Van Avesaat M Troost FJ Ripken D Hendriks HF Masclee AA. Ileal brake activation: macronutrient-specific effects on eating behavior? Int J Obes. (2015) 39:235–43. 10.1038/ijo.2014.112 24957485

[11] Lin YM Li F Shi XZ. Mechanical stress is a pro-inflammatory stimulus in the gut: *in vitro, in vivo* and *ex vivo* evidence. PLoS ONE. (2014) 9:e106242. 10.1371/journal.pone.0106242 25180799 PMC4152012

[12] Fleshner M Crane CR. Exosomes, DAMPs and miRNA: features of stress physiology and immune homeostasis. Trends Immunol. (2017) 38:768–76. 10.1016/j.it.2017.08.002 28838855 PMC5624844

[13] Gaskell SK Lis DM Costa RJS. Exercise-induced gastrointestinal syndrome (Chapter 21, pages 551-575). In:Burke L Deakin V Minehan M. Clinical Sports Nutrition 6th, ed. Sydney, NSW: McGraw-Hill Education (2021).

[14] Perko MJ Nielsen HB Skak C Clemmesen JO Schroeder TV Secher NH. Mesenteric, coeliac and splanchnic blood flow in humans during exercise. J Physiol. (1998) 513:907–13. 10.1111/j.1469-7793.1998.907ba.x 9824727 PMC2231328

[15] Rehrer NJ Smets A Reynaert H Goes E De Meirleir K. Effect of exercise on portal vein blood flow in man. Med Sci Sports Exerc. (2001) 33:1533–7. 10.1097/00005768-200109000-00017 11528343

[16] Gill SK Teixeira A Rama L Prestes J Rosado F Hankey J . Circulatory endotoxin concentration and cytokine profile in response to exertional-heat stress during a multi-stage ultra-marathon competition. Exerc Immunol Rev. (2015) 21:114–28. 10.1111/1747 25830597

[17] Grootjans J Lenaerts K Buurman WA Dejong CH Derikx JP. Life and death at the mucosal-luminal interface: new perspectives on human intestinal ischemia-reperfusion. World J Gastroenterol. (2016) 22:2760–70. 10.3748/wjg.v22.i9.2760 26973414 PMC4777998

[18] Van Wijck K Lenaerts K Van Loon LJ Peters WH Buurman WA Dejong CH. Exercise-induced splanchnic hypoperfusion results in gut dysfunction in healthy men. PLoS ONE. (2011) 6:e22366. 10.1371/journal.pone.0022366 21811592 PMC3141050

[19] Costa RJS Miall A Khoo A Rauch C Snipe R Camões-Costa V . Gut-training: the impact of two weeks repetitive gut-challenge during exercise on gastrointestinal status, glucose availability, fuel kinetics, and running performance. Appl Physiol Nutr Metab. (2017) 42:547–57. 10.1139/apnm-2016-0453 28177715

[20] Gaskell SK Rauch CE Parr A Costa RJS. Diurnal versus nocturnal exercise-effect on the gastrointestinal tract. Med Sci Sports Exerc. (2021) 53:1056–67. 10.1249/MSS.0000000000002546 33065594

[21] Gaskell SK Burgell R Wiklendt L Dinning P Costa RJS. Does exertional heat stress impact gastrointestinal function and symptoms. J Sci Med Sport. (2022). 10.1016/j.jsams.2022.10.008. In press. 36347748

[22] Bermon S Castell LM Calder PC Bishop NC Blomstrand E Mooren FC . Consensus statement immunonutrition and exercise. Exerc Immunol Rev. (2017) 23:8–50. 28224969

[23] Francavilla VC Bongiovanni T Todaro L Di Pietro V Francavilla G. Probiotic supplements and athletic performance: a review of the literature. Medicina Dello Sport. (2017) 70, 247–59. 10.23736/S0025-7826.17.03037-X35276980

[24] Jäger R Mohr AE Carpenter KC Kerksick CM Purpura M Moussa A . international society of sports nutrition position stand: probiotics. J Int Soc Sports Nutr. (2019) 16:62. 10.1186/s12970-019-0329-0 31864419 PMC6925426

[25] Leite GSF Resende Master Student AS West NP Lancha AH Jr. Probiotics and sports: a new magic bullet? Nutrition. (2019) 60:152–60. 10.1016/j.nut.2018.09.023 30590242

[26] Mach N Fuster-Botella D. Endurance exercise and gut microbiota: a review. J Sport Health Sci. (2017) 6:179–97. 10.1016/j.jshs.2016.05.001 30356594 PMC6188999

[27] Pyne DB West NP Cox AJ Cripps AW. Probiotics supplementation for athletes - clinical and physiological effects. Eur J Sport Sci. (2015) 15:63–72. 10.1080/17461391.2014.971879 25339255

[28] Sivamaruthi BS Kesika P Chaiyasut C. Effect of probiotics supplementations on health status of athletes. Int J Environ Res Public Health. (2019) 16:4469. 10.3390/ijerph16224469 31766303 PMC6888046

[29] West NP Pyne DB Peake JM Cripps AW. Probiotics, immunity and exercise: a review. Exerc Immunol Rev. (2009) 15:107–26. 19957873

[30] Guo Y-T Peng Y-C Yen H-Y Wu J-C Hou W-H. Effects of probiotic supplementation on immune and inflammatory markers in athletes: a meta-analysis of randomized clinical trials. Medicina. (2022) 58:1188. 10.3390/medicina58091188 36143865 PMC9505795

[31] Heimer M Teschler M Schmitz B Mooren FC. Health benefits of probiotics in spot and exercise- Non-existent or a matter of heterogeneity? A systematic review. Front Nutr. (2022) 9:804046. 10.3389/fnut.2022.804046 35284446 PMC8906887

[32] Möller GB Da Cunha Goulart MJV Nicoletto BB Alves FD Schneider CD. Supplementation of probiotics and its effects on physically active individuals and athletes: systematic review. Int J Sport Nutr Exerc Metab. (2019) 29:481–92. 10.1123/ijsnem.2018-0227 30676130

[33] Bennett CJ Henry R Snipe RMJ Costa RJS. Is the gut microbiota bacterial abundance and composition associated with intestinal epithelial injury, systemic inflammatory profile, and gastrointestinal symptoms in response to exertional-heat stress? J Sci Med Sport. (2020) 23:1141–53. 10.1016/j.jsams.2020.06.002 32620352

[34] Gaskell SK Gill P Muir J Henry R Costa RJS. The impact of a 24h low and high FODMAP diet on faecal and plasma short chain fatty acid concentration, and its influence on markers of exercise-induced gastrointestinal syndrome in response to exertionalheat stress. Nutr Diet. (2021) 78:8. 10.1111/1747-0080.12709

[35] Gaskell SK Taylor B Muir J Costa RJS. Impact of 24-h high and low fermentable oligo-, di-, monosaccharide, and polyol diets on markers of exercise-induced gastrointestinal syndrome in response to exertional heat stress. Appl Physiol Nutr Metab. (2020) 45:569–80. 10.1139/apnm-2019-0187 31652404

[36] Young P Russo I Gill P Muir J Henry R Davidson Z . The impact of pre-exercise faecal and plasma SCFA concentration on markers of gastrointestinal integrity in response to 2h of high-intensity interval training. Nutr Diet. (2021) 78:9.

[37] Matheson PJ Wilson MA Garrison RN. Regulation of intestinal blood flow. J Surg Res. (2000) 93:182–96. 10.1006/jsre.2000.5862 10945962

[38] Snipe RMJ Khoo A Kitic CM Gibson PR Costa RJS. Carbohydrate and protein intake during exertional heat stress ameliorates intestinal epithelial injury and small intestine permeability. Appl Physiol Nutr Metab. (2017) 42:1283–92. 10.1139/apnm-2017-0361 28777927

[39] Karczewski J Troost FJ Konings I Dekker J Kleerebezem M Brummer RJ . Regulation of human epithelial tight junction proteins by Lactobacillus plantarum *in vivo* and protective effects on the epithelial barrier. Am J Physiol Gastrointest Liver Physiol. (2010) 298:G851–859. 10.1152/ajpgi.00327.2009 20224007

[40] Mujagic Z De Vos P Boekschoten MV Govers C Pieters HH De Wit NJ . The effects of *Lactobacillus plantarum* on small intestinal barrier function and mucosal gene transcription; a randomized double-blind placebo controlled trial. Sci Rep. (2017) 7:40128. 10.1038/srep40128 28045137 PMC5206730

[41] Cani PD. Human gut microbiome: hopes, threats and promises. Gut. (2018) 67:1716–25. 10.1136/gutjnl-2018-316723 29934437 PMC6109275

[42] Gilbert JA Blaser MJ Caporaso JG Jansson JK Lynch SV Knight R. Current understanding of the human microbiome. Nat Med. (2018) 24:392–400. 10.1038/nm.4517 29634682 PMC7043356

[43] Sekirov I Russell SL Antunes LC Finlay BB. Gut microbiota in health and disease. Physiol Rev. (2010) 90:859–904. 10.1152/physrev.00045.2009 20664075

[44] Costa RJS Young P Gill SK Snipe RMJ Gaskell SK Russo I . Assessment of exercise-associated gastrointestinal perturbations in research and practical settings: methodological concerns and recommendations for best practice. Int J Sports Nutr Exerc Metab. (2022) 32:387–418. 10.1123/ijsnem.2022-0048 35963615

[45] Russo F Clemente C Linsalata M Chiloiro M Orlando A Marconi E . Effects of a diet with inulin-enriched pasta on gut peptides and gastric emptying rates in healthy young volunteers. Eur J Nutr. (2011) 50:271–7. 10.1007/s00394-010-0135-6 20938778

[46] Costa RJS Camões-Costa V Snipe RMJ Dixon D Russo I Huschtscha Z. Impact of exercise-induced hypohydration on gastrointestinal integrity, function, symptoms, and systemic endotoxin and inflammatory profile. J Appl Physiol (1985). (2019) 126:1281–91. 10.1152/japplphysiol.01032.2018 30896356

[47] Russo I Della Gatta PA Garnham A Porter J Burke LM Costa RJS. Assessing overall exercise recovery processes using carbohydrate and carbohydrate-protein containing recovery beverages. Front Physiol. (2021) 12:628863. 10.3389/fphys.2021.628863 33613323 PMC7890126

[48] Russo I Della Gatta PA Garnham A Porter J Burke LM Costa RJS. Does the nutritional composition of a dairy-based recovery beverage influence post-exercise gastrointestinal and immune status, and subsequent markers of recovery optimisation in response to high intensity interval exercise? Front Nutri. (2021) 7:622270. 10.3389/fnut.2020.622270 33521041 PMC7840831

[49] Russo I Della Gatta PA Garnham A Porter J Burke LM Costa RJS. The effects of an acute “train-low” nutritional protocol on markers of recovery optimization in endurance-trained male athletes. Int J Sports Physiol Perf. (2021) 16:1764–76. 10.1123/ijspp.2020-0847 34044369

[50] Hamad A Fragkos KC Forbes A. A systematic review and meta-analysis of probiotics for the management of radiatic induced bowel disease. Clin Nutr. (2013) 32:353–60. 10.1016/j.clnu.2013.02.004 23453637

[51] Page MJ McKenzie JE Bossuyt PM Boutron I Hoffmann TC Mulrow CD . The PRISMA 2020 statement: an updated guideline for reporting systematic reviews. System Rev. (2021) 10:1–11. 10.31222/osf.io/v7gm2 33781348 PMC8008539

[52] Liberati A Altman DG Tetzlaff J Mulrow C Gøtzsche PC Ioannidis JP . The PRISMA statement for reporting systematic reviews and meta-analyses of studies that evaluate health care interventions: explanation and elaboration. PLoS Med. (2009) 6:e1000100. 10.1371/journal.pmed.1000100 19621070 PMC2707010

[53] Rohatgi A. WebPlotDigitizer. Web Based Tool to Extract Data from Plots, Images, and Maps. (2022). Available online at: https://automeris.io/WebPlotDigitizer/ (accessed November 14, 2022).

[54] Higgins J. Cochrane Handbook for Systematic Reviews of Interventions. Version 5.1. 0 [updated March 2011]. The Cochrane Collaboration (2011). Available online at: www.cochrane-handbook.org (accessed March 31, 2022).

[55] Damen B Cloetens L Broekaert WF François I Lescroart O Trogh I . Consumption of breads containing *in situ*-produced arabinoxylan oligosaccharides alters gastrointestinal effects in healthy volunteers. J Nutr. (2012) 142:470–7. 10.3945/jn.111.146464 22298569

[56] Finegold SM Li Z Summanen PH Downes J Thames G Corbett K . Xylooligosaccharide increases bifidobacteria but not lactobacilli in human gut microbiota. Food Funct. (2014) 5:436–45. 10.1039/c3fo60348b 24513849

[57] François IE Lescroart O Veraverbeke WS Marzorati M Possemiers S Evenepoel P . Effects of a wheat bran extract containing arabinoxylan oligosaccharides on gastrointestinal health parameters in healthy adult human volunteers: a double-blind, randomised, placebo-controlled, cross-over trial. Br J Nutr. (2012) 108:2229–42. 10.1017/S0007114512000372 22370444

[58] Cloetens L Broekaert WF Delaedt Y Ollevier F Courtin CM Delcour JA . Tolerance of arabinoxylan-oligosaccharides and their prebiotic activity in healthy subjects: a randomised, placebo-controlled cross-over study. Br J Nutr. (2010) 103:703–13. 10.1017/S0007114509992248 20003568

[59] Kleessen B Schwarz S Boehm A Fuhrmann H Richter A Henle T . Jerusalem artichoke and chicory inulin in bakery products affect faecal microbiota of healthy volunteers. Br J Nutr. (2007) 98:540–9. 10.1017/S0007114507730751 17445348

[60] Reimer RA Soto-Vaca A Nicolucci AC Mayengbam S Park H Madsen KL . Effect of chicory inulin-type fructan-containing snack bars on the human gut microbiota in low dietary fiber consumers in a randomized crossover trial. Am J Clin Nutr. (2020) 111:1286–96. 10.1093/ajcn/nqaa074 32320024

[61] Russo F Riezzo G Chiloiro M De Michele G Chimienti G Marconi E . Metabolic effects of a diet with inulin-enriched pasta in healthy young volunteers. Curr Pharm Des. (2010) 16:825–31. 10.2174/138161210790883570 20388093

[62] Russo F Linsalata M Clemente C Chiloiro M Orlando A Marconi E . Inulin-enriched pasta improves intestinal permeability and modifies the circulating levels of zonulin and glucagon-like peptide 2 in healthy young volunteers. Nutr Res. (2012) 32:940–6. 10.1016/j.nutres.2012.09.010 23244539

[63] Axelrod CL Brennan CJ Cresci G Paul D Hull M Fealy CE . UCC118 supplementation reduces exercise-induced gastrointestinal permeability and remodels the gut microbiome in healthy humans. Physiol Rep. (2019) 7:e14276. 10.14814/phy2.14276 31758610 PMC6874782

[64] Karhu ERA Forsgard L Alanko H Alfthan P Pussinen E . Exercise and gastrointestinal symptoms: running-induced changes in intestinal permeability and markers of gastrointestinal function in asymptomatic and symptomatic runners. Eur J Appl Physiol. (2017) 117:2519–26. 10.1007/s00421-017-3739-1 29032392 PMC5694518

[65] Batatinha H Tavares-Silva E Leite GSF Resende AS Albuquerque JAT Arslanian S . Probiotic supplementation in marathonists and its impact on lymphocyte population and function after a marathon: a randomized placebo-controlled double-blind study. Sci Rep. (2020) 10:18777. 10.1038/s41598-020-75464-0 33139757 PMC7608678

[66] Burton KJ Rosikiewicz M Pimentel G Bütikofer U Von Ah U Voirol MJ . Probiotic yogurt and acidified milk similarly reduce postprandial inflammation and both alter the gut microbiota of healthy, young men. Br J Nutr. (2017) 117:1312–22. 10.1017/S0007114517000885 28558854

[67] Carbuhn AF Reynolds SM Campbell CW Bradford LA Deckert JA Kreutzer A . Effects of probiotic (*Bifidobacterium longum* 35624) supplementation on exercise performance, immune modulation, and cognitive outlook in division I female swimmers. Sports. (2018) 6:116. 10.3390/sports6040116 30308984 PMC6315752

[68] Gill SK Allerton DM Ansley-Robson P Hemmings K Cox M Costa RJ. Does short-term high dose probiotic supplementation containing lactobacillus casei attenuate exertional-heat stress induced endotoxaemia and cytokinaemia? Int J Sport Nutr Exerc Metab. (2016) 26:268–75. 10.1123/ijsnem.2015-0186 26568577

[69] Gill SK Hankey J Wright A Marczak S Hemming K Allerton DM . The impact of a 24-h ultra-marathon on circulatory endotoxin and cytokine profile. Int J Sports Med. (2015) 36:688–95. 10.1055/s-0034-1398535 25941924

[70] Gleeson M Bishop NC Oliveira M Tauler P. Daily probiotic's (*Lactobacillus casei* Shirota) reduction of infection incidence in athletes. Int J Sport Nutr Exerc Metab. (2011) 21:55–64. 10.1123/ijsnem.21.1.55 21411836

[71] Neville V Gleeson M Folland JP. Salivary IgA as a risk factor for upper respiratory infections in elite professional athletes. Med Sci Sports Exerc. (2008) 40:1228–36. 10.1249/MSS.0b013e31816be9c3 18580401

[72] Haywood BA Black KE Baker D Mcgarvey J Healey P Brown RC. Probiotic supplementation reduces the duration and incidence of infections but not severity in elite rugby union players. J Sci Med Sport. (2014) 17:356–60. 10.1016/j.jsams.2013.08.004 24045086

[73] Hoffman JR Hoffman MW Zelicha H Gepner Y Willoughby DS Feinstein U . The effect of 2 weeks of inactivated probiotic bacillus coagulans on endocrine, inflammatory, and performance responses during self-defense training in soldiers. J Strength Cond Res. (2019) 33:2330–7. 10.1519/JSC.0000000000003265 31306390

[74] Huang WC Pan CH Wei CC Huang HY. *Lactobacillus plantarum* PS128 improves physiological adaptation and performance in triathletes through gut microbiota modulation. Nutrients. (2020) 12:2315. 10.3390/nu12082315 32752178 PMC7468698

[75] Klein A Friedrich U Vogelsang H Jahreis G. *Lactobacillus acidophilus* 74-2 and *Bifidobacterium animalis* subsp lactis DGCC 420 modulate unspecific cellular immune response in healthy adults. Eur J Clin Nutr. (2008) 62:584–93. 10.1038/sj.ejcn.1602761 17440520

[76] Lamprecht M Bogner S Schippinger G Steinbauer K Fankhauser F Hallstroem S . Probiotic supplementation affects markers of intestinal barrier, oxidation, and inflammation in trained men; a randomized, double-blinded, placebo-controlled trial. J Int Soc Sports Nutr. (2012) 9:45. 10.1186/1550-2783-9-45 22992437 PMC3465223

[77] Lee MC Jhang WL Lee CC Kan NW Hsu YJ Ho CS . The effect of kefir supplementation on improving human endurance exercise performance and antifatigue. Metabolites. (2021) 11:136. 10.3390/metabo11030136 33669119 PMC7996501

[78] Lin CL Hsu YJ Ho HH Chang YC Kuo YW Yeh YT . *Bifidobacterium longum* subsp. longum OLP-01 supplementation during endurance running training improves exercise performance in middle- and long-distance runners: a double-blind controlled trial. Nutrients. (2020) 12:1972. 10.3390/nu12071972 32630786 PMC7400043

[79] Pugh JN Sparks AS Doran DA Fleming SC Langan-Evans C Kirk B . Four weeks of probiotic supplementation reduces GI symptoms during a marathon race. Eur J Appl Physiol. (2019) 119:1491–501. 10.1007/s00421-019-04136-3 30982100 PMC6570661

[80] Sánchez Macarro M Ávila-Gandía V Pérez-Piñero S Cánovas F García-Muñoz AM Abellán-Ruiz MS . Antioxidant effect of a probiotic product on a model of oxidative stress induced by high-intensity and duration physical exercise. Antioxidants. (2021) 10:323. 10.3390/antiox10020323 33671691 PMC7926771

[81] Krotkiewski M Brzezinska Z. Lipid peroxides production after strenuous exercise and in relation to muscle morphology and capillarization. Muscle Nerve. (1996) 19:1530. 8941266 10.1002/(SICI)1097-4598(199612)19:12<1530::AID-MUS2>3.0.CO;2-B

[82] Schreiber C Tamir S Golan R Weinstein A Weinstein Y. The effect of probiotic supplementation on performance, inflammatory markers and gastro-intestinal symptoms in elite road cyclists. J Int Soc Sports Nutr. (2021) 18:36. 10.1186/s12970-021-00432-6 34001168 PMC8127283

[83] Smarkusz-Zarzecka J Ostrowska L Leszczyńska J Orywal K Cwalina U Pogodziński D. Analysis of the impact of a multi-strain probiotic on body composition and cardiorespiratory fitness in long-distance runners. Nutrients. (2020) 12:3758. 10.3390/nu12123758 33297458 PMC7762398

[84] Son J Jang LG Kim BY Lee S Park H. The effect of athletes' probiotic intake may depend on protein and dietary fiber intake. Nutrients. (2020) 12:2947. 10.3390/nu12102947 32992898 PMC7650591

[85] Strasser B Geiger D Schauer M Gostner JM Gatterer H Burtscher M . Probiotic supplements beneficially affect tryptophan-kynurenine metabolism and reduce the incidence of upper respiratory tract infections in trained athletes: a randomized, double-blinded, placebo-controlled Trial. Nutrients. (2016) 8:752. 10.3390/nu8110752 27886064 PMC5133134

[86] Areces F González-Millán C Salinero JJ Abian-Vicen J Lara B Gallo-Salazar C . Changes in serum free amino acids and muscle fatigue experienced during a half-ironman triathlon. PLoS ONE. (2015) 10:e0138376. 10.1371/journal.pone.0138376 26372162 PMC4570672

[87] Tavares-Silva E Caris AV Santos SA Ravacci GR Thomatieli-Santos RV. Effect of multi-strain probiotic supplementation on URTI symptoms and cytokine production by monocytes after a marathon race: a randomized, double-blind, placebo study. Nutrients. (2021) 13:1478. 10.3390/nu13051478 33925633 PMC8146695

[88] Townsend JR Bender D Vantrease WC Sapp PA Toy AM Woods CA . Effects of probiotic (*Bacillus subtilis* DE111) supplementation on immune function, hormonal status, and physical performance in division I baseball players. Sports. (2018) 6:70. 10.3390/sports6030070 30049931 PMC6162611

[89] Vaisberg M Paixão V Almeida EB Santos JMB Foster R Rossi M . Daily intake of fermented milk containing *Lactobacillus casei* Shirota (Lcs) modulates systemic and upper airways immune/inflammatory responses in marathon runners. Nutrients. (2019) 11:1678. 10.3390/nu11071678 31336570 PMC6682935

[90] West NP Pyne DB Cripps AW Hopkins WG Eskesen DC Jairath A . *Lactobacillus fermentum* (PCC^®^) supplementation and gastrointestinal and respiratory-tract illness symptoms: a randomised control trial in athletes. Nutr J. (2011) 10:30. 10.1186/1475-2891-10-30 21477383 PMC3083335

[91] Fricker PA Pyne DB Saunders PU Cox AJ Gleeson M Telford RD. Influence of training loads on patterns of illness in elite distance runners. Clin J Sport Med. (2005) 15:246–52. 10.1097/01.jsm.0000168075.66874.3e 16003039

[92] Coman M Verdenelli M Silvi S Cecchini C Gabbianelli R Amadio E . Knowledge and acceptance of functional foods: a preliminary study on influence of a synbiotic fermented milk on athlete health. Int J Probiot Prebiot. (2017) 12:33–42.

[93] Quero CD Manonelles P Fernández M Abellán-Aynés O López-Plaza D Andreu-Caravaca L . Differential health effects on inflammatory, immunological and stress parameters in professional soccer players and sedentary individuals after consuming a synbiotic. A triple-blinded, randomized, placebo-controlled pilot study. Nutrients. (2021) 13:1321. 10.3390/nu13041321 33923663 PMC8073688

[94] Roberts JD Suckling CA Peedle GY Murphy JA Dawkins TG Roberts MG. An exploratory investigation of endotoxin levels in novice long distance triathletes, and the effects of a multi-strain probiotic/prebiotic, antioxidant intervention. Nutrients. (2016) 8:733. 10.3390/nu8110733 27869661 PMC5133117

[95] Faul F Erdfelder E Lang AG Buchner A. G^*^ Power 3: a flexible statistical power analysis program for the social, behavioral, and biomedical sciences. Behav Res Methods. (2007) 39:175–91. 10.3758/BF03193146 17695343

[96] Valle MCPR Vieira IA Fino LC Gallina DA Esteves AM Da Cunha DT . Immune status, well-being and gut microbiota in military supplemented with synbiotic ice cream and submitted to field training: a randomised clinical trial. Br J Nutr. (2021) 126:1794–808. 10.1017/S0007114521000568 33593462

[97] Olivares M Díaz-Ropero MP Gomez N Lara-Villoslada F Sierra S Maldonado JA . The consumption of two new probiotic strains, *Lactobacillus gasseri* CECT 5714 and *Lactobacillus coryniformis* CECT 5711, boosts the immune system of healthy humans. Int Microbiol. (2006) 9:47–52. 10.1016/j.ijfoodmicro.2005.08.019 16636989

[98] West NP Pyne DB Cripps AW Christophersen CT Conlon MA Fricker PA. Gut Balance, a synbiotic supplement, increases fecal Lactobacillus paracasei but has little effect on immunity in healthy physically active individuals. Gut Microbes. (2012) 3:221–7. 10.4161/gmic.19579 22572834 PMC3427214

[99] Kekkonen RA Vasankari TJ Vuorimaa T Haahtela T Julkunen I Korpela R. The effect of probiotics on respiratory infections and gastrointestinal symptoms during training in marathon runners. Int J Sport Nutr Exerc Metab. (2007) 17:352–63. 10.1123/ijsnem.17.4.352 17962710

[100] Pugh JN Wagenmakers AJM Doran DA Fleming SC Fielding BA Morton JP . Probiotic supplementation increases carbohydrate metabolism in trained male cyclists: a randomized, double-blind, placebo-controlled crossover trial. Am J Physiol Endocrinol Metab. (2020) 318:E504–e513. 10.1152/ajpendo.00452.2019 32069071

[101] Yeo SE Jentjens RL Wallis GA Jeukendrup AE. Caffeine increases exogenous carbohydrate oxidation during exercise. J Appl Physiol. (2005) 99:844–50. 10.1152/japplphysiol.00170.2005 15831802

[102] Shing CM Peake JM Lim CL Briskey D Walsh NP Fortes MB . Effects of probiotics supplementation on gastrointestinal permeability, inflammation and exercise performance in the heat. Eur J Appl Physiol. (2014) 114:93–103. 10.1007/s00421-013-2748-y 24150782

[103] Lambert GP Broussard LJ Mason BL Mauermann WJ Gisolfi CV. Gastrointestinal permeability during exercise: effects of aspirin and energy-containing beverages. J Appl Physiol (1985). (2001) 90:2075–80. 10.1152/jappl.2001.90.6.2075 11356768

[104] Marchbank T Davison G Oakes JR Ghatei MA Patterson M Moyer MP . The nutriceutical bovine colostrum truncates the increase in gut permeability caused by heavy exercise in athletes. Am J Physiol-Gastrointest Liver Physiol. (2011) 300:G477–84. 10.1152/ajpgi.00281.2010 21148400

[105] Lim CL Pyne D Horn P Kalz A Saunders P Peake J . The effects of increased endurance training load on biomarkers of heat intolerance during intense exercise in the heat. Appl Physiol Nutr Metab. (2009) 34:616–24. 10.1139/H09-021 19767796

[106] Hoffman MD Snipe RMJ Costa RJS. Ad libitum drinking adequately supports hydration during 2 h of running in different ambient temperatures. Euro J Appl Physiol. (2018) 118:2687–97. 10.1007/s00421-018-3996-7 30267225

[107] Dill DB Costill DL. Calculation of percentage changes in volumes of blood, plasma, and red cells in dehydration. J Appl Physiol. (1974) 37:247–8. 10.1152/jappl.1974.37.2.247 4850854

[108] Grames C Berry-Cabán CS. Ischemic colitis in an endurance runner. Case Rep Gastrointest Med. (2012) 2012:356895. 10.1155/2012/356895 23091744 PMC3474219

[109] Snipe RMJ Khoo A Kitic CM Gibson PR Costa RJS. The impact of exertional-heat stress on gastrointestinal integrity, gastrointestinal symptoms, systemic endotoxin and cytokine profile. Eur J Appl Physiol. (2018) 118:389–400. 10.1007/s00421-017-3781-z 29234915

[110] Snipe RMJ Costa RJS. Does biological sex impact intestinal epithelial injury, small intestine permeability, gastrointestinal symptoms and systemic cytokine profile in response to exertional-heat stress? J Sports Sci. (2018) 36:2827–35. 10.1080/02640414.2018.1478612 29790452

[111] Al-Saffar AK Meijer CH Gannavarapu VR Hall G Li Y Diaz Tartera HO . Parallel changes in harvey-bradshaw index, TNFα, and intestinal fatty acid binding protein in response to infliximab in Crohn's disease. Gastroenterol Res Pract. (2017) 2017:1745918. 10.1155/2017/1745918 29201046 PMC5672611

[112] Haas V Büning C Buhner S Von Heymann C Valentini L Lochs H. Clinical relevance of measuring colonic permeability. Eur J Clin Invest. (2009) 39:139–44. 10.1111/j.1365-2362.2008.02075.x 19200167

[113] Jekarl DW Kim JY Ha JH Lee S Yoo J Kim M . Diagnosis and prognosis of sepsis based on use of cytokines, chemokines, and growth factors. Dis Markers. (2019) 2019:1089107. 10.1155/2019/1089107 31583025 PMC6754872

[114] Linsalata M Riezzo G D'attoma B Clemente C Orlando A Russo F. Noninvasive biomarkers of gut barrier function identify two subtypes of patients suffering from diarrhoea predominant-IBS: a case-control study. BMC Gastroenterol. (2018) 18:167. 10.1186/s12876-018-0888-6 30400824 PMC6219148

[115] Martinez-Fierro ML Garza-Veloz I Rocha-Pizaña MR Cardenas-Vargas E Cid-Baez MA Trejo-Vazquez F . Serum cytokine, chemokine, and growth factor profiles and their modulation in inflammatory bowel disease. Medicine. (2019) 98:e17208. 10.1097/MD.0000000000017208 31567972 PMC6756690

[116] Pelsers MM Hermens WT Glatz JF. Fatty acid-binding proteins as plasma markers of tissue injury. Clin Chim Acta. (2005) 352:15–35. 10.1016/j.cccn.2004.09.001 15653098

[117] Power N Turpin W Espin-Garcia O Smith MI Croitoru K. Serum zonulin measured by commercial kit fails to correlate with physiologic measures of altered gut permeability in first degree relatives of Crohn's disease patients. Front Physiol. (2021) 12:645303. 10.3389/fphys.2021.645303 33841181 PMC8027468

[118] Surbatovic M Veljovic M Jevdjic J Popovic N Djordjevic D Radakovic S. Immunoinflammatory response in critically ill patients: severe sepsis and/or trauma. Mediators Inflamm. (2013) 2013:362793. 10.1155/2013/362793 24371374 PMC3859159

[119] Snipe RMJ Costa RJS. Does the temperature of water ingested during exertional-heat stress influence gastrointestinal injury, symptoms, and systemic inflammatory profile? J Sci Med Sport. (2018) 21:771–6. 10.1016/j.jsams.2017.12.014 29371075

[120] Alcock R Mccubbin A Camões-Costa V Costa RJS. Case study: providing nutritional support to an ultraendurance runner in preparation for a self-sufficient multistage ultramarathon: rationed versus full energy provisions. Wilderness Environ Med. (2018) 29:508–20. 10.1016/j.wem.2018.06.004 30249353

[121] Flood TR Montanari S Wicks M Blanchard J Sharp H Taylor L . Addition of pectin-alginate to a carbohydrate beverage does not maintain gastrointestinal barrier function during exercise in hot-humid conditions better than carbohydrate ingestion alone. Appl Physiol Nutr Metab. (2020) 45:1145–55. 10.1139/apnm-2020-0118 32365303

[122] Jonvik KL Lenaerts K Smeets JSJ Kolkman JJ VAN Loon LJC . Sucrose but Not Nitrate Ingestion Reduces Strenuous Cycling-induced Intestinal Injury. Med Sci Sports Exerc. (2019) 51:436–44. 10.1249/MSS.0000000000001800 30299412

[123] Rehrer NJ Goes E Dugardeyn C Reynaert H Demeirleir K. Effect of carbohydrate on portal vein blood flow during exercise. Int J Sports Med. (2005) 26:171–6. 10.1055/s-2004-820957 15776331

[124] March DS Marchbank T Playford RJ Jones AW Thatcher R Davison G. Intestinal fatty acid-binding protein and gut permeability responses to exercise. Eur J Appl Physiol. (2017) 117:931–41. 10.1007/s00421-017-3582-4 28290057 PMC5388720

[125] Snipe RMJ Khoo A Kitic CM Gibson PR Costa RJS. The impact of mild heat stress during prolonged running on gastrointestinal integrity, gastrointestinal symptoms, systemic endotoxin and cytokine profiles. Int J Sports Med. (2018) 39:255–63. 10.1055/s-0043-122742 29415294

[126] Scheffler L Crane A Heyne H Tönjes A Schleinitz D Ihling CH . Widely used commercial ELISA does not detect precursor of haptoglobin2, but recognizes properdin as a potential second member of the zonulin family. Front Endocrinol. (2018) 9:22. 10.3389/fendo.2018.00022 29459849 PMC5807381

[127] Ajamian M Steer D Rosella G Gibson PR. Serum zonulin as a marker of intestinal mucosal barrier function: may not be what it seems. PLoS ONE. (2019) 14:e0210728. 10.1371/journal.pone.0210728 30640940 PMC6331146

[128] Horner KM Schubert MM Desbrow B Byrne NM King NA. Acute exercise and gastric emptying: a meta-analysis and implications for appetite control. Sports Med. (2015) 45:659–78. 10.1007/s40279-014-0285-4 25398225

[129] Bate JP Irving PM Barrett JS Gibson PR. Benefits of breath hydrogen testing after lactulose administration in analysing carbohydrate malabsorption. Eur J Gastroenterol Hepatol. (2010) 22:318–26. 10.1097/MEG.0b013e32832b20e8 19636251

[130] Costa RJ Snipe R Camões-Costa V Scheer V Murray A. The impact of gastrointestinal symptoms and dermatological injuries on nutritional intake and hydration status during ultramarathon events. Sports Med Open. (2016) 2:16. 10.1186/s40798-015-0041-9 26767151 PMC4701764

[131] Bengtsson M Persson J Sjölund K Ohlsson B. Further validation of the visual analogue scale for irritable bowel syndrome after use in clinical practice. Gastroenterol Nurs. (2013) 36:188–98. 10.1097/SGA.0b013e3182945881 23732784

[132] Drossman DA. The functional gastrointestinal disorders and the Rome III process. Gastroenterol. (2006) 130:1377–90. 10.1053/j.gastro.2006.03.008 16678553

[133] Gaskell SK Snipe RMJ Costa RJS. Test-retest reliability of a modified visual analog scale assessment tool for determining incidence and severity of gastrointestinal symptoms in response to exercise stress. Int J Sport Nutr Exerc Metab. (2019) 29:411–9. 10.1123/ijsnem.2018-0215 30632417

[134] Brestoff JR Artis D. Commensal bacteria at the interface of host metabolism and the immune system. Nat Immunol. (2013) 14:676–84. 10.1038/ni.2640 23778795 PMC4013146

[135] Gnauck A Lentle RG Kruger MC. The characteristics and function of bacterial lipopolysaccharides and their endotoxic potential in humans. Int Rev Immunol. (2016) 35:189–218. 10.3109/08830185.2015.1087518 26606737

[136] Imhann F Vich Vila A Bonder MJ Fu J Gevers D Visschedijk MC . Interplay of host genetics and gut microbiota underlying the onset and clinical presentation of inflammatory bowel disease. Gut. (2018) 67:108–19. 10.1136/gutjnl-2016-312135 27802154 PMC5699972

[137] Van Der Beek CM Dejong CHC Troost FJ Masclee AM Lenaerts K. Role of short-chain fatty acids in colonic inflammation, carcinogenesis, and mucosal protection and healing. Nutr Rev. (2017) 75:286–305. 10.1093/nutrit/nuw067 28402523

[138] Wong JM De Souza R Kendall CW Emam A Jenkins DJ. Colonic health: fermentation and short chain fatty acids. J Clin Gastroenterol. (2006) 40:235–43. 10.1097/00004836-200603000-00015 16633129

[139] Sanchez JI Marzorati M Grootaert C Baran M Van Craeyveld V Courtin CM . Arabinoxylan-oligosaccharides (AXOS) affect the protein/carbohydrate fermentation balance and microbial population dynamics of the Simulator of Human Intestinal Microbial Ecosystem. Microb Biotechnol. (2009) 2:101–13. 10.1111/j.1751-7915.2008.00064.x 21261885 PMC3815425

[140] Dalile B Vervliet B Bergonzelli G Verbeke K Van Oudenhove L. Colon-delivered short-chain fatty acids attenuate the cortisol response to psychosocial stress in healthy men: a randomized, placebo-controlled trial. Neuropsychopharmacol. (2020) 45:2257–66. 10.1038/s41386-020-0732-x 32521538 PMC7784980

[141] Gibson PR Halmos EP Muir JG. Review article: FODMAPS, prebiotics and gut health-the FODMAP hypothesis revisited. Aliment Pharmacol Ther. (2020) 52:233–46. 10.1111/apt.15818 32562590

